# BrainBaseline Assessment of Cognition and Everyday Functioning (“BRACE”-ing for the Future): Establishing iPad-Based Norms for Cognitive Function in the Multicenter AIDS Cohort Study and Women’s Interagency HIV Study Combined Cohort Study

**DOI:** 10.2196/70207

**Published:** 2026-05-28

**Authors:** Leah H Rubin, Pauline M Maki, Joan Severson, Adam Lieberman, Eran F Shorer, Sabina A Haberlen, Deborah R Gustafson, Michelle Floris-Moore, Valentina Stosor, Matthew J Mimiaga, Jamie Peven, Deborah L Jones, Anandi N Sheth, Kathleen M Weber, Amanda B Spence, Anjali Sharma, David E Vance, Raha M Dastgheyb

**Affiliations:** 1 Department of Neurology, Johns Hopkins University, 600 N Wolfe St, Carnegie 303, Baltimore, MD, 21287-7613, United States, 1 443-287-0571; 2 Psychiatry and Behavioral Sciences, Johns Hopkins University School of Medicine, Baltimore, MD, United States; 3 Molecular and Comparative Pathobiology, Johns Hopkins University School of Medicine, Baltimore, MD, United States; 4 Department of Epidemiology, Johns Hopkins University Bloomberg School of Public Health, Baltimore, MD, United States; 5 Departments of Psychiatry, Psychology, and Obstetrics and Gynecology, University of Illinois Chicago, Chicago, IL, United States; 6 Human True Inc, Seattle, WA, United States; 7 Department of Neurology, State University of New York Downstate Health Sciences University, Brooklyn, NY, United States; 8 Department of Medicine, University of North Carolina at Chapel Hill, Chapel Hill, NC, United States; 9 Departments of Medicine and Surgery, Northwestern University, Evanston, IL, United States; 10 Department of Epidemiology, University of California Los Angeles Fielding School of Public Health, Los Angeles, CA, United States; 11 Department of Psychiatry and Biobehavioral Sciences, University of California Los Angeles Geffen School of Medicine, Los Angeles, CA, United States; 12 Department of Psychiatry, University of Pittsburgh, Pittsburgh, PA, United States; 13 Behavioral Health Service Line, VA Pittsburgh Healthcare System, Pittsburgh, PA, United States; 14 Department of Psychiatry and Behavioral Sciences, Miller School of Medicine, University of Miami, Miami, FL, United States; 15 Department of Medicine, Emory University, Atlanta, GA, United States; 16 Hektoen Institute, Chicago, IL, United States; 17 Department of Medicine, Georgetown University, Washington, DC, United States; 18 Department of Medicine, Albert Einstein College of Medicine, Bronx, NY, United States; 19 Department of Acute, Chronic and Continuing Care, University of Alabama at Birmingham, Birmingham, AL, United States

**Keywords:** digital health, cognition, human immunodeficiency virus, HIV, normative data, regression models, tablet-based testing

## Abstract

**Background:**

Digital cognitive assessments are increasingly used in large-scale studies to assess brain health, offering scalable, standardized, and self-directed testing solutions. Cognitive function remains a concern for people with HIV despite antiretroviral therapy. The BRACE (BrainBaseline Assessment of Cognition and Everyday Functioning) is a validated tablet-based screener for cognition in people with HIV. Preliminary pilot norms were established in a small sample (n=144), but full regression-based normative data have not yet been developed. Consequently, HIV serostatus differences based on standardized BRACE scores and cognitive correlates have not been systematically examined.

**Objective:**

This study aims to develop regression-based normative data for BRACE performance in people without HIV who were demographically and behaviorally comparable to people with HIV within biological sex; to examine differences in cognitive performance by HIV status and biological sex; and to evaluate sociodemographic, behavioral, and clinical correlates of BRACE performance.

**Methods:**

A total of 2937 participants (1063 people without HIV [499 women] and 1874 people with HIV [1053 women]) in the Multicenter AIDS Cohort Study/Women’s Interagency HIV Study Combined Cohort Study completed BRACE once between November 2020 and March 2025. BRACE includes the Trail Making Test (A and B), Stroop-Color, and visual spatial learning. Regression-based norms were derived from people without HIV using multiple demographic models (eg, age-only, age + education, and age + education + sex). The age + education model was selected for primary analyses because it provided the best balance of interpretability, parsimony, and generalizability while avoiding race-based corrections. HIV serostatus and sex differences were examined using ANOVA and *χ*^2^ tests, with effect sizes calculated using Cohen’s *d*.

**Results:**

Cognitive performance was largely comparable between people with HIV and people without HIV across all BRACE outcome measures. Statistically significant differences were very small in magnitude (all effect sizes<0.11) and primarily observed among men on Stroop-Color. Across groups, older age and fewer years of education were associated with poorer raw BRACE performance, although these associations attenuated after demographic adjustment using T-scores. Most clinical and behavioral factors (eg, hypertension, smoking, and noncannabis substance use) were related to poorer raw scores but not standardized performance. However, diabetes and cannabis use remained independently associated with T-scores across multiple measures—diabetes with poorer scores and cannabis use with higher scores, an association that should be interpreted cautiously. HIV-specific clinical factors, such as nadir CD4 count and antiretroviral therapy duration, were linked primarily to raw scores.

**Conclusions:**

This study establishes the first regression-based normative data for BRACE, derived from a large, demographically diverse people without HIV, and demonstrates its applicability for evaluating cognitive function in people with HIV. Findings indicate minimal cognitive differences between people with HIV and people without HIV and highlight the influence of common sociodemographic and metabolic factors. These results support BRACE as a scalable, reliable, and self-administered digital tool for assessing cognitive health in diverse populations and underscore its potential for longitudinal monitoring and precision phenotyping in both research and clinical contexts.

## Introduction

Cognitive health is a central aspect of aging and chronic disease management, yet comprehensive neuropsychological assessments remain resource-intensive. Traditional paper-and-pencil neuropsychological batteries are the gold standard for assessing and staging cognitive impairment because of their reliability, validity, and established normative data. However, the staff training, cross-site standardization, and administration required for traditional neuropsychological testing limit scalability. This challenge is particularly relevant in HIV, where cognitive health remains a concern for people with HIV despite effective antiretroviral therapy (ART).

Tablet-based cognitive assessments offer scalable, standardized, self-directed testing. These tools provide automated scoring and data aggregation, reducing staff burden and enabling broader implementation. The BRACE (BrainBaseline Assessment of Cognition and Everyday Functioning, Clinical Ink, Inc) is a brief, iPad-based cognitive evaluation. In a study of 404 people with HIV receiving outpatient clinical care in Baltimore, Maryland, BRACE demonstrated excellent test-retest reliability, minimal practice effects over 30-day and 10-month intervals, strong correlations with a gold-standard neuropsychological battery, and good classification accuracy for cognitive impairment [1]. To extend its use in HIV research and clinical settings, normative data reflecting the demographic and clinical diversity of people with HIV and people without HIV are needed. Norms for people without HIV, particularly among women, may differ from those in the general population because comparison groups in HIV research often have a high prevalence of risk factors associated with cognitive dysfunction, including lower educational attainment, mental health burden, substance use, and socioeconomic disadvantage [2].

In 2019, the Multicenter AIDS Cohort Study (MACS) and Women’s Interagency HIV Study (WIHS) merged to form the MACS/WIHS Combined Cohort Study (MWCCS). Before the merger, each cohort administered comprehensive neuropsychological batteries every 24 months. During the COVID-19 pandemic, the iPad-based BRACE tool was introduced to reduce face-to-face contact and shorten testing time. Here, we present the initial cross-sectional BRACE data from the MWCCS. We developed regression-based normative data in people without HIV, examined HIV serostatus differences in cognitive performance using both raw and standardized (T-score) metrics, evaluated whether HIV serostatus differences varied by biological sex, given prior findings in this cohort [2,3], and examined additional covariates that may inform future BRACE-based analyses stratified by HIV serostatus and biological sex.

## Methods

### Participants and Data Source

In 2019, the MWCCS combined 2 long-running prospective, multicenter US sex-specific cohorts of people with HIV and people without HIV: the MACS (men) and the WIHS (women). The combined cohort also enrolled additional people with HIV and demographically similar people without HIV across 14 clinical research sites located in the Mid-Atlantic/Northeast (New York-Brooklyn and Bronx; Baltimore, MD; Washington, DC), South (Birmingham, AL; Jackson, MS, Atlanta, GA; Chapel Hill, NC; Miami, FL), Midwest (Pittsburgh, PA; Columbus, OH; Chicago, IL), and West Coast (California-San Francisco and Los Angeles). Participant characteristics have been previously described in detail [4].

Data collected at annual in-person or semiannual interim telephone study visits included physical examinations, medical and psychosocial interviews, and blood draws. Measures included sociodemographics measures (self-reported date of birth, race or ethnicity, and education), behavioral factors (substance use), clinical measures (Center for Epidemiologic Studies Depression Scale [CES-D]), and reproductive history (hysterectomy, menopause status according to the STRAW+10 [Staging of Reproductive Aging Workshop] [5]).

Participants in this cross-sectional analysis completed BRACE during their first in-person MWCCS visit between November 30, 2020, and March 28, 2025. As BRACE was only available in English, analyses were restricted to English-speaking participants.

### Ethical Considerations

The parent study, MWCCS, received institutional review board approval at each participating clinical research site, and all participants provided written informed consent. This study utilized deidentified MWCCS data provided under an MWCCS-approved concept sheet and did not involve direct participant contact. Data sharing was approved by the MWCCS Data Analysis and Coordination Center Institutional Review Board (23015/CR2949; Multimedia Appendix 1). Participants’ confidentiality was maintained through the removal of personal identifiers prior to data sharing. Compensation for participants was determined by each site as part of the parent study and was not specific to this analysis.

### Cognitive Assessments

Cognitive function was assessed using BRACE (Clinical Ink, Inc), a self-administered iPad-based tool with automated audio and video instructions. BRACE includes 4 widely used cognitive tests assessing processing speed, executive function, and visuospatial learning—Trail Making Test (TMT)-A, TMT-B, Stroop-Color, and a Visual Spatial Learning Test (VSLT; Table 1).

**Table 1.** Cognitive tests in BRACE (BrainBaseline Assessment of Cognition and Everyday Functioning).

Test	Measures	Outcomes
TMT-A^a^	Psychomotor speed	Time to completion
TMT-B	Psychomotor speed Executive function, particularly set-shifting and mental flexibility	Time to completion TMT-B–TMT-A
STROOP-Color	Psychomotor speed	Time to completion Number of accurate trials
VSLT^b^	Visuospatial learning and attention	Total correctly placed items minus incorrectly placed items

a TMT: Trail Making Test.

b VSLT: Visual Spatial Learning Test.

Raw BRACE data were transmitted from Clinical Ink and processed locally using the custom graphical user interface developed by Dastgheyb [6]. This graphical user interface parses trial-level output files, applies quality control (QC) algorithms, and generates the primary performance metrics (time to completion and/or accuracy) for each test. QC procedures were used to ensure that participants interacted with the tasks as intended, including completing more than 15 Stroop-color trials accurately and making less than 5 errors on TMT-A and less than 10 errors on TMT-B. For timed outcomes, lower raw scores indicate faster (better) performance. Normative standards (T-scores) were derived from the MWCCS people without HIV sample, with higher T-scores indicating better performance.

### Statistical Analysis

Descriptive statistics summarized sociodemographic, behavioral, and clinical characteristics by HIV serostatus and biological sex. Group differences (by HIV serostatus, sex stratified by HIV serostatus, or HIV serostatus stratified by sex) were examined using Wilcoxon rank-sum tests for continuous variables and *χ*² tests for categorical variables. Regression-based normative equations were developed in the people without HIV sample and applied to both people without HIV and people with HIV samples to generate standardized T-scores for each BRACE outcome. Analyses were then conducted using both raw BRACE performance metrics and demographically adjusted T-scores derived from the MWCCS people without HIV reference group. Raw and T-scores were examined continuously. Binary indicators of BRACE impairment were defined as T-scores below 40 (≥1 SD below the normative mean) and below 35 (≥1.5 SD below). Group differences in BRACE performance (eg, people with HIV vs people without HIV) were tested using ANOVA for continuous outcomes and *χ*² for categorical outcomes. Effect sizes for between-group differences were calculated using either Cohen’s *d* or η^2^. Effect size of approximately 0.10 was interpreted as very small. Associations between sociodemographic, behavioral, and clinical variables and BRACE performance were examined separately in people with HIV and people without HIV and further stratified by biological sex. Continuous variables were evaluated using Pearson correlations, and binary categorical variables were evaluated using point-biserial correlations. Results for variables with more than 2 categories were summarized descriptively across groups. Analyses were performed using all available data for each test. All statistical tests were 2-tailed, with significance set at *α*=.05, and analyses were conducted in R version 4.3.3 (R Foundation for Statistical Computing) and the SciDataReportR package (author RMD) [7], which now include the custom function used for the regression-based normative modeling.

## Results

### Sample Characteristics

Overall, 1874 people with HIV (1053 women) and 1063 people without HIV (499 women) from the MWCCS completed the BRACE battery at a single time point between November 30, 2020, and March 28, 2025. Compared to people without HIV, people with HIV were slightly younger, completed fewer formal years of education, were more likely to be Black or African American and women, and were less likely to use substances (Table 2). The distribution of these characteristics differed by HIV serostatus when stratified by biological sex (Table 3). Among people with HIV, the majority (1488/1602, 93%) had a viral load of <200 copies/mL and reported greater than or equal to 95% adherence to ART (1414/1607, 88%). Women with HIV were more likely to be postmenopausal and were also more likely to have had a hysterectomy compared to women without HIV.

**Table 2.** Sample characteristics by HIV status.

Characteristic	People without HIV (N=1063)	People with HIV (N=1874)	*P* value^a^
Women, n (%)	499 (47)	1053 (56)	<.001
Clinical site, n (%)			<.001
Bronx	94 (8.8)	131 (7)	
Brooklyn	78 (7.3)	201 (11)	
Washington DC	53 (5)	128 (6.8)	
San Francisco	92 (8.7)	137 (7.3)	
Chicago Cook County	48 (4.5)	131 (7)	
Chapel Hill	54 (5.1)	191 (10)	
Atlanta	91 (8.6)	206 (11)	
Miami	67 (6.3)	133 (7.1)	
Birmingham	36 (3.4)	90 (4.8)	
Jackson	43 (4)	88 (4.7)	
Baltimore	117 (11)	136 (7.3)	
Chicago Northwestern	85 (8)	128 (6.8)	
Pittsburgh/Ohio	93 (8.7)	58 (3.1)	
Los Angeles	112 (11)	116 (6.2)	
Age (y), mean (SD)	56.95 (12.34)	54.22 (10.61)	<.001
Initial study cohort, n (%)			<.001
MACS^b^	398 (40)	410 (23)	
WIHS^c^	359 (36)	826 (47)	
New MWCCS^d^ recruits	246 (25)	509 (29)	
Black/African American race	466 (44)	1042 (56)	<.001
Hispanic/Latinx ethnicity	117 (11)	230 (12)	.34
Years of education, n (%)			<.001
Less than high school	152 (14)	318 (17)	
Completed high school	201 (19)	428 (23)	
Some college	307 (29)	635 (34)	
Graduated college	142 (13)	180 (9.6)	
More than College	258 (24)	306 (16)	
CES-D^e^, mean (SD)	10.79 (10.44)	10.76 (10.61)	.96
BMI (kg/m^2^), mean (SD)	30.62 (7.85)	31.16 (8.34)	.10
Diabetes, n (%)	173 (18)	342 (21)	.14
Hypertension, n (%)	637 (68)	1139 (68)	.95
Current smoker, n (%)	240 (26)	441 (27)	.53
Ever smoker, n (%)	632 (67)	1073 (65)	.28
Current heavy alcohol use^f^, n (%)	103 (12)	155 (9.6)	.11
Recent heroin use, n (%)	314 (35)	542 (33)	.28
Recent crack use, n (%)	30 (3.4)	22 (1.4)	<.001
Recent cocaine use, n (%)	90 (10)	130 (8.1)	.08
Recent cannabis use, n (%)	90 (10)	130 (8.1)	.08
Ever cannabis use	619 (70)	1032 (63)	<.001
Ever crack, cocaine, and/or heroin use, n (%)	448 (45)	686 (40)	.008
Plasma HIV RNA viral load (copies/mL), n (%)			—^g^
≤20	—	931 (58)	
21‐200	—	557 (35)	
201‐500	—	24 (1.5)	
501‐1000	—	11 (0.7)	
>1000	—	79 (4.9)	
CD4+^h^ count (cell/mm^3^), mean (SD)	—	761 (379.49)	—
CD8+^i^ count (cell/mm^3^), mean (SD)	—	828 (433)	—
Nadir CD4+ count (cell/mm^3^), mean (SD)	—	285 (227)	—
ART^j^ adherence ≥95%, n (%)	—	1414 (88)	—
Years on ART, mean (SD)	—	17.11 (8.96)	—
HIV duration (y), mean (SD)	—	29.35 (5.77)	—

a Categorical: Pearson *χ*2 test/Fisher exact test; Continuous: Wilcoxon rank sum test.

b MACS: Multicenter AIDS Cohort Study.

c WIHS: Women’s Interagency HIV Study.

d MWCCS: MACS/WIHS Combined Cohort Study.

e CES-D: Center for Epidemiologic Studies Depression Scale.

f Heavy alcohol use is defined as ≥7 standard drinks for women and ≥17 for men per week.

g Not applicable.

h CD4: cluster of differentiation 4 count.

i CD8: cluster of differentiation 8 count.

j ART: antiretroviral therapy.

**Table 3.** Sample characteristics by HIV status and biological sex.

Characteristic	Women			Men		
	People without HIV (n=499)	People with HIV (n=1053)	*P* value^a^	People without HIV (n=464)	People with HIV (n=821)	*P* value^a^
Clinical site, n (%)			.01			<.001
Bronx	66 (13)	102 (9.7)		28 (6.3)	29 (4.2)	
Brooklyn	78 (16)	201 (19)		0 (0)	0 (0)	
Washington DC	53 (11)	127 (12)		0 (0)	1 (0.1)	
San Francisco	61 (12)	113 (11)		31 (6.9)	24 (3.5)	
Chicago Cook County	48 (9.6)	131 (12)		0 (0)	0 (0)	
Chapel Hill	38 (7.6)	94 (8.9)		16 (3.6)	97 (14)	
Atlanta	70 (14)	130 (12)		21 (4.7)	76 (11)	
Miami	44 (8.8)	51 (4.8)		23 (5.1)	82 (12)	
Birmingham	16 (3.2)	47 (4.5)		20 (4.5)	43 (6.3)	
Jackson	25 (5)	57 (5.4)		18 (4)	31 (4.5)	
Baltimore	0 (0)	0 (0)		117 (21)	136 (17)	
Chicago Northwestern	0 (0)	0 (0)		85 (19)	128 (19)	
Pittsburgh/Ohio	0 (0)	0 (0)		93 (21)	58 (8.5)	
Los Angeles	0 (0)	0 (0)		112 (25)	116 (17)	
Age (y), mean (SD)	52.96 (9.81)	54.52 (9.18)	.003	60.49 (13.25)	53.84 (12.20)	<.001
Initial study cohort			.46			<.001
MACS^b^	0 (0)	0 (0)		398 (75)	410 (57)	
WIHS^c^	336 (85)	785 (84)		0 (0)	0 (0)	
New MWCCS^d^ recruits	57 (15)	153 (16)		135 (25)	315 (43)	
Black/African American race	325 (65)	691 (66)	.93	141 (25)	351 (43)	<.001
Hispanic/Latinx ethnicity	59 (12)	112 (11)	.54	58 (10)	118 (14)	.03
Years of education, n (%)			.10			<.001
Less than high school	108 (22)	245 (23)		44 (7.8)	73 (8.9)	
Completed high school	126 (25)	256 (24)		75 (13)	172 (21)	
Some college	191 (38)	382 (36)		116 (21)	253 (31)	
Graduated college	17 (3.4)	18 (1.7)		125 (22)	162 (20)	
More than College	55 (11)	149 (14)		203 (36)	157 (19)	
CES-D^e^, mean (SD)	12.16 (10.43)	10.66 (10.40)	.03	9.23 (10.09)	10.95 (11.02)	.02
BMI (kg/m^2^), mean (SD)	32.66 (8.71)	32.90 (9.12)	.67	28.69 (6.05)	28.24 (5.97)	.20
Diabetes, n (%)	93 (26)	238 (28)	.52	86 (17)	111 (16)	.88
Hypertension, n (%)	245 (64)	613 (70)	.08	334 (68)	441 (65)	.30
Current smoker, n (%)	134 (34)	240 (27)	.01	81 (16)	171 (25)	<.001
Ever smoker, n (%)	270 (68)	581 (65)	.32	326 (66)	439 (65)	.72
Heavy alcohol use^f^, n (%)	63 (16)	104 (12)	.05	33 (7.4)	39 (5.8)	.37
Recent heroin use, n (%)	15 (3.9)	13 (1.5)	.02	161 (36)	285 (43)	.03
Recent crack use, n (%)	45 (11)	82 (9.3)	.28	15 (3.4)	9 (1.4)	.04
Recent cocaine use, n (%)	21 (5.4)	50 (5.7)	.94	40 (9.1)	57 (8.6)	.88
Recent cannabis use, n (%)	138 (35)	239 (27)	.004	40 (9.1)	57 (8.6)	.88
Ever cannabis use, n (%)	175 (42)	318 (34)	.007	352 (78)	524 (79)	>.99
Ever crack, cocaine, and heroin use, n (%)	81 (19)	144 (15)	.09	252 (48)	368 (52)	.18
STRAW^g^+10 menopause status, n (%)			<.001			—^h^
Premenopausal	98 (25)	146 (16)	—	—	—	—
Perimenopause	47 (12)	76 (8.5)	—	—	—	—
Postmenopausal	243 (63)	669 (75)	—	—	—	—
Hysterectomy	59 (15)	205 (23)	.002	—	—	—
Ever pregnant	382 (93)	859 (93)	.92	—	—	—
Plasma HIV RNA viral load (copies/mL), n (%)						
≤20	—	522 (60)	—	—	359 (56)	—
21‐200	—	284 (33)	—	—	240 (37)	—
201‐500	—	15 (1.7)	—	—	9 (1.4)	—
501‐1000	—	5 (0.6)	—	—	5 (0.8)	—
>1000	—	45 (5.2)	—	—	32 (5)	—
CD4+^i^ count (cell/mm^3^), mean (SD)	—	797 (398)	—	—	710 (343)	—
CD8+^j^ count (cell/mm^3^), mean (SD)	—	846 (465)	—	—	801 (395)	—
Nadir CD4+ count (cell/mm^3^), mean (SD)	—	284 (242)	—	—	305 (221)	—
ART^k^ adherence ≥95%, n (%)	—	743 (87)	—	—	597 (90)	—
Years on ART, mean (SD)	—	16.34 (7.98)	—	—	18.25 (10.15)	—
HIV duration (y)	—	29.60 (4.95)	—	—	28.99 (6.81)	—

a Categorical: Pearson *χ*2 test/Fisher exact test; Continuous: Wilcoxon rank sum test.

b MACS: Multicenter AIDS Cohort Study.

c WIHS: Women’s Interagency HIV Study.

d MWCCS: MACS/WIHS Combined Cohort Study.

e CES-D: Center for Epidemiologic Studies Depression Scale.

f Heavy alcohol use is defined as ≥7 standard drinks for women and ≥17 for men per week.

g STRAW+10: Staging of Reproductive Aging Workshop in women.

h Not applicable.

i CD4: cluster of differentiation 4.

j CD8: cluster of differentiation 8.

k ART: antiretroviral therapy.

### Development of BRACE Normative Data

Regression-based normative equations were generated for each raw BRACE outcome using data from people without HIV who met QC criteria on all outcomes (n=1063). Timed outcomes were converted to seconds, log-transformed, and reverse-scored, so that higher values indicated better performance across all measures. Log transformation reduced skewness in timed outcomes, whereas nontimed outcomes were already approximately normally distributed (Figure S1 in Multimedia Appendix 2).

Each outcome was regressed on age and years of education (categorized as less than high school, high school, some college, completed/graduate with a 4-year college degree, attended/completed graduate school). The resulting unstandardized beta weights for each predictor, the constant, and SE were used to compute predicted scores for each test (Table 4). Differences between predicted and observed scores (or residual scores) were divided by the SE of the estimate of the regression to generate standardized T-scores with a mean of 50 and an SD of 10. Distribution plots for the derived T-scores are shown in Figure 2 in Multimedia Appendix 2 and indicate that the normative transformation produced approximately normally distributed T-scores. These age- and education-adjusted T-scores were then applied to both people without HIV and people with HIV. A global T-score was calculated by averaging the 4 individual test T-scores (TMT-A, TMT-B, Stroop-Color time to completion, and VSLT).

**Table 4.** Regression coefficients, constants, and SEs for regression models for the normative sample of people without HIV (N=1063).

			Education (referent=completed high school)				
Outcome	Intercept	Age	Less than high school	Some college	Completed/graduated with 4-year college degree	Attended/completed graduate school	SE
TMT-A^a^	−1.066^b^	−0.004^b^	−0.026	0.065^b^	0.126^b^	0.126^b^	0.187
TMT-B	−1.474^b^	−0.004^b^	−0.013	0.075^b^	0.162^b^	0.158^b^	0.14
TMT-B–TMT-A	−19.494^b^	−0.170^b^	−0.026	3.625^c^	8.809^b^	8.645^b^	13.749
Stroop-Color duration	0.247^b^	−0.004^b^	−0.010	0.026^b^	0.057^b^	0.056^b^	0.08
Stroop-Color accuracy	36.166^b^	−0.155^b^	−0.500	1.054^c^	2.686^b^	2.784^b^	3.752
VSLT^d^	2.035^b^	−0.045^b^	−0.420	0.836^b^	1.403^b^	1.862^b^	2.453

a TMT: Trial Making Test.

b *P*<.001.

c *P*<.01.

d VSLT: Visual Spatial Learning Test.

The primary normative model included age and education because this model provided the best balance of interpretability, parsimony, and generalizability. Race or ethnicity was not included in the primary model, given longstanding concerns in neuropsychology regarding race-based corrections [8-10]. Biological sex was also not included in the primary model because the aim of this study was to evaluate HIV serostatus differences within sex using a common normative framework. Alternative normative equations were also derived using age only, age + education + biological sex, age + education + race/ethnicity, and age + education + biological sex + race/ethnicity and are presented in Tables S1-S4 in Multimedia Appendix 2, but they were not used in the primary analyses.

### BRACE Performance

Table 5 presents comparisons of cognitive performance between people with HIV and people without HIV. Overall, group differences were minimal. On Stroop-Color duration and accuracy, people with HIV showed slightly lower performance than people without HIV on standardized T-scores, although no significant differences were observed for raw scores. Effect sizes were very small (all<0.11). When examining impairment on Stroop-Color duration, significant differences were observed at the 1 SD and 1.5 SD cutoffs (*P*=.03 and *P*=.04). For accuracy, there was a trend for a difference at the 1 SD cutoff (*P*=.06). When stratified by biological sex (Table 6), the Stroop-Color difference was largely driven by men. Among women, no significant differences were detected across tests. Across both sexes, the direction and magnitude of serostatus differences were very small (all effect sizes<0.11), underscoring the generally comparable BRACE performance between people with HIV and people without HIV in this sample.

**Table 5.** Differences in cognitive performance by HIV status.

Test and outcomes	People without HIV (N=1063)	People with HIV (N=1874)	*P* value^a^	Effect size^b^
Trail Making Test (TMT)				
TMT-A				
Raw score: time to completion (ms), mean (SD)	19,490 (11,196)	19,467 (10,999)	.96	.00
T-score, mean (SD)	50 (9.98)	49.98 (9.83)	.95	.00
Impaired (<1 SD), n (%)	163 (15)	275 (15)	.68	.01
Impaired (<1.5 SD), n (%)	103 (9.7)	170 (9.1)	.63	.01
TMT-B				
Raw score: time to completion (ms), mean (SD)	44,331 (15,116)	45,404 (14,975)	.06	.07
T-score, mean (SD)	50 (9.98)	49.52 (10.06)	.21	.05
Impaired (<1 SD), n (%)	177 (17)	333 (18)	.47	.01
Impaired (<1.5 SD), n (%)	55 (5.2)	107 (5.7)	.59	.01
TMT-B−TMT-A				
Raw score, mean (SD)	24,841 (14,189)	25,966 (14,423)	.04	.08
T-score, mean (SD)	50.00 (9.98)	49.49 (10.25)	.19	.05
Impaired (<1 SD), n (%)	199 (19)	390 (21)	.19	.03
Impaired (<1.5 SD), n (%)	81 (7.6)	177 (9.5)	.10	.03
Stroop-Color				
Raw score: average response time per trial (ms), mean (SD)	879.46 (192.96)	884.98 (191.65)	.46	.03
T-score, mean (SD)	50 (9.98)	49.08 (10.14)	.02	.09
Impaired (<1 SD), n (%)	152 (14)	325 (17)	.04	.04
Impaired (<1.5 SD), n (%)	69 (6.5)	162 (8.7)	.04	.04
Raw score: % accuracy across trials, mean (SD)	28.61 (4.26)	28.38 (4.42)	.16	.05
T-score, mean (SD)	50 (9.98)	48.96 (10.86)	.009	.10
Impaired (<1 SD), n (%)	142 (13)	300 (16)	.06	.04
Impaired (<1.5 SD), n (%)	79 (7.5)	160 (8.6)	.32	.02
Visual Spatial Learning Test (VSLT)				
Raw score: correct−incorrect, mean (SD)	0.31 (2.59)	0.13 (2.54)	.07	.07
T-score, mean (SD)	50 (9.98)	49.43 (9.88)	.14	.06
Impaired (<1 SD), n (%)	179 (17)	314 (17)	>.99	.00
Impaired (<1.5 SD), n (%)	73 (6.9)	140 (7.5)	.59	.01
Global^c^				
T-score, mean (SD)	50 (6.88)	49.50 (6.87)	.06	.07
Impaired (<1 SD), n (%)	76 (7.2)	151 (8.1)	.41	.02
Impaired (<1.5 SD), n (%)	17 (1.6)	33 (1.8)	.86	.01

a Categorical: Pearson *χ*2 test/Fisher exact test; continuous: independent sample *t* test.

b Effect size: continuous: |Cohen’s *d*|; categorical: Cramer *V*.

c Global is the average of TMT-A and TMT-B time to completion, Stroop-Color time to completion, and VSLT raw score.

**Table 6.** HIV status differences in cognition in women and men separately.

Tests/outcomes	Women				Men			
	People without HIV (n=499)	People with HIV (n=1053)	*P* value^a^	Effect size^b^	People without HIV (n=564)	People with HIV (n=821)	*P* value^a^	Effect size^b^
Trail Making Test (TMT)								
TMT-A								
Raw score: time to completion (ms), mean (SD)	21,283 (13,165)	20,739 (12,278)	.44	.04	17,905 (882)	17,835 (8844)	.89	.01
T-score, mean (SD)	48.93 (11.01)	49.64 (10.60)	.23	.07	50.95 (8.87)	50.41 (8.74)	.27	.06
Impaired (<1 SD), n (%)	96 (19)	184 (18)	.43	.02	67 (12)	91 (11)	.73	.01
Impaired (<1.5 SD), n (%)	71 (14)	122 (12)	.16	.04	32 (5.7)	48 (5.9)	.97	.00
TMT-B								
Raw score: time to completion (ms), mean (SD)	46,733 (15,178)	47,258 (14,732)	.52	.04	42,206 (14,751)	43,027 (14,957)	.31	.06
T-score, mean (SD)	49.31 (9.88)	49.31 (9.90)	.99	.00	50.61 (10.03)	49.80 (10.26)	.14	.08
Impaired (<1 SD), n (%)	84 (17%)	180 (17%)	.96	.00	93 (17%)	153 (19%)	.33	.03
Impaired (<1.5 SD), n (%)	26 (5.2%)	50 (4.8%)	.79	.01	29 (5.2%)	57 (7.0%)	.21	.04
TMT-B−TMT-A								
Raw score, mean (SD)	25,450 (15,581)	26,560 (15,089)	.19	.07	24,301 (12,821)	25,205 (13,492)	.21	.07
T-score, mean (SD)	50.28 (11.14)	49.67 (10.90)	.31	.06	49.75 (8.82)	49.25 (9.35)	.31	.06
Impaired (<1 SD), n (%)	112 (23%)	235 (22%)	.99	.00	87 (15%)	155 (19%)	.11	.05
Impaired (<1.5 SD), n (%)	50 (10%)	107 (10%)	>.99	.00	31 (5.5%)	70 (8.6%)	.04	.06
Stroop-Color								
Raw score: average response time per trial (ms), mean (SD)	888.99 (190.45)	905.60 (183.70)	.10	.09	871.02 (194.93)	858.54 (198.39)	.25	.06
T-score, mean (SD)	48.87 (9.65)	48.54 (9.76)	.54	.03	51 (10.16)	49.78 (10.58)	.03	.12
Impaired (<1 SD), n (%)	78 (16%)	186 (18%)	.36	.03	74 (13%)	139 (17%)	.06	.05
Impaired (<1.5 SD), n (%)	34 (6.8)	90 (8.6)	.29	.03	35 (6.2)	72 (8.8)	.10	.05
Raw score: % accuracy across trials, mean (SD)	28.29 (4.19)	27.90 (4.21)	.09	.09	28.90 (4.29)	28.99 (4.61)	.70	.02
T-score, mean (SD)	49.01 (9.96)	48.55 (10.37)	.40	.05	50.87 (9.92)	49.48 (11.46)	.02	.13
Impaired (<1 SD), n (%)	75 (15)	175 (17)	.48	.02	67 (12)	125 (15)	.09	.05
Impaired (<1.5 SD), n (%)	41 (8.2)	83 (7.9)	.89	.01	38 (6.7)	77 (9.4)	.10	.05
Visual Spatial Learning Test (VSLT)								
Raw score: correct − incorrect, mean (SD)	−0.07 (2.60)	−0.17 (2.37)	.45	.04	0.64 (2.54)	0.51 (2.69)	.36	.05
T-score, mean (SD)	49.12 (10.11)	48.94 (9.43)	.74	.02	50.78 (9.80)	50.06 (10.40)	.19	.07
Impaired (<1 SD), n (%)	94 (19)	176 (17)	.33	.03	85 (15)	138 (17)	.42	.02
Impaired (<1.5 SD), n (%)	48 (9.7)	75 (7.1)	.11	.04	25 (4.4)	65 (8)	.01	.07
Global^c^								
T-score, mean (SD)	49.05 (6.80)	49.11 (6.71)	.89	.01	50.83 (6.85)	50.01 (7.05)	.03	.12
Impaired (<1 SD), n (%)	45 (9.1)	87 (8.3)	.68	.01	31 (5.5)	64 (7.8)	.12	.05
Impaired (<1.5 SD), n (%)	9 (1.8)	19 (1.8)	>.99	.00	8 (1.4)	14 (1.7)	.84	.01

a Categorical: Pearson *χ*2 test/Fisher exact test; continuous: independent sample *t* test.

b Effect size: continuous: |Cohen’s *d*|; categorical: Cramer *V.*

c Global is the average of TMT-A and TMT-B time to completion, Stroop-Color time to completion, and VSLT raw score.

### Sociodemographic, Clinical, and Behavioral Factors Relating to BRACE Performance

**Figure 1.**
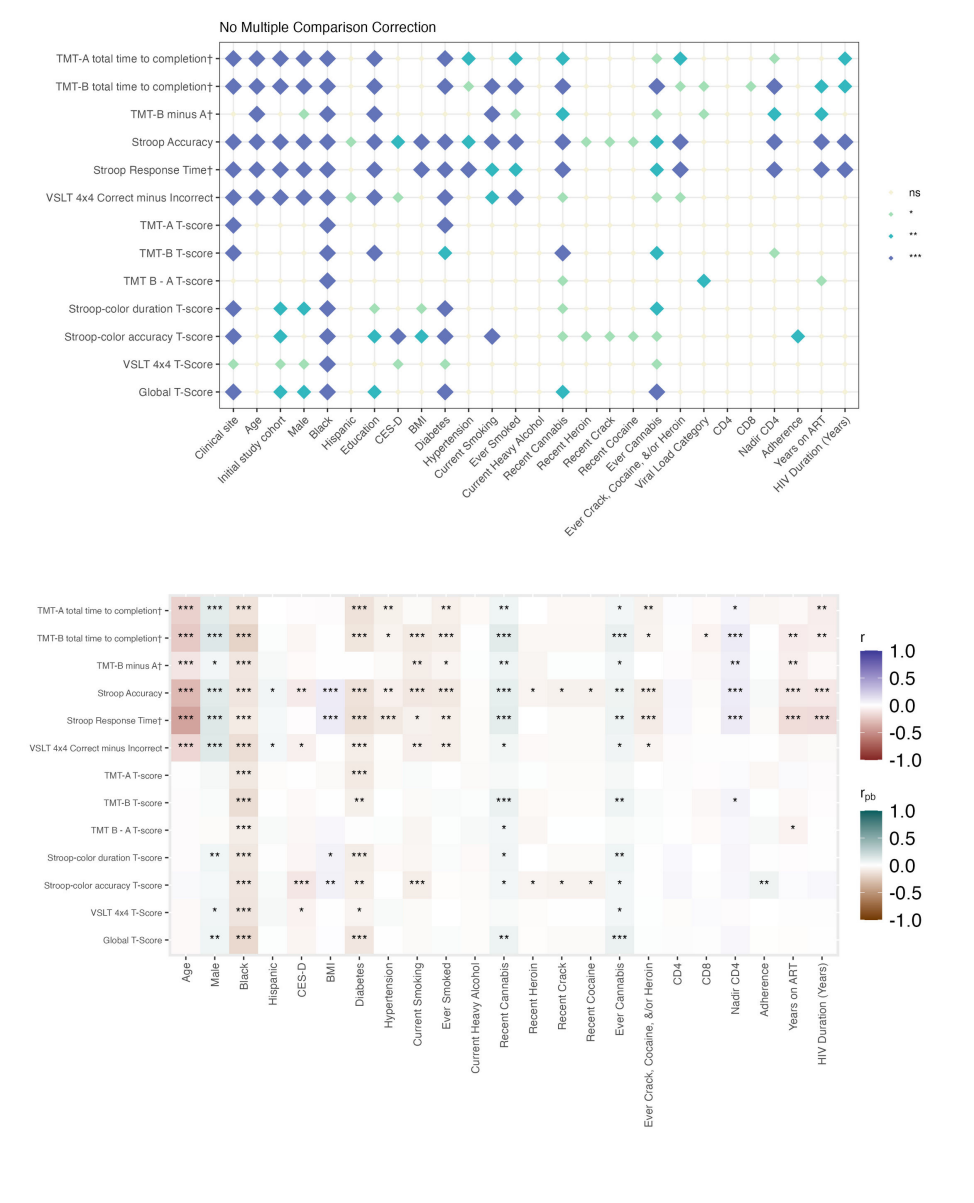
Cognitive correlates among people with HIV. The top panel displays associations between the examined variables and cognitive performance (raw scores and T-scores). † Denotes raw scores that have been reverse-scored so that higher scores indicate better performance. The bottom panel illustrates the direction and strength of associations. Pearson correlations were used for continuous variables (eg, age) and point-biserial correlations for binary categorical variables. Significance thresholds are denoted as ***P<.001; **P<.01; *P<.05; biological sex=male; ns: not significant. TMT: Trail Making Test; VSLT: Visual Spatial Learning Test.

In both people with HIV and people without HIV, a greater number of factors were associated with raw BRACE scores than with demographically adjusted T-scores. Associations for continuous variables and 2-level categorical variables are shown in Figures1  2, whereas the results for categorical variables with more than 2 levels are presented in Tables S5-S11 in Multimedia Appendix 2. Across serostatus groups, clinical research site, study cohort, and sociodemographic factors (age, race/ethnicity, and education) were significantly related to raw test scores across most outcome measures. Older age, being Black or African American, and having fewer years of education were associated with poorer performance. These associations for race and sex were attenuated when using age- and education-adjusted T-scores. Behavioral and clinical factors, including diabetes, hypertension, depressive symptoms, and substance use (except cannabis), were also consistently associated with poorer raw scores in both groups, although these relationships were often weakened when using T-scores. Among people with HIV, HIV-related factors, such as nadir cluster of differentiation 4 (CD4) count, years of ART, and HIV duration, were primarily associated with raw scores rather than T-score performance.

When analyses were stratified by biological sex, most associations with BRACE performance were again observed for raw scores rather than T-scores (Figures3^6^**,** Tables S12-S27 in Multimedia Appendix 2). Across all 4 groups, age and education showed the most consistent associations with cognitive performance (Figure S1 and Tables S28-S30 in Multimedia Appendix 2).

Among women, associations between demographic factors and cognitive performance were generally more extensive and stronger in people with HIV (Figure 3, Tables S12-S16 in Multimedia Appendix 2) than in people without HIV (Figure 4, Tables S17-S20 in Multimedia Appendix 2). Menopausal status was significantly related to raw scores but not T-scores, in both women with and without HIV. In women with HIV, additional associations were observed with clinical research site and Black or African American race. Depressive symptoms (CES-D score) were associated with poorer performance on Stroop-Color accuracy in people with HIV and VSLT in people without HIV. Diabetes, hypertension, and smoking (current or ever) were associated with poorer raw scores in both groups of women, whereas other forms of substance use showed stronger associations in women without HIV.

Among men, cognitive performance was associated with a broader range of factors overall, with stronger associations observed among people with HIV (Figure 5, Tables S21-S24 in Multimedia Appendix 2) than among people without HIV (Figure 6, Tables S25-S27 in Multimedia Appendix 2). In men with HIV, substance use, particularly cannabis, was significantly related to both raw and T-scores across multiple cognitive tests. Diabetes was also more strongly associated with poorer cognition in men with HIV than in men without HIV. Additional results for the total sample and sex-stratified analyses are presented in Figures S3-S5 and Tables S28-S37 in Multimedia Appendix 2.

**Figure 2.**
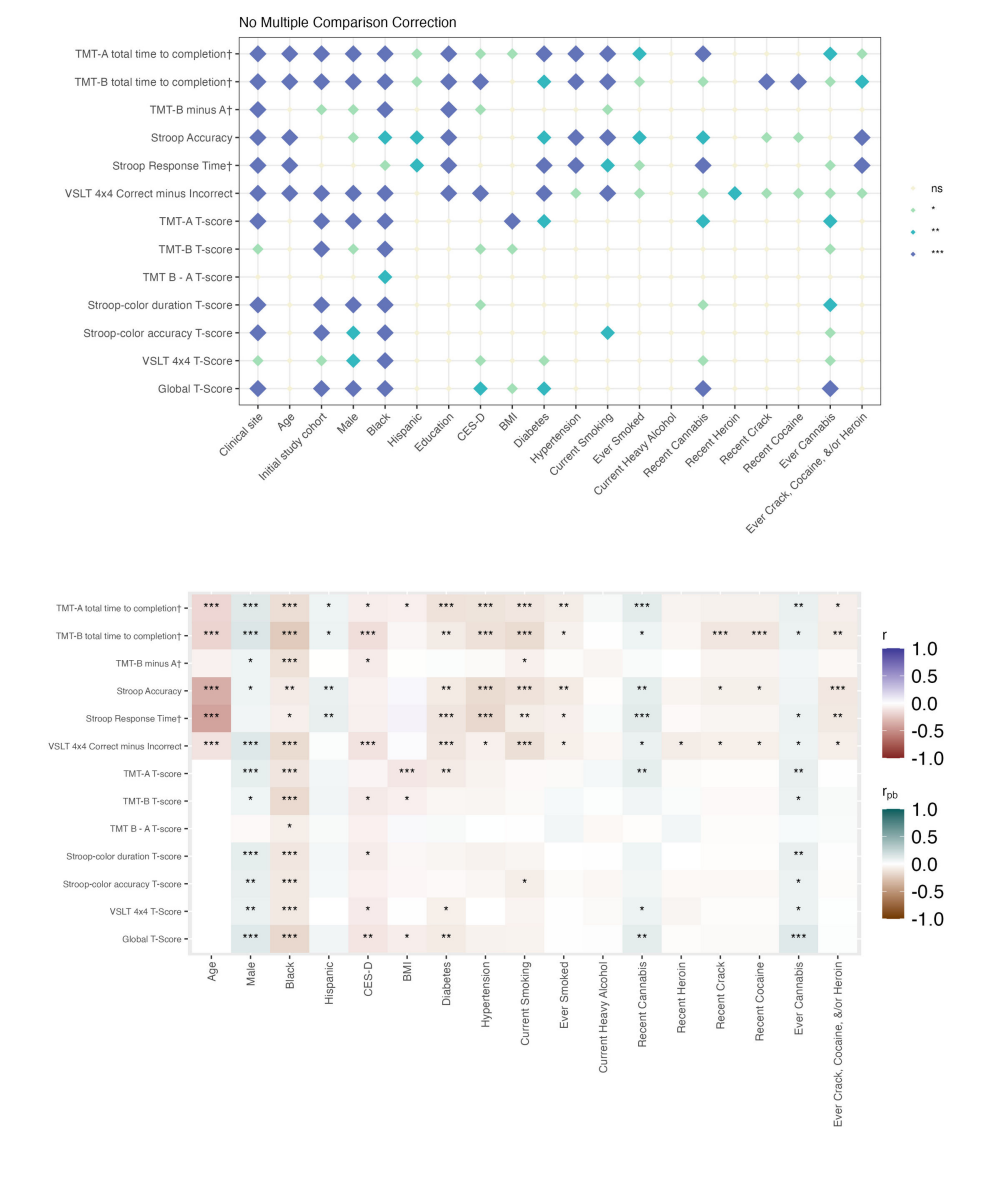
Cognitive correlates among people without HIV. The top panel displays associations between examined variables and cognitive performance (raw scores and T-scores). † Denotes raw scores that have been reverse-scored so that higher scores indicate better performance. The bottom panel illustrates the direction and strength of associations: Pearson correlations were used for continuous variables (eg, age), and point-biserial correlations for binary categorical variables. Significance thresholds are denoted as ***P<.001; **P<.01; *P<.05. Biological sex=male; ns: not significant. TMT: Trail Making Test; VSLT: Visual Spatial Learning Test.

**Figure 3.**
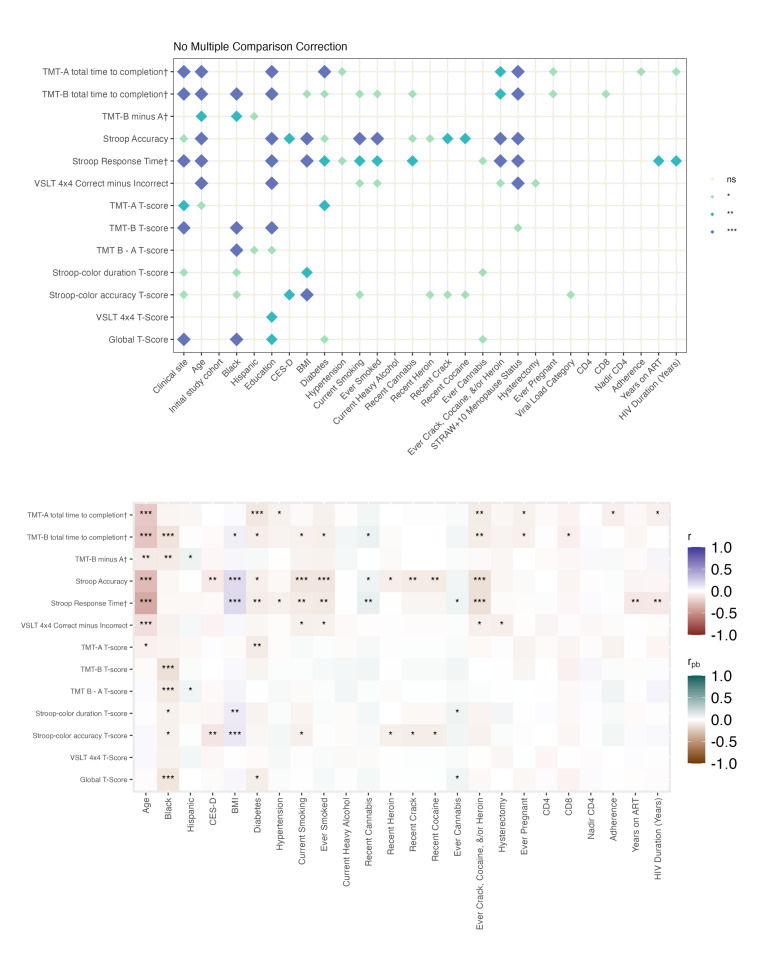
Cognitive correlates among women with HIV. The top panel displays associations between examined variables and cognitive performance (raw scores and T-scores). † Denotes raw scores that have been reverse-scored so that higher scores indicate better performance. The bottom panel illustrates the direction of and strength of associations: Pearson correlations were used for continuous variables (eg, age), and point-biserial correlations for binary categorical variables. Significance thresholds are denoted as ***P<.001; **P<.01; *P<.05; ns: not significant. TMT: Trail Making Test; VSLT: Visual Spatial Learning Test.

**Figure 4.**
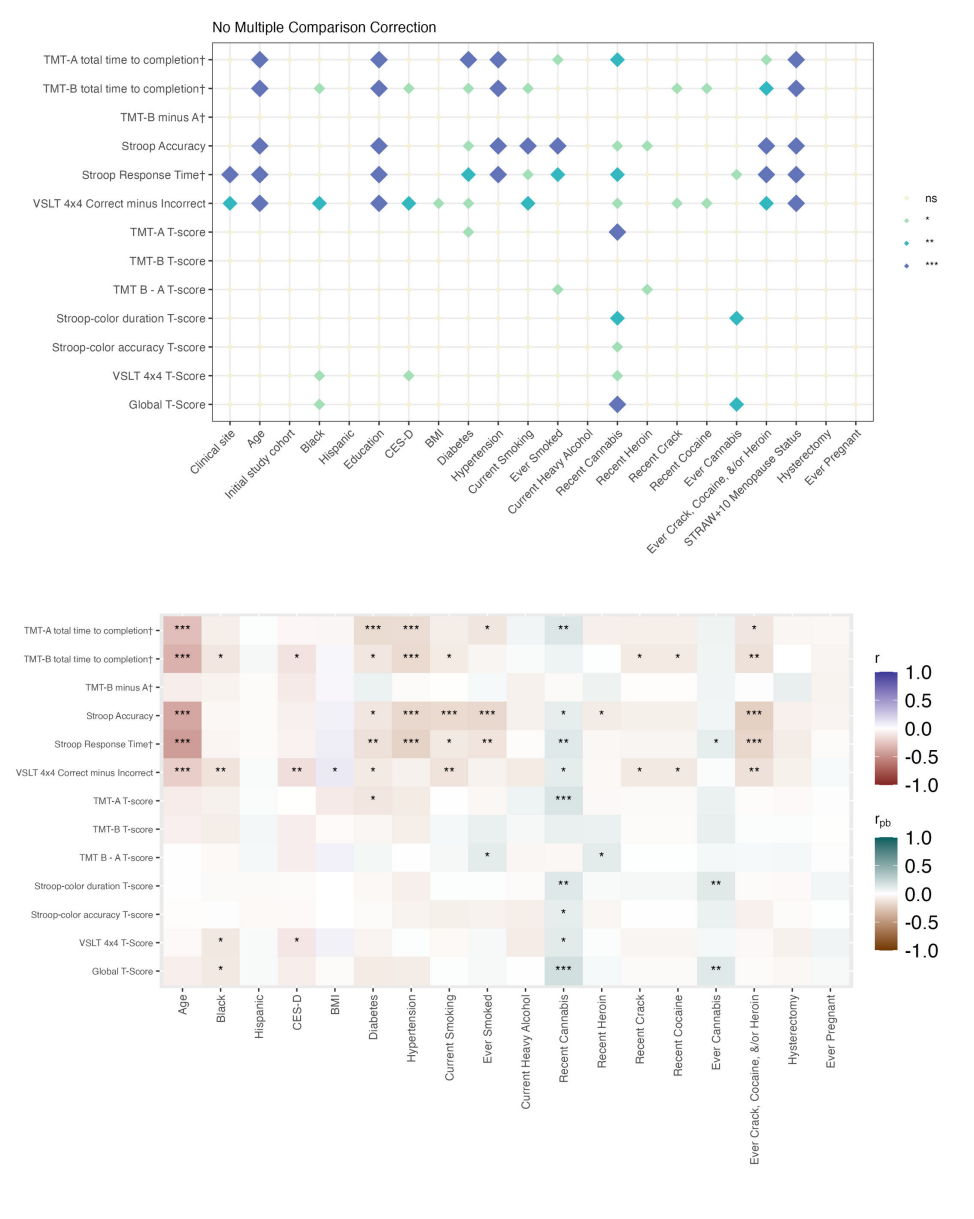
Cognitive correlates among women without HIV. The top panel displays associations between examined variables and cognitive performance (raw scores and T-scores). † Denotes raw scores that have been reverse-scored so that higher scores indicate better performance. The bottom panel illustrates the direction of and strength of associations: Pearson correlations were used for continuous variables (eg, age), and point-biserial correlations for binary categorical variables. Significance thresholds are denoted as ***P<.001; **P<.01; *P<.05; ns: not significant. TMT: Trail Making Test; VSLT: Visual Spatial Learning Test.

**Figure 5.**
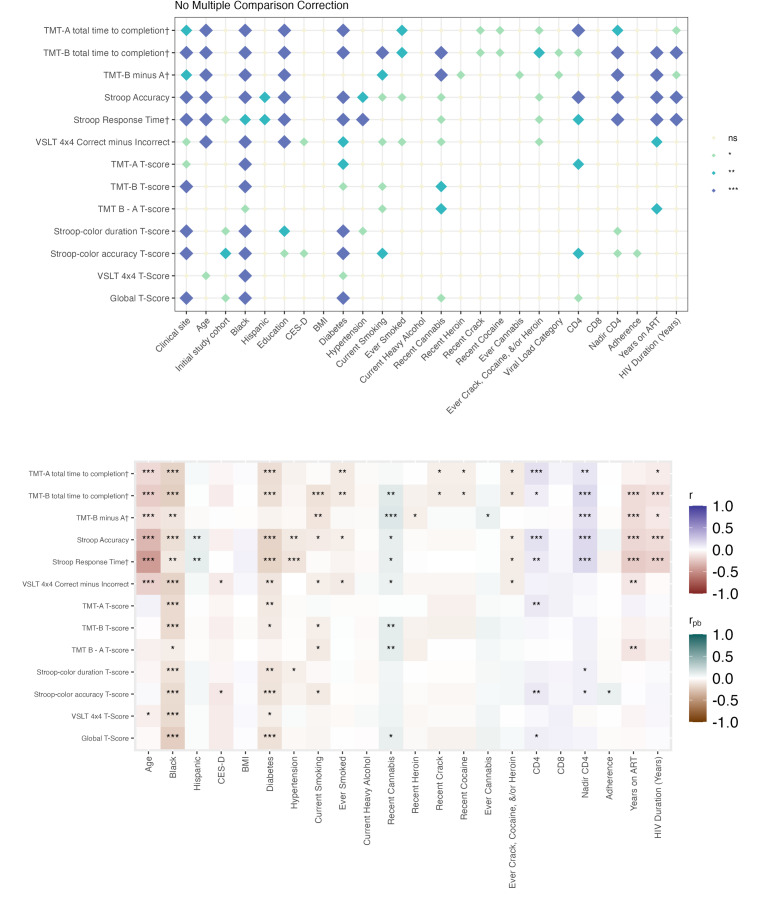
Cognitive correlates among men with HIV. The top panel displays associations between examined variables and cognitive performance (raw scores and T-scores). † Denotes raw scores that have been reverse-scored so that higher scores indicate better performance. The bottom panel illustrates the direction of and strength of associations: Pearson correlations were used for continuous variables (eg, age), and point-biserial correlations for binary categorical variables. Significance thresholds are denoted as ***P<.001; **P<.01; *P<.05; ns: not significant. TMT: Trail Making Test; VSLT: Visual Spatial Learning Test.

**Figure 6.**
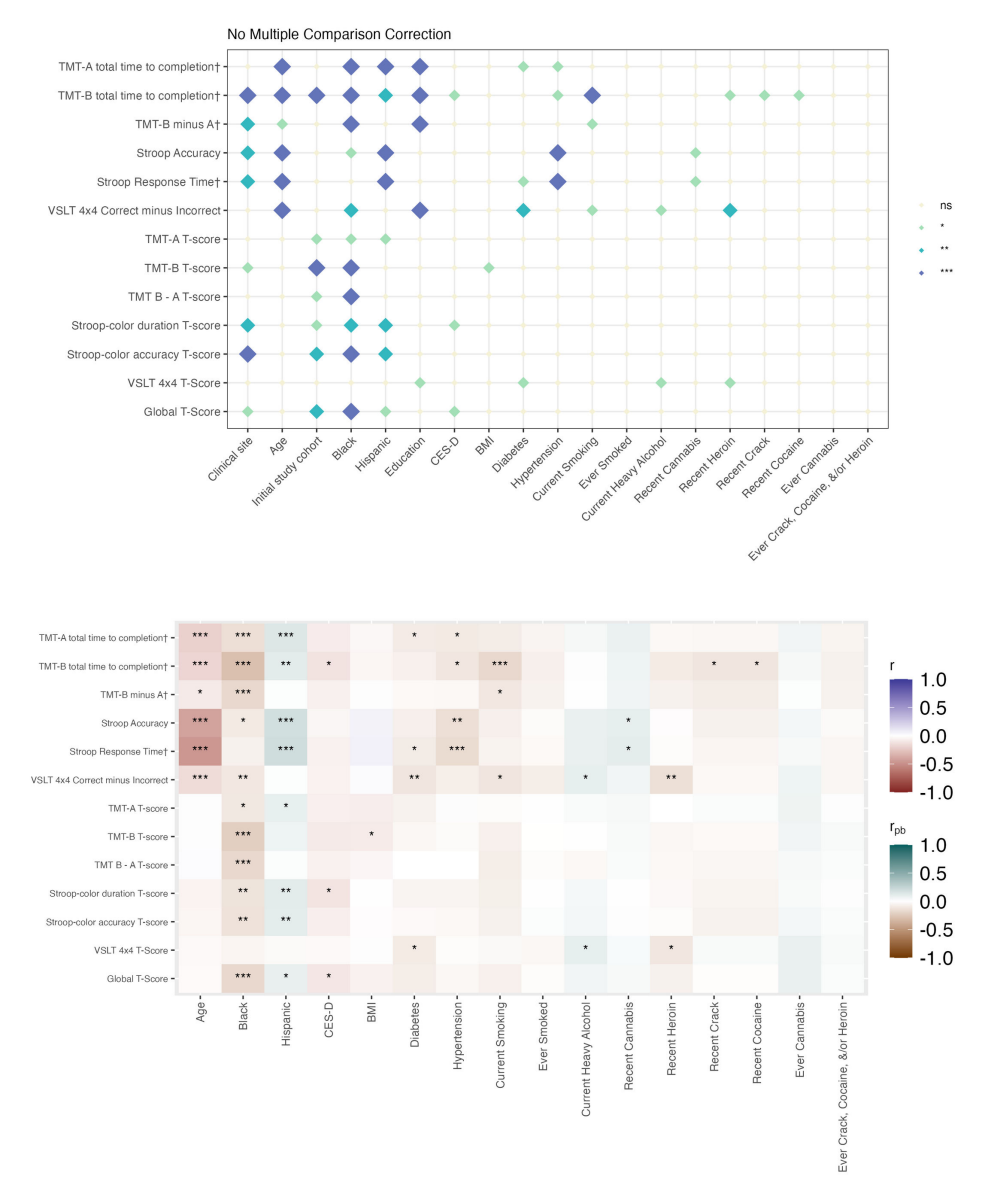
Cognitive correlates among men without HIV. The top panel displays associations between examined variables and cognitive performance (raw scores and T-scores). † Denotes raw scores that have been reverse-scored so that higher scores indicate better performance. The bottom panel illustrates the direction of and strength of associations: Pearson correlations were used for continuous variables (eg, age), and point-biserial correlations for binary categorical variables. Significance thresholds are denoted as ***P<.001; **P<.01; *P<.05; ns: not significant. TMT: Trail Making Test; VSLT: Visual Spatial Learning Test.

## Discussion

This study establishes the first regression-based normative framework for BRACE using data from a large and diverse sample of people with and without HIV. The analyses incorporated multiple demographic models (age only; age and education; age, education, and race/ethnicity; age, education, and biological sex; and age, education, race/ethnicity, and biological sex). Building on our prior work demonstrating that BRACE is easily self-administered, reliable, resistant to short-term practice effects, and strongly correlated with a standard neuropsychological battery [1], this study extends validation by examining HIV serostatus differences, within-sex comparisons, and demographic and clinical correlates of cognitive performance across 4 cognitive measures (TMT-A and B, Stroop-Color, and VSLT). These norms provide a framework for interpreting BRACE performance relative to age and education and support standardized evaluation across research and clinical settings.

Overall, cognitive differences between people with HIV and people without HIV were minimal, with very small effect sizes observed for Stroop-Color. Notably, these serostatus differences were predominantly observed in men. This finding contrasts with a systematic review suggesting that women with HIV are more vulnerable to cognitive impairment than men with HIV [2,11]. While many prior studies were underpowered to detect sex differences [2], one well-powered MWCCS analysis using traditional pencil-and-paper neuropsychological tests found a female disadvantage. Women with HIV had 2.5 times higher odds of scoring in the impaired range on the TMT-A and on the nondominant hand of the Grooved Pegboard Test (not assessed in BRACE) [3]. However, that study included a younger cohort (mean age in the early 40s vs 50s-60s here), lower ART adherence, higher cluster of differentiation 4 counts, and higher viral loads and represented only about one-fourth of the MWCCS. These differences may reflect variations in cohort composition, aging, improved viral suppression, and methodological differences between tablet-based and traditional testing.

Beyond serostatus and biological sex, cognitive performance was related to a broad set of sociodemographic, behavioral, and clinical factors across both people with HIV and people without HIV. Consistent with prior work, older age, fewer years of education, and Black or African American race were associated with poorer performance, highlighting persistent disparities in cognitive health [12,13]. These associations likely reflect broader structural and contextual determinants of cognitive health rather than intrinsic group differences. Depressive symptoms and cardiometabolic comorbidities, particularly diabetes and hypertension, were also associated with worse cognition, reinforcing their role as modifiable risk factors for cognitive health in aging populations. Substance use patterns showed nuanced associations. Most substances were associated with poorer performance, whereas cannabis use was associated with better performance, consistent with some prior studies [14,15]. This finding should be interpreted cautiously, as it may reflect residual confounding or other unmeasured behavioral or clinical factors rather than a beneficial effect of cannabis itself. These findings suggest that BRACE is sensitive to broader determinants of cognitive health that cut across serostatus.

A major strength of this study is the large (N=2937), diverse normative sample (mean age 56, SD 12.34 years; 53% [n=1552] women; 51% [n=1508] Black/African American; and 12% [n=347] Hispanic/Latino) recruited across multiple US sites, enhancing generalizability. Generating regression-based norms across multiple demographic models provides flexibility for future BRACE applications. To minimize residual confounding due to demographic and clinical differences between men and women, we examined HIV serostatus differences *within* sex rather than sex differences *within* HIV serostatus. This approach improves internal validity but limits direct inferences about sex differences among people with HIV and people without HIV.

Several limitations should be considered. The cross-sectional design precludes causal inference, and BRACE assesses a relatively narrow range of cognitive abilities compared to comprehensive neuropsychological batteries. Although tablet-based cognitive assessments, such as BRACE, offer advantages for scalability and standardized administration, they may also introduce modality-specific challenges that are less common in traditional neuropsychological testing. In this study, administration difficulties were documented in routine coordinator notes, including touchscreen or stylus interaction issues (eg, among some participants with long fingernails or limited hand dexterity), intermittent software interruptions, and participant fatigue or drowsiness during testing. These observations should be interpreted cautiously, as they were not systematically measured; however, they highlight the importance of documenting testing context and user interaction when implementing digital cognitive assessments in research settings. Potentially relevant factors related to cognitive function, including sleep, HIV reservoir dynamics, and sex hormones, were not available in this analysis and warrant further investigation. As BRACE was only available in English, findings may not generalize to non-English-speaking populations. Additionally, since the normative equations were derived from people without HIV and then applied to both groups, observed HIV serostatus differences should be interpreted relative to the people without HIV reference group. Accordingly, the present regression-based norms should be viewed as an initial framework requiring further refinement and validation. Future work should evaluate their performance, including assessments of criterion and construct validity, potential adjustment for practice effects, and the incorporation of additional demographic or contextual factors. Although we intentionally did not use race-based corrections in the primary normative model, the current approach does not fully account for broader social and structural determinants that may shape cognitive performance. Future work should evaluate whether incorporating indicators of socioeconomic disadvantage, opportunity, and related contextual factors improves normative precision. Longitudinal analyses of BRACE data in the MWCCS will be essential to characterize cognitive trajectories in people with HIV and people without HIV and to identify early markers of cognitive decline or resilience.

In conclusion, we developed and applied regression-based normative equations for BRACE using data from people without HIV, providing an initial framework for the standardized evaluation of cognitive performance in people with HIV. Cognitive differences between groups were minimal overall and, when present, were largely driven by men with HIV. BRACE performance was also shaped by sociodemographic and clinical factors common to both groups, underscoring that cognitive difficulties likely reflect multiple intersecting influences. BRACE appears to be a promising tool for scalable cognitive assessment in large cohorts; however, additional external validation will be important before broader applicability can be established. Future cross-sectional and longitudinal studies are needed to further validate these norms and clarify sex- and serostatus-specific trajectories of cognitive performance.

## Supplementary material

10.2196/70207 Multimedia Appendix 1 Institutional review board names and approval numbers for each Multicenter AIDS Cohort Study and Women’s Interagency HIV Study site.

10.2196/70207 Multimedia Appendix 2 Tables and figures depicting regression coefficients, constants, and standard errors for regression models and T*-*scores, cognitive correlates, differences in cognition, cohort differences.

## Abbreviations

ART: antiretroviral therapy
BRACE: BrainBaseline Assessment of Cognition and Everyday Functioning
CES-D: Center for Epidemiologic Studies Depression Scale
MACS: Multicenter AIDS Cohort Study
MWCCS: Multicenter AIDS Cohort Study and Women’s Interagency HIV Study
QC: quality control
STRAW: Staging of Reproductive Aging Workshop in women
TMT A&B: Trail Making Test A&B
VSLT: Visual Spatial Learning Test
WIHS: Women’s Interagency HIV Study
