# Supplementary material for: BrainBaseline Assessment of Cognition and Everyday Functioning (“BRACE”-ing for the Future): Establishing iPad-Based Norms for Cognitive Function in the Multicenter AIDS Cohort Study and Women’s Interagency HIV Study Combined Cohort Study
Source: JMIR Ment Health. 2026 May 28;13:e70207. doi: 10.2196/70207 (PMC13218720; doi:10.2196/70207)
Supplement: Multimedia Appendix 1 [file mental-v13-e70207-s001.docx]

| **Appendix.** IRB names and approval numbers for each MWCCS site. | | |
| --- | --- | --- |
| **Subsite** | **IRB number** | **IRB of record** |
| Atlanta | STUDY00002773 | Emory University Institutional Review Board |
| JHU CRS | 16882 | Johns Hopkins Bloomberg School of Public Health IRB |
| DC Whitman Walker | Pro00055451 | Advarra |
| UMMC | 2020-1120 | University of Mississippi Medical Center IRB |
| UAB | IRB-130607002 | University of Alabama at Birmingham IRB |
| Bronx | BRANY File # 21-02-210-01E | BRANY |
| Brooklyn | 1544986-46 | SUNY Downstate IRB and Privacy Board |
| Chapel Hill | 21-0857 | University of North Carolina at Chapel Hill IRB |
| Chicago Cook County | BRANY File # 23-02-723-1540 | BRANY |
| Chicago Rush U | 19071813 | Rush Institutional Review Board |
| Chicago Core Center | 09-044 | Cook County Health |
| Chicago Northwestern | STU00022906 | Northwestern University IRB |
| DC Georgetown | 1993-077 | Georgetown University |
| DC Inova | INOVA-2024-142 | Inova IRB |
| Los Angeles | IRB-20-2292 | UCLA |
| Miami | 20210476 | University of Miami |
| Ohio | 2021H0423 | The Ohio State University |
| Pittsburgh | STUDY21050072 | University of Pittsburgh |
| San Francisco | 21-33925 | UCSF IRB |
| Los Angeles | HS-21-00496 | University of Southern California IRB |
| –JHU DACC | 23015 | Johns Hopkins Bloomberg School of Public Health IRB |
|  |  |  |
