# Supplementary material for: BrainBaseline Assessment of Cognition and Everyday Functioning (“BRACE”-ing for the Future): Establishing iPad-Based Norms for Cognitive Function in the Multicenter AIDS Cohort Study and Women’s Interagency HIV Study Combined Cohort Study
Source: JMIR Ment Health. 2026 May 28;13:e70207. doi: 10.2196/70207 (PMC13218720; doi:10.2196/70207)

**Supplemental Table 1.** Regression coefficients, constants, and standard errors for regression models and *T-*scores as a function of demographic variables (age) for the normative sample of people without HIV (n=1063).

|  |  |  |  |
| --- | --- | --- | --- |
| Variable | Intercept | Age | SE |
| TMT-A | -1.073*** | -0.003*** | 0.195 |
| TMT-B | -1.482*** | -0.002*** | 0.155 |
| TMT-B - A | -19.963*** | -0.086* | 14.156 |
| Stroop-color duration | 0.241*** | -0.003*** | 0.083 |
| Stroop-color accuracy | 35.687*** | -0.124*** | 3.972 |
| VSLT | 1.836*** | -0.027*** | 2.57 |

****P*<0.001; ***P*<0.01; **P*<0.05; SE=standard error; TMT-Trial Making Test; VSLT=Visual spatial learning Test

**Supplemental Table 2**. Regression coefficients, constants, and standard errors for regression models and *T-*scores as a function of demographic variables (age, education, biological sex) for the normative sample of people without HIV (n=1063).

|  |  |  | Education (referent =completed high school) | | | |  |  |
| --- | --- | --- | --- | --- | --- | --- | --- | --- |
| Variable | Intercept | Age | Less than high school | Some college | Completed/ Graduated with 4-year college degree | Attended/  completed graduate school | Male | SE |
| TMT-A | -1.064*** | -0.005*** | -0.022 | 0.065*** | 0.104*** | 0.108*** | 0.050*** | 0.186 |
| TMT-B | -1.473*** | -0.004*** | -0.011 | 0.075*** | 0.151*** | 0.150*** | 0.024* | 0.14 |
| TMT-B - A | -19.530*** | -0.163*** | -0.104 | 3.630** | 9.245*** | 8.978*** | -0.951 | 13.75 |
| Stroop-color duration | 0.248*** | -0.004*** | -0.009 | 0.026*** | 0.047*** | 0.048*** | 0.022*** | 0.079 |
| Stroop-color accuracy | 36.201*** | -0.162*** | -0.425 | 1.049** | 2.266*** | 2.462*** | 0.917*** | 3.732 |
| VSLT | 2.055*** | -0.049*** | -0.376 | 0.833*** | 1.157*** | 1.673*** | 0.537** | 2.443 |

****P*<0.001; ***P*<0.01; **P*<0.05; SE=standard error; TMT-Trial Making Test; VSLT=Visual spatial learning Test

**Supplemental Table 3**. Regression coefficients, constants, and standard errors for regression models and *T-*scores as a function of demographic variables (age, race/ethnicity, education) for the normative sample of people without HIV (n=1063).

|  |  |  |  | | Education (referent =completed high school) | | | |  |
| --- | --- | --- | --- | --- | --- | --- | --- | --- | --- |
| Variable | Intercept | Age | Black | Hispanic/  Latinx | Less than high school | Some college | Completed/ Graduated with 4-year college degree | Attended/  completed graduate school | SE |
| TMT-A | -1.008*** | -0.005*** | -0.055*** | -0.001 | -0.024 | 0.059*** | 0.104*** | 0.102*** | 0.186 |
| TMT-B | -1.406*** | -0.004*** | -0.063*** | -0.010 | -0.011 | 0.067*** | 0.137*** | 0.131*** | 0.138 |
| TMT-B - A | -16.533*** | -0.190*** | -2.826** | -0.275 | 0.002 | 3.290** | 7.684*** | 7.422*** | 13.712 |
| Stroop-color duration | 0.272*** | -0.004*** | -0.023*** | -0.001 | -0.009 | 0.023** | 0.048*** | 0.046*** | 0.079 |
| Stroop-color accuracy | 37.248*** | -0.162*** | -1.012*** | -0.078 | -0.453 | 0.934** | 2.287*** | 2.350*** | 3.731 |
| VSLT | 2.963*** | -0.052*** | -0.762*** | -0.433 | -0.364 | 0.749*** | 1.104*** | 1.543*** | 2.436 |

****P*<0.001; ***P*<0.01; **P*<0.05; SE=standard error; TMT-Trial Making Test; VSLT=Visual spatial learning Test

**Supplemental Table 4**. Regression coefficients, constants, and standard errors for regression models and *T-*scores as a function of demographic variables (age, race/ethnicity, education, biological sex) for the normative sample of people without HIV (n=1063).

|  |  |  | |  |  | | Education (referent =completed high school) | | | | | | |  | |  |
| --- | --- | --- | --- | --- | --- | --- | --- | --- | --- | --- | --- | --- | --- | --- | --- | --- |
| Variable | Intercept | | Age | | | Black | | Hispanic/  Latinx | Less than high school | Some college | Completed/ Graduated with 4-year college degree | Attended/  completed graduate school | Male | | SE | |
| TMT-A | -1.019*** | | -0.005*** | | | -0.045** | | 0.004 | -0.021 | 0.060*** | 0.091*** | 0.093*** | 0.039** | | 0.185 | |
| TMT-B | -1.409*** | | -0.004*** | | | -0.061*** | | -0.008 | -0.010 | 0.067*** | 0.134*** | 0.129*** | 0.010 | | 0.138 | |
| TMT-B - A | -16.051*** | | -0.181*** | | | -3.295** | | -0.480 | -0.113 | 3.245** | 8.293*** | 7.831*** | -1.731 | | 13.699 | |
| Stroop-color duration | 0.267*** | | -0.004*** | | | -0.018** | | 0.001 | -0.008 | 0.024** | 0.042*** | 0.041*** | 0.018** | | 0.079 | |
| Stroop-color accuracy | 37.048*** | | -0.166*** | | | -0.818** | | 0.007 | -0.405 | 0.952** | 2.035*** | 2.180*** | 0.717** | | 3.72 | |
| VSLT | 2.856*** | | -0.054*** | | | -0.658*** | | -0.388 | -0.338 | 0.759*** | 0.969*** | 1.453*** | 0.383* | | 2.432 | |

****P*<0.001; ***P*<0.01; **P*<0.05; SE=standard error; TMT-Trial Making Test; VSLT=Visual spatial learning Test

**Supplemental Table 5**. Clinical site differences in cognition among people with HIV.

|  | **Bronx**  N = 131^1^ | **Brooklyn**  N = 201^1^ | **DC**  N = 128^1^ | **SF**  N = 137^1^ | **Chicago-CC** N = 131^1^ | **Chapel Hill**  N = 191^1^ | **Atlanta**  N = 206^1^ | **Miami**  N = 133^1^ | **BHAM**  N = 90^1^ | **Jackson**  N = 88^1^ | **Balti**  N = 136^1^ | **Chicago-NW**  N = 128^1^ | **Pitt/Ohio**  N = 58^1^ | **LA**  N = 116^1^ | ***P*-value** | η² |
| --- | --- | --- | --- | --- | --- | --- | --- | --- | --- | --- | --- | --- | --- | --- | --- | --- |
| TMT-A total time to completion(ms) | 23,149 (14,599) | 23,129 (13,490) | 19,030 (10,795) | 17,801 (9,493) | 18,516 (8,818) | 17,277  (9,053) | 20,720 (11,784) | 21,192 (13,178) | 18,534 (10,324) | 19,453 (11,484) | 18,066 (8,559) | 17,568 (7,142) | 15,226  (5,650) | 18,480 (9,618) | **<.001** | .04 |
| TMT-B total time to completion(ms) | 51,010 (13,872) | 48,757  (14,151) | 45,693 (14,290) | 41,557 (14,931) | 47,222 (14,833) | 42,361 (15,620) | 48,361 (14,562) | 45,606.67 (15,351) | 43,695 (15,593) | 45,430 (14,865) | 43,758 (14,460) | 42,279 (14,287) | 38,333 (14,557) | 45,188 (14,704) | **<.001** | .04 |
| TMT-B minus A(ms) | 27,861 (15,499) | 25,844 (15,070) | 26,663 (14,647. | 23,755 (13,260) | 28,705 (15,153) | 25,084  (14,839) | 27,641 (15,802) | 24,415 (13,831) | 25,161 (15,846) | 25,976 (12,865) | 25,691 (13,094) | 24,793 (12,541) | 23,106 (13,084) | 26,708 (13,202) | .113 | .01 |
| Stroop Accuracy | 26.94 (4.48) | 27.45 (4.12) | 28.46 (3.83) | 28.24 (4.49) | 27.05 (4.10) | 29.40 (4.09) | 28.14 (4.27) | 27.47 (5.06) | 28.09 (4.67) | 28.11 (5.50) | 29.07 (4.43) | 27.90 (4.28) | 30.23  (3.02) | 28.58 (4.37) | **<.001** | .03 |
| Stroop Response Time (ms) | 927.23 (206.52) | 920.32 (181.83) | 891.11 (161.98) | 894.02 (184.16) | 940.92 (188.00) | 831.94 (177.47) | 885.32 (195.91) | 890.19 (227.97) | 828.16 (159.86) | 850.85 (184.89) | 855.09 (192.33) | 925.33 (206.34) | 814.71 (135.44) | 871.85 (191.36) | **<.001** | .04 |
| VSLT 4x4 Correct minus Incorrect | -0.34 (2.13) | -0.37 (2.23) | -0.12 (2.52) | 0.45 (2.51) | -0.16 (2.28) | 0.38 (2.73) | -0.17 (2.36) | 0.13  (2.87) | -0.02 (2.66) | 0.27 (2.66) | 0.35 (2.63) | 0.38 (2.54) | 1.48  (2.82) | 0.64 (2.51) | **<.001** | .02 |
| TMT-A T-score | 48.66 (11.25) | 47.76 (11.18) | 51.54 (9.57) | 52.44 (9.25) | 50.98 (8.99) | 51.02 (8.56) | 48.87 (9.88) | 49.09 (10.67) | 49.96 (9.58) | 48.91 (10.42) | 50.84 (8.30) | 50.96 (7.53) | 53.36 (7.29) | 50.59 (8.47) | **<.001** | .02 |
| TMT-B T-score | 47.82 (8.90) | 48.52 (9.26) | 50.69 (10.18) | 53.48 (10.78) | 48.80 (10.00) | 50.59 (10.38) | 47.95 (9.37) | 49.87 (9.63) | 50.33 (12.94) | 47.71 (11.27) | 50.18 (10.08) | 50.53 (9.75) | 52.84 (10.60) | 49.36 (9.55) | **<.001** | .02 |
| TMT B - A T-score | 49.22 (11.13) | 50.32 (10.78) | 49.64 (10.57) | 51.49 (9.16) | 47.85 (10.63) | 49.50 (10.00) | 48.70 (11.03) | 50.73 (9.60) | 49.91 (11.65) | 48.87 (9.70) | 49.27 (9.14) | 49.40 (8.61) | 49.96 (8.84) | 48.81 (9.13) | .284 | .01 |
| Stroop-color duration T-score | 49.00 (10.55) | 48.25 (9.42) | 50.52 (8.90) | 50.06 (10.06) | 45.94 (9.59) | 50.14 (10.29) | 48.86 (10.33) | 49.62 (11.24) | 49.22 (10.63) | 48.12 (10.80) | 52.50 (11.42) | 48.00 (9.42) | 53.74 (7.41) | 49.86 (10.53) | **<.001** | .03 |
| Stroop-color accuracy T-score | 48.68 (10.95) | 47.99 (10.31) | 51.25 (9.13) | 50.44 (10.88) | 45.86 (10.30) | 50.51 (9.89) | 48.19 (11.66) | 49.99 (11.05) | 47.19 (12.63) | 46.43 (14.67) | 51.85 (11.73) | 48.35 (9.19) | 54.02 (6.64) | 49.88 (10.44) | **<.001** | .03 |
| VSLT 4x4 T-Score | 49.11 (8.08) | 48.26 (9.15) | 49.13 (9.15) | 51.29 (10.07) | 48.53 (8.85) | 49.38 (10.28) | 48.56 (9.75) | 49.71 (10.85) | 48.36 (11.04) | 48.87 (10.82) | 50.09 (10.34) | 49.19 (10.00) | 53.33 (11.49) | 51.18 (9.93) | **.016** | .01 |
| Global T-Score | 48.65 (6.50) | 48.20 (6.45) | 50.47 (6.78) | 51.82 (7.10) | 48.56 (5.66) | 50.28 (6.88) | 48.56 (6.09) | 49.57 (7.77) | 49.47 (7.40) | 48.40 (7.89) | 50.90 (6.97) | 49.67 (6.53) | 53.32 (6.35) | 50.25 (7.23) | **<.001** | .03 |

^1^Mean (SD); ANOVA used to examine clinical site differences in performance; Effect size method= η²

LA=Los Angeles; DC=Washington DC; SF=San Francisco; NW=Northwestern; CC=Cook County; BHAM=Birmingham; Balti=Baltimore; Pitt=Pittsburgh

**Supplemental Table 6**. Cohort differences in cognition among people with HIV.

|  | **MACS**  N = 410^1^ | **WIHS**  N = 826^1^ | **MWCCS**  N = 509^1^ | ***P*-value** | **η²** |
| --- | --- | --- | --- | --- | --- |
| TMT-A total time to completion(ms) | 17,708 (8,273) | 20,767 (12,420) | 18,666 (10,120) | **<.001** | .01 |
| TMT-B total time to completion(ms) | 42,876 (14,528) | 46,955 (14,645) | 44,890(15,558) | **<.001** | .01 |
| TMT-B minus A(ms) | 25,194 (12,904) | 26,202 (15,379) | 26,286(14,202) | .443 | .00 |
| Stroop Accuracy | 28.76 (4.23) | 27.90 (4.19) | 28.64 (4.74) | **<.001** | .01 |
| Stroop Response Time (ms) | 877.39 (191.81) | 904.38 (181.55) | 869.10 (202.92) | **.002** | .01 |
| VSLT 4x4 Correct minus Incorrect | 0.56 (2.62) | -0.16 (2.44) | 0.24 (2.52) | **<.001** | .01 |
| TMT-A T-score | 51.00 (8.25) | 49.60 (10.69) | 50.03 (9.34) | .061 | .00 |
| TMT-B T-score | 50.33 (10.02) | 49.34 (9.89) | 49.30 (10.43) | .209 | .00 |
| TMT B - A T-score | 49.37 (9.05) | 49.82 (11.16) | 48.98 (9.91) | .339 | .00 |
| Stroop-color duration T-score | 50.30 (10.39) | 48.72 (9.68) | 48.35 (10.63) | **.009** | .01 |
| Stroop-color accuracy T-score | 50.41 (10.36) | 48.64 (10.28) | 48.04 (11.72) | **.003** | .01 |
| VSLT 4x4 T-Score | 50.62 (10.31) | 48.85 (9.71) | 49.50 (9.59) | **.011** | .01 |
| Global T-Score | 50.56 (7.01) | 49.13 (6.71) | 49.30 (6.95) | **.002** | .01 |

^1^Mean (SD); ANOVA used to examine cohort differences in performance; Effect size method= η²

**Supplemental Table 7**. Differences in cognition by education level among people with HIV.

|  | **Less than high school**  N = 318^1^ | **Completed high school**  N = 428^1^ | **Some College**  N = 635^1^ | **Graduated College**  N = 180^1^ | **>College**  N = 306^1^ | ***P*-value** | **η²** |
| --- | --- | --- | --- | --- | --- | --- | --- |
| TMT-A total time to completion(ms) | 23,870 (14,105) | 20,705 (11,342) | 18,871 (10,631) | 15,967 (7,139) | 16,428 (7,127) | **<.001** | .05 |
| TMT-B total time to completion(ms) | 53,090 (13,819) | 48,955 (14,050) | 44,006 (14,647) | 38,461 (13,974) | 39,079 (13,552) | **<.001** | .11 |
| TMT-B minus A(ms) | 29,220 (15,054) | 28,377 (14,441) | 25,134 (14,898) | 22,494 (12,030) | 22,651 (12,610) | **<.001** | .03 |
| Stroop Accuracy | 26.65 (4.87) | 27.75 (4.25) | 28.91 (4.05) | 29.71 (4.70) | 29.20 (4.10) | **<.001** | .05 |
| Stroop Response Time (ms) | 951.32 (215.04) | 911.28 (192.11) | 859.44 (173.62) | 831.73 (188.88) | 861.35 (179.39) | **<.001** | .04 |
| VSLT 4x4 Correct minus Incorrect | -0.67 (2.08) | -0.36 (2.41) | 0.27 (2.57) | 0.75 (2.79) | 0.98 (2.54) | **<.001** | .05 |
| TMT-A T-score | 49.86 (11.45) | 50.93 (10.23) | 49.71 (9.94) | 49.73 (8.03) | 49.49 (7.96) | .250 | .00 |
| TMT-B T-score | 49.52 (9.32) | 51.03 (9.48) | 49.54 (10.42) | 48.02 (10.76) | 48.25 (10.14) | **<.001** | .01 |
| TMT B - A T-score | 49.61 (11.02) | 50.08 (10.35) | 49.88 (10.74) | 48.21 (8.69) | 48.47 (8.94) | .083 | .00 |
| Stroop-color duration T-score | 49.12 (11.33) | 49.54 (10.33) | 49.69 (9.44) | 48.43 (10.31) | 47.53 (9.76) | **.025** | .01 |
| Stroop-color accuracy T-score | 48.20 (12.72) | 49.36 (10.82) | 49.94 (10.07) | 48.29 (11.89) | 47.54 (9.53) | **.010** | .01 |
| VSLT 4x4 T-Score | 50.51 (8.43) | 49.87 (9.55) | 49.15 (10.21) | 49.05 (11.07) | 48.52 (10.22) | .090 | .00 |
| Global T-Score | 49.75 (7.16) | 50.34 (6.88) | 49.52 (6.73) | 48.81 (7.31) | 48.44 (6.45) | **.003** | .01 |

^1^Mean (SD); ANOVA used to examine education differences in performance Effect size method= η²

**Supplemental Table 8**. Differences in cognition by viral load status among people with HIV.

| **Characteristic** | **<=20**  N = 931^1^ | **21-200**  N = 557^1^ | **201-500**  N = 24^1^ | **501-1000**  N = 11^1^ | **>1000**  N = 79^1^ | ***P*-value** | η² |
| --- | --- | --- | --- | --- | --- | --- | --- |
| TMT-A total time to completion(ms) | 19,489 (11,150) | 19,602 (10,896) | 17,983 (11,194) | 20,796 (9,823) | 16,607 (7,055) | .198 | .00 |
| TMT-B total time to completion(ms) | 44,250 (14,916) | 46,535 (15,194) | 43,693 (14,223) | 52,280 (16,162) | 46,905 (14,551) | **.019** | .01 |
| TMT-B minus A(ms) | 24,820 (14,571) | 26,932 (14,405) | 25,710 (13,509) | 31,484 (14,094) | 30,298 (13,779) | **.002** | .01 |
| Stroop Accuracy | 28.46 (4.32) | 28.20 (4.51) | 27.50 (4.43) | 27.27 (5.39) | 27.76 (3.99) | .387 | .00 |
| Stroop Response Time (ms) | 882.43 (181.34) | 893.28 (208.48) | 912.74 (161.84) | 907.94 (219.70) | 895.47 (156.70) | .761 | .00 |
| VSLT 4x4 Correct minus Incorrect | 0.20 (2.56) | 0.03 (2.49) | 0.25 (2.11) | -0.18 (1.72) | 0.22 (2.19) | .751 | .00 |
| TMT-A T-score | 49.95 (9.85) | 49.91 (9.71) | 51.08 (9.73) | 49.74 (9.34) | 52.28 (8.06) | .326 | .00 |
| TMT-B T-score | 50.22 (9.92) | 49.01 (10.17) | 49.22 (10.28) | 47.67 (8.48) | 48.08 (10.88) | .107 | .00 |
| TMT B - A T-score | 50.22 (10.32) | 48.91 (10.24) | 49.02 (10.20) | 46.87 (9.35) | 46.33 (10.24) | **.006** | .01 |
| Stroop-color duration T-score | 49.19 (9.74) | 48.95 (10.87) | 46.43 (8.97) | 48.43 (9.36) | 46.71 (9.42) | .207 | .00 |
| Stroop-color accuracy T-score | 49.21 (10.57) | 48.70 (11.06) | 46.01 (11.01) | 46.73 (12.89) | 46.27 (11.38) | .104 | .00 |
| VSLT 4x4 T-Score | 49.64 (10.03) | 49.17 (9.62) | 49.21 (8.76) | 50.21 (5.04) | 49.65 (8.79) | .925 | .00 |
| Global T-Score | 49.75 (6.80) | 49.26 (7.03) | 48.99 (6.41) | 49.01 (6.07) | 49.18 (6.52) | .695 | .00 |

^1^Mean (SD); ANOVA used to examine viral load differences in performance; Effect size method= η²

**Supplemental Table 9**. Clinical site differences in cognition among people without HIV.

|  | **Bronx**  N = 94^1^ | **Brooklyn**  N = 78^1^ | **DC**  N = 53^1^ | **SF**  N = 92^1^ | **Chicago-CC** N = 48^1^ | **Chapel Hill**  N = 54^1^ | **Atlanta**  N = 91^1^ | **Miami**  N = 67^1^ | **BHAM**  N = 36^1^ | **Jackson**  N = 43^1^ | **Balti** N = 117^1^ | **Chicago-NW**  N = 85^1^ | **Pitt/Ohio**  N = 93^1^ | **LA**  N = 112^1^ | ***P*-value** | η² |
| --- | --- | --- | --- | --- | --- | --- | --- | --- | --- | --- | --- | --- | --- | --- | --- | --- |
| TMT-A total time to completion(ms) | 20,685 (12,381) | 23,710 (14,1562) | 21,354 (13,442) | 19,628 (10,899) | 18,582 (10,398) | 19,046 (9,3434) | 19,194 (11,544) | 23,841 (15,626) | 19,493 (13,485) | 22,439 (13,464) | 17,089 (6,800) | 17,923 (8,510) | 16,937 (8,017) | 17,482 (8,871) | **<.001** | .04 |
| TMT-B total time to completion(ms) | 47,652  (15,074) | 50,505 (14,005) | 45,601 (15,255) | 43,185 (14,394) | 49,064 (15,390) | 47,561 (15,371) | 46,312 (15,076) | 49,970 (14,990) | 39,281 (15,666) | 45,632 (16,667) | 40,581 (14,402) | 41,812 (15,030) | 38,742 (13,195) | 40,611 (13,832) | **<.001** | .07 |
| TMT-B minus A(ms) | 26,966 (14,476) | 26,796 (16,692) | 24,247  (16,381) | 23,558 (13,745) | 30,482 (14,868) | 28,514 (14,749) | 27,118 (14,676) | 26,129 (15,417) | 19,788 (15,537) | 23,193 (13,756) | 23,492 (12,471) | 23,889 (12,964) | 21,805 (11,241) | 23,128  (12,839) | **.004** | .03 |
| Stroop Accuracy | 28.65 (5.01) | 27.97 (3.77) | 29.70 (4.15) | 28.88 (4.15) | 27.54 (2.87) | 28.46 (4.90) | 27.73 (4.33) | 27.54 (4.18) | 31.07 (4.77) | 29.00 (5.15) | 29.08 (4.05) | 28.48 (4.16) | 28.69 (3.74) | 29.59 (3.79) | **.022** | .03 |
| Stroop Response Time (ms) | 899.03 (246.67) | 920.71 (200.62) | 836.97 (149.29) | 876.34 (179.06) | 937.49 (159.82) | 860.12 (212.23) | 892.87 (174.09) | 944.96 (194.24) | 760.85 (184.11) | 839.05 (180.32) | 860.11 (178.12) | 895.53 (208.05) | 878.80 (197.23) | 853.51 (164.02) | **<.001** | .04 |
| VSLT 4x4 Correct minus Incorrect | -0.50 (2.18) | -0.50 (2.42) | 0.40 (2.63) | 0.51 (2.62) | -0.98 (2.44) | 0.00 (2.75) | 0.54 (2.66) | -0.36 (2.48) | 0.58 (2.64) | 0.51 (3.07) | 0.43 (2.58) | 0.91 (2.32) | 1.23 (2.56) | 0.75 (2.48) | **<.001** | .06 |
| TMT-A T-score | 50.82 (10.51) | 47.35 (10.58) | 48.37 (11.12) | 49.99 (10.10) | 52.03 (9.01) | 50.21 (8.62) | 50.98 (9.89) | 47.85 (12.18) | 48.51 (11.64) | 45.27 (10.56) | 51.73 (7.83) | 50.47 (8.37) | 51.75 (8.04) | 51.87 (8.89) | **.002** | .03 |
| TMT-B T-score | 50.52 (9.85) | 47.78 (8.25) | 49.05 (10.33) | 51.16 (10.08) | 48.78 (9.85) | 48.96 (10.02) | 49.70 (9.82) | 48.49 (9.81) | 51.37 (13.71) | 46.89 (10.59) | 51.80 (10.69) | 49.90 (10.19) | 52.14 (9.65) | 51.80 (9.16) | **.043** | .02 |
| TMT B - A T-score | 49.95 (10.09) | 50.00 (11.87) | 50.44 (11.30) | 51.21 (9.72) | 47.12 (10.45) | 48.30 (10.14) | 49.16 (10.32) | 50.47 (10.95) | 52.34 (11.65) | 50.28 (9.76) | 50.07 (8.84) | 49.13 (8.84) | 50.82 (7.95) | 50.42 (8.56) | .504 | .01 |
| Stroop-color duration T-score | 51.10 (11.69) | 48.12 (9.40) | 52.15 (7.81) | 50.13 (9.75) | 47.25 (9.22) | 50.21 (11.65) | 48.29 (9.80) | 46.86 (10.14) | 52.43 (9.73) | 47.35 (8.53) | 53.26 (10.39) | 50.06 (10.26) | 50.80 (10.11) | 53.09 (8.41) | **<.001** | .04 |
| Stroop-color accuracy T-score | 51.23 (11.20) | 49.32 (8.54) | 52.22 (9.77) | 50.59 (9.69) | 48.69 (7.61) | 48.56 (12.82) | 47.59 (10.21) | 47.15 (10.62) | 51.83 (9.90) | 46.28 (11.92) | 52.66 (10.11) | 50.23 (9.29) | 50.80 (9.37) | 52.78 (7.91) | **<.001** | .04 |
| VSLT 4x4 T-Score | 48.57 (8.96) | 48.08 (9.79) | 49.94 (9.97) | 51.08 (10.03) | 45.98 (9.21) | 49.48 (10.84) | 51.62 (10.32) | 49.06 (9.58) | 49.04 (11.08) | 49.34 (11.99) | 49.68 (10.30) | 50.71 (8.82) | 52.28 (9.65) | 51.03 (10.06) | **.021** | .03 |
| Global T-Score | 50.25 (6.71) | 47.83 (6.10) | 49.88 (6.30) | 50.59 (7.17) | 48.51 (5.79) | 49.71 (6.73) | 50.15 (6.54) | 48.06 (7.77) | 50.34 (8.38) | 47.21 (7.50) | 51.62 (6.98) | 50.28 (6.91) | 51.75 (6.68) | 51.95 (6.03) | **<.001** | .04 |

^1^Mean (SD); ANOVA used to examine clinical site differences in performance; Effect size method= η²

LA=Los Angeles; DC=Washington DC; SF=San Francisco; NW=Northwestern; CC=Cook County; BHAM=Birmingham; Balti=Baltimore; Pitt=Pittsburgh

**Supplemental Table 10**. Cohort differences in cognition among people without HIV.

|  | **MACS**  N = 398^1^ | **WIHS**  N = 359^1^ | **MWCCS**  N = 246^1^ | ***P*-value** | **η²** |
| --- | --- | --- | --- | --- | --- |
| TMT-A total time to completion(ms) | 17,463 (8,190) | 20,812 (12,444) | 20,630 (12,385) | **<.001** | .02 |
| TMT-B total time to completion(ms) | 40,573 (13,992) | 46,169 (15,136) | 47,109 (15,548) | **<.001** | .04 |
| TMT-B minus A(ms) | 23,109 (12,386) | 25,357 (15,293) | 26,479 (14,851) | **.008** | .01 |
| Stroop Accuracy | 28.93 (3.84) | 28.32 (4.30) | 28.69 (4.61) | .137 | .00 |
| Stroop Response Time (ms) | 874.33 (186.32) | 890.59 (194.23) | 868.35 (198.61) | .318 | .00 |
| VSLT 4x4 Correct minus Incorrect | 0.78 (2.47) | 0.03 (2.63) | 0.07 (2.64) | **<.001** | .02 |
| TMT-A T-score | 51.42 (8.68) | 49.22 (10.49) | 48.79 (10.62) | **<.001** | .01 |
| TMT-B T-score | 51.52 (9.91) | 49.62 (9.90) | 48.26 (9.72) | **<.001** | .02 |
| TMT B - A T-score | 50.41 (8.63) | 50.31 (10.99) | 49.05 (10.24) | .198 | .00 |
| Stroop-color duration T-score | 51.64 (9.81) | 48.87 (9.59) | 49.20 (10.04) | **<.001** | .02 |
| Stroop-color accuracy T-score | 51.70 (9.10) | 49.08 (10.02) | 49.04 (10.32) | **<.001** | .02 |
| VSLT 4x4 T-Score | 51.18 (9.77) | 49.37 (10.22) | 49.32 (9.85) | **.017** | .01 |
| Global T-Score | 51.44 (6.73) | 49.27 (6.83) | 48.89 (6.64) | **<.001** | .03 |

^1^Mean (SD); ANOVA used to examine cohort differences in performance; Effect size method= η²

**Supplemental Table 11**. Differences in cognition by education level among people without HIV.

|  | **Less than high school**  N = 152^1^ | **Completed high school**  N = 201^1^ | **Some College**  N = 307^1^ | **Graduated College**  N = 142^1^ | **>College**  N = 258^1^ | ***P*-value** | **η²** |
| --- | --- | --- | --- | --- | --- | --- | --- |
| TMT-A total time to completion(ms) | 23,531 (13,368) | 22,388 (13,559) | 18,676 (10,487) | 16,836 (8,296) | 17,283 (8,600) | **<.001** | .05 |
| TMT-B total time to completion(ms) | 52,198 (13,724) | 51,079 (14,144) | 43,732 (14,973) | 37,813 (13,503) | 38,738 (13,448) | **<.001** | .14 |
| TMT-B minus A(ms) | 28,667 (15,624) | 28,691 (15,320) | 25,056 (13,966) | 20,977 (12,960) | 21,455 (11,885) | **<.001** | .05 |
| Stroop Accuracy | 27.34 (4.72) | 27.80 (4.47) | 28.86 (4.19) | 29.49 (3.83) | 29.30 (3.71) | **<.001** | .03 |
| Stroop Response Time (ms) | 937.87 (230.01) | 914.16 (197.30) | 859.45 (179.41) | 844.34 (179.14) | 860.14 (176.39) | **<.001** | .03 |
| VSLT 4x4 Correct minus Incorrect | -0.79 (2.33) | -0.38 (2.28) | 0.46 (2.69) | 0.73 (2.41) | 1.11 (2.60) | **<.001** | .07 |
| TMT-A T-score | 50.00 (11.52) | 50.00 (11.16) | 50.00 (9.86) | 50.00 (8.74) | 50.00 (8.80) | >.999 | .00 |
| TMT-B T-score | 50.00 (9.22) | 50.00 (9.30) | 50.00 (10.90) | 50.00 (10.17) | 50.00 (9.72) | >.999 | .00 |
| TMT B - A T-score | 50.00 (11.31) | 50.00 (11.09) | 50.00 (10.07) | 50.00 (9.27) | 50.00 (8.45) | >.999 | .00 |
| Stroop-color duration T-score | 50.00 (10.85) | 50.00 (10.59) | 50.00 (9.73) | 50.00 (9.72) | 50.00 (9.44) | >.999 | .00 |
| Stroop-color accuracy T-score | 50.00 (11.27) | 50.00 (10.84) | 50.00 (10.18) | 50.00 (9.22) | 50.00 (8.61) | >.999 | .00 |
| VSLT 4x4 T-Score | 50.00 (9.56) | 50.00 (9.35) | 50.00 (10.61) | 50.00 (9.91) | 50.00 (10.03) | >.999 | .00 |
| Global T-Score | 50.00 (6.62) | 50.00 (6.96) | 50.00 (7.39) | 50.00 (6.49) | 50.00 (6.59) | >.999 | .00 |

^1^Mean (SD); ANOVA used to examine education differences in performance; Effect size method= η²

**Supplemental Table 12**. Clinical site differences in cognition in women with HIV.

|  | **Bronx**  N = 102^1^ | **Brooklyn**  N = 201^1^ | **Washington DC**  N = 127^1^ | **San Francisco**  N = 113^1^ | **Chicago Cook County**  N = 131^1^ | **Chapel Hill**  N = 94^1^ | **Atlanta**  N = 130^1^ | **Miami**  N = 51^1^ | **Birmingham**  N = 47^1^ | **Jackson**  N = 57^1^ | **Balti**  N =0^1^ | **Chicago-NW**  N =0^1^ | **Pitt/Ohio**  N = 0^1^ | **LA**  N = 0^1^ | ***P*-value** | η² |
| --- | --- | --- | --- | --- | --- | --- | --- | --- | --- | --- | --- | --- | --- | --- | --- | --- |
| TMT-A total time to completion(ms) | 24,288 (15,385) | 23,129 (13,490) | 19,043 (10,836) | 18,249 (10,132) | 18,516 (8,818) | 18,616 (9,636) | 20,934 (12,273) | 25,149 (16,140) | 19,846 (11,775) | 19,634 (12,488) | **-** | **-** | **-** | **-** | **<.001** | .04 |
| TMT-B total time to completion(ms) | 52,202 (13,509) | 48,757 (14,151) | 45,796 (14,299) | 42,372 (15,236) | 47,222 (14,833) | 46,703 (15,687) | 48,200 (14,474) | 50,734 (13,870) | 45,041 (15,338) | 43,641 (14,317) | **-** | **-** | **-** | **-** | **<.001** | .03 |
| TMT-B minus A(ms) | 27,913 (16,105) | 25,844 (15,070) | 26,753 (14,670) | 24,123 (13,679) | 28,705 (15,153) | 28,087 (15,299) | 27,266 (16,118) | 25,585 (14,327) | 25,195 (16,638) | 24,007 (12,908) | - | - | - | - | .322 | .01 |
| Stroop Accuracy | 26.98 (4.30) | 27.45 (4.12) | 28.46 (3.83) | 27.90 (4.42) | 27.05 (4.10) | 28.56 (3.91) | 28.38 (3.97) | 27.81 (5.12) | 28.32 (4.62) | 28.42 (5.16) | **-** | **-** | **-** | **-** | **.041** | .02 |
| Stroop Response Time (ms) | 946.16 (202.54) | 920.32 (181.83) | 891.77 (162.45) | 913.16 (179.57) | 940.92 (188.00) | 874.35 (174.96) | 876.07 (162.93) | 941.94 (251.99) | 835.46 (139.94) | 859.91 (181.14) | **-** | **-** | **-** | **-** | **<.001** | .03 |
| VSLT 4x4 Correct minus Incorrect | -0.49 (1.93) | -0.37 (2.23) | -0.12 (2.53) | 0.41 (2.36) | -0.16 (2.28) | -0.33 (2.67) | -0.15 (2.25) | -0.43 (2.50) | -0.19 (2.72) | 0.32 (2.67) | - | - | - | - | .142 | .01 |
| TMT-A T-score | 48.28 (11.84) | 47.76 (11.18) | 51.57 (9.60) | 52.39 (9.49) | 50.98 (8.99) | 50.91 (9.19) | 49.22 (9.74) | 47.24 (12.48) | 49.95 (9.81) | 50.04 (10.71) | **-** | **-** | **-** | **-** | **.001** | .03 |
| TMT-B T-score | 47.69 (8.95) | 48.52 (9.26) | 50.65 (10.21) | 53.49 (10.64) | 48.80 (10.00) | 49.56 (11.70) | 48.73 (9.02) | 48.14 (8.24) | 50.47 (11.90) | 50.57 (9.64) | **-** | **-** | **-** | **-** | **<.001** | .03 |
| TMT B - A T-score | 49.46 (11.65) | 50.32 (10.78) | 49.59 (10.60) | 51.57 (9.42) | 47.85 (10.63) | 48.42 (11.05) | 49.30 (11.26) | 51.12 (10.22) | 50.39 (12.22) | 51.20 (9.13) | - | - | - | - | .232 | .01 |
| Stroop-color duration T-score | 49.06 (10.78) | 48.25 (9.42) | 50.55 (8.93) | 49.18 (9.77) | 45.94 (9.59) | 49.51 (10.66) | 49.96 (8.95) | 47.91 (12.52) | 51.09 (9.25) | 49.28 (10.30) | **-** | **-** | **-** | **-** | **.012** | .02 |
| Stroop-color accuracy T-score | 49.21 (10.19) | 47.99 (10.31) | 51.31 (9.14) | 49.39 (10.86) | 45.86 (10.30) | 49.78 (10.29) | 49.56 (9.29) | 48.57 (12.31) | 48.80 (11.16) | 48.84 (12.52) | **-** | **-** | **-** | **-** | **.012** | .02 |
| VSLT 4x4 T-Score | 48.89 (7.56) | 48.26 (9.15) | 49.15 (9.18) | 51.43 (9.56) | 48.53 (8.85) | 47.90 (10.77) | 48.98 (9.69) | 48.92 (9.73) | 48.35 (11.13) | 49.86 (10.80) | - | - | - | - | .283 | .01 |
| Global T-Score | 48.48 (6.62) | 48.20 (6.45) | 50.48 (6.81) | 51.62 (6.80) | 48.56 (5.66) | 49.47 (7.60) | 49.22 (5.69) | 48.05 (8.10) | 49.96 (6.64) | 49.94 (7.65) | **-** | **-** | **-** | **-** | **<.001** | .03 |

^1^Mean (SD); ANOVA used to examine clinical site differences in performance; Effect size method= η²

LA=Los Angeles; DC=Washington DC; SF=San Francisco; NW=Northwestern; CC=Cook County; BHAM=Birmingham; Balti=Baltimore; Pitt=Pittsburgh

**Supplemental Table 13**. Cohort differences in cognition in women with HIV.

|  | **MACS**  N = 0^1^ | **WIHS**  N = 826^1^ | **MWCCS**  N = 194^1^ | ***P*-value** | ***d*** |
| --- | --- | --- | --- | --- | --- |
| TMT-A total time to completion(ms) | - | 20,767 (12,420) | 20,106 (10,974) | .462 | .05 |
| TMT-B total time to completion(ms) | - | 46,955 (14,645) | 48,517 (14,889) | .188 | .11 |
| TMT-B minus A(ms) | - | 26,202 (15,379) | 28,574 (13,728) | **.035** | .16 |
| Stroop Accuracy | - | 27.90 (4.19) | 27.88 (4.22) | .942 | .01 |
| Stroop Response Time (ms) | - | 904.38 (181.55) | 906.83 (185.04) | .868 | .01 |
| VSLT 4x4 Correct minus Incorrect | - | -0.16 (2.44) | -0.19 (2.09) | .870 | .01 |
| TMT-A T-score | - | 49.60 (10.69) | 50.09 (9.79) | .543 | .05 |
| TMT-B T-score | - | 49.34 (9.89) | 48.91 (9.97) | .595 | .04 |
| TMT B - A T-score | - | 49.82 (11.16) | 48.52 (9.83) | .107 | .12 |
| Stroop-color duration T-score | - | 48.72 (9.68) | 47.99 (9.95) | .358 | .08 |
| Stroop-color accuracy T-score | - | 48.64 (10.28) | 48.15 (10.66) | .563 | .05 |
| VSLT 4x4 T-Score | - | 48.85 (9.71) | 49.32 (8.14) | .489 | .05 |
| Global T-Score | - | 49.13 (6.71) | 49.08 (6.49) | .924 | .01 |

^1^Mean (SD); T-tests were used to examine cohort differences in performance; Effect size method= Cohen’s *d*

**Supplemental Table 14**. Differences in cognition by education level in women with HIV.

|  | **Less than high school**  N = 245^1^ | **Completed high school**  N = 256^1^ | **Some College**  N = 382^1^ | **Graduated College**  N = 18^1^ | **>College**  N = 149^1^ | ***P*-value** | **η²** |
| --- | --- | --- | --- | --- | --- | --- | --- |
| TMT-A total time to completion(ms) | 24,641 (14,753) | 21,598 (12,513) | 19,557 (11,272) | 14,893 (7,490) | 16,549 (7,542) | **<.001** | .05 |
| TMT-B total time to completion(ms) | 53,515 (14,130) | 49,534 (13,52) | 44,646 (14,235) | 39,618 (13,263) | 40,322 (14,255) | **<.001** | .10 |
| TMT-B minus A(ms) | 28,874 (15,816) | 28,107 (14,799) | 25,089 (15,241) | 24,725 (14,037) | 23,773 (13,249) | **.002** | .02 |
| Stroop Accuracy | 26.23 (4.92) | 27.36 (3.87) | 28.37 (3.80) | 28.82 (2.99) | 29.48 (3.79) | **<.001** | .07 |
| Stroop Response Time (ms) | 962.00 (214.22) | 929.28 (181.82) | 878.47 (157.43) | 864.78 (117.19) | 845.88 (172.73) | **<.001** | .05 |
| VSLT 4x4 Correct minus Incorrect | -0.71 (2.02) | -0.56 (2.32) | 0.08 (2.46) | 0.33 (2.14) | 0.72 (2.46) | **<.001** | .04 |
| TMT-A T-score | 49.46 (11.72) | 50.92 (10.91) | 49.44 (9.89) | 51.28 (8.96) | 49.32 (8.10) | .355 | .00 |
| TMT-B T-score | 49.63 (9.78) | 51.12 (9.21) | 49.51 (10.24) | 45.99 (9.81) | 47.30 (10.62) | **.003** | .02 |
| TMT B - A T-score | 50.08 (11.43) | 50.39 (10.53) | 50.17 (10.94) | 45.85 (9.44) | 47.62 (9.30) | **.041** | .01 |
| Stroop-color duration T-score | 49.17 (11.44) | 49.32 (10.40) | 48.86 (8.47) | 44.57 (7.88) | 48.39 (9.81) | .343 | .00 |
| Stroop-color accuracy T-score | 48.02 (12.94) | 49.32 (10.18) | 49.35 (9.35) | 45.55 (6.85) | 48.53 (9.12) | .299 | .00 |
| VSLT 4x4 T-Score | 50.45 (8.23) | 49.47 (9.29) | 48.62 (9.94) | 46.53 (9.03) | 46.91 (10.10) | **.004** | .01 |
| Global T-Score | 49.68 (7.22) | 50.21 (6.80) | 49.11 (6.30) | 47.09 (6.51) | 47.98 (6.49) | **.009** | .01 |

^1^Mean (SD); ANOVA used to examine education differences in performance; Effect size method= η²

**Supplemental Table 15**. Differences in cognition by STRAW+10 menopause status in women with HIV.

|  | **Premenopausal**  N = 160^1^ | **Perimenopause**  N = 85^1^ | **Post Menopausal**  N = 698^1^ | ***P*-value** | **η²** |
| --- | --- | --- | --- | --- | --- |
| TMT-A total time to completion(ms) | 17,642 (10,372) | 17,412 (9,395) | 21,700 (12,771) | **<.001** | .02 |
| TMT-B total time to completion(ms) | 44,244 (14,295) | 41,673 (14,403) | 48,582 (14,572) | **<.001** | .03 |
| TMT-B minus A(ms) | 26,602 (14,493) | 24,260 (14,848) | 26,899 (15,385) | .319 | .00 |
| Stroop Accuracy | 29.49 (4.34) | 29.15 (3.45) | 27.31 (4.11) | **<.001** | .05 |
| Stroop Response Time (ms) | 835.56 (187.44) | 829.75 (120.93) | 931.88 (179.29) | **<.001** | .06 |
| VSLT 4x4 Correct minus Incorrect | 0.23 (2.67) | 0.69 (2.45) | -0.37 (2.29) | **<.001** | .02 |
| TMT-A T-score | 50.30 (10.04) | 50.82 (9.54) | 49.53 (10.84) | .451 | .00 |
| TMT-B T-score | 47.58 (10.04) | 50.47 (10.60) | 49.55 (9.68) | **.038** | .01 |
| TMT B - A T-score | 47.91 (10.53) | 50.05 (10.77) | 49.95 (11.08) | .101 | .00 |
| Stroop-color duration T-score | 47.62 (10.63) | 48.94 (8.21) | 48.66 (9.57) | .428 | .00 |
| Stroop-color accuracy T-score | 47.83 (10.74) | 48.39 (9.14) | 48.58 (10.35) | .709 | .00 |
| VSLT 4x4 T-Score | 48.15 (10.61) | 50.45 (9.75) | 48.89 (9.15) | .195 | .00 |
| Global T-Score | 48.41 (7.18) | 50.17 (6.11) | 49.16 (6.55) | .137 | .00 |

^1^Mean (SD); ANOVA used to examine education differences in performance; Effect size method= η²

**Supplemental Table 16**. Differences in cognition by viral load status in women with HIV.

| **Characteristic** | **<=20**  N = 572^1^ | **21-200**  N = 317^1^ | **201-500**  N = 15^1^ | **501-1000**  N = 6^1^ | **>1000**  N = 47^1^ | ***P*-value** | η² |
| --- | --- | --- | --- | --- | --- | --- | --- |
| TMT-A total time to completion(ms) | 20,725 (12,227) | 21,446 (12,287) | 19,626 (13,797) | 19,590 (9,153) | 16,688 (8,248) | .163 | .01 |
| TMT-B total time to completion(ms) | 46,158 (14,672) | 49,178 (14,566) | 43,611 (15,067) | 47,046 (18,091) | 47,168 (14,993) | **.050** | .01 |
| TMT-B minus A(ms) | 25,509 (15,335) | 27,732 (14,828) | 23,985 (13,284) | 27,457 (16,502) | 30,480 (14,332) | .083 | .01 |
| Stroop Accuracy | 28.13 (4.09) | 27.51 (4.32) | 26.40 (4.97) | 26.83 (6.46) | 28.00 (3.61) | .144 | .01 |
| Stroop Response Time (ms) | 898.88 (177.44) | 920.54 (194.77) | 952.37 (179.05) | 859.07 (221.67) | 892.76 (138.13) | .341 | .00 |
| VSLT 4x4 Correct minus Incorrect | -0.02 (2.41) | -0.38 (2.29) | 0.13 (2.29) | 0.00 (2.00) | -0.04 (2.05) | .295 | .01 |
| TMT-A T-score | 49.56 (10.45) | 48.98 (10.73) | 50.18 (11.29) | 49.51 (9.30) | 53.21 (8.89) | .152 | .01 |
| TMT-B T-score | 49.98 (9.83) | 48.21 (9.81) | 49.93 (9.10) | 49.55 (10.68) | 48.82 (10.83) | .149 | .01 |
| TMT B - A T-score | 50.37 (11.03) | 48.95 (10.75) | 50.60 (9.43) | 48.94 (11.43) | 46.60 (10.54) | .108 | .01 |
| Stroop-color duration T-score | 48.98 (9.52) | 48.01 (10.24) | 44.10 (9.98) | 49.17 (10.18) | 47.67 (7.50) | .228 | .01 |
| Stroop-color accuracy T-score | 49.22 (9.98) | 47.79 (10.82) | 43.09 (12.80) | 43.53 (16.04) | 47.83 (9.54) | **.042** | .01 |
| VSLT 4x4 T-Score | 49.47 (9.62) | 48.25 (9.09) | 48.94 (9.19) | 49.67 (6.04) | 49.07 (8.24) | .486 | .00 |
| Global T-Score | 49.50 (6.65) | 48.36 (6.72) | 48.29 (6.74) | 49.48 (6.45) | 49.69 (6.01) | .164 | .01 |

^1^Mean (SD); ANOVA used to examine viral load differences in performance; Effect size method= η²

**Supplemental Table 17**. Clinical site differences in cognition in women without HIV.

|  | **Bronx**  N = 66^1^ | **Brooklyn**  N = 78^1^ | **DC**  N = 53^1^ | **SF**  N = 61^1^ | **Chicago-CC** N = 48^1^ | **Chapel Hill**  N = 38^1^ | **Atlanta**  N = 70^1^ | **Miami**  N = 44^1^ | **BHAM**  N = 16^1^ | **Jackson**  N = 25^1^ | **Balti**  N = 0^1^ | **Chicago-NW**  N = 0^1^ | **Pitt/**  **Ohio**  N = 0^1^ | **LA**  N = 0^1^ | ***P*-value** | η² |
| --- | --- | --- | --- | --- | --- | --- | --- | --- | --- | --- | --- | --- | --- | --- | --- | --- |
| TMT-A total time to completion(ms) | 21,036 (12,860) | 23,710 (14,156) | 21,354 (13,442) | 20,634 (11,940) | 18,582 (10,398) | 19,738 (10,430) | 19,522 (12,741) | 23,555 (15,566) | 23,720 (18,636) | 22,698 (14,025) |  | - | - | - | .461 | .02 |
| TMT-B total time to completion(ms) | 47,681 (14,891) | 50,505 (14,005) | 45,601 (15,255) | 43,730 (14,269) | 49,064 (15,390) | 45,389 (15,850) | 44,846 (15,293) | 48,865 (15,013) | 44,191 (17,933) | 42,914 (16,800) |  | - | - | - | .154 | .03 |
| TMT-B minus A(ms) | 26,645 (15,283) | 26,796 (16,692) | 24,247 (16,381) | 23,097 (14,779) | 30,482 (14,868) | 25,651 (15,701) | 25,324 (14,676) | 25,310 (15,372) | 20,471 (17,832) | 20,216 (14,371) |  | - | - | - | .204 | .02 |
| Stroop Accuracy | 27.92 (4.96) | 27.97 (3.77) | 29.70 (4.15) | 28.87 (4.13) | 27.54 (2.87) | 28.39 (4.76) | 28.24 (4.16) | 26.93 (4.41) | 28.00 (7.00) | 28.83 (4.36) |  | - | - | - | .281 | .03 |
| Stroop Response Time (ms) | 921.55 (249.96) | 920.71 (200.62) | 836.97 (149.29) | 868.58 (161.14) | 937.49 (159.82) | 852.90 (201.40) | 874.73 (157.94) | 950.47 (212.58) | 814.05 (202.01) | 805.52 (108.84) |  | - | - | - | **.002** | .05 |
| VSLT 4x4 Correct minus Incorrect | -0.56 (2.21) | -0.50 (2.42) | 0.40 (2.63) | 0.54 (2.74) | -0.98 (2.44) | -0.37 (2.56) | 0.61 (2.66) | -0.32 (2.47) | 0.25 (2.54) | 0.68 (3.25) |  | - | - | - | **.004** | .05 |
| TMT-A T-score | 50.53 (10.74) | 47.35 (10.58) | 48.37 (11.12) | 48.83 (9.82) | 52.03 (9.01) | 49.39 (9.16) | 50.73 (10.71) | 48.21 (12.39) | 48.14 (15.08) | 44.61 (10.95) |  | - | - | - | .140 | .03 |
| TMT-B T-score | 50.40 (10.12) | 47.78 (8.25) | 49.05 (10.33) | 50.61 (8.92) | 48.78 (9.85) | 50.17 (10.73) | 50.34 (10.17) | 49.43 (9.91) | 51.66 (15.47) | 48.50 (11.33) |  | - | - | - | .782 | .01 |
| TMT B - A T-score | 50.13 (10.86) | 50.00 (11.87) | 50.44 (11.30) | 51.69 (10.53) | 47.12 (10.45) | 50.11 (10.97) | 50.25 (10.43) | 51.28 (11.02) | 53.59 (13.09) | 52.23 (10.28) |  | - | - | - | .578 | .02 |
| Stroop-color duration T-score | 49.93 (11.86) | 48.12 (9.40) | 52.15 (7.81) | 49.80 (8.55) | 47.25 (9.22) | 50.46 (10.62) | 48.64 (9.28) | 46.65 (11.36) | 52.19 (11.10) | 48.17 (7.25) |  | - | - | - | .127 | .03 |
| Stroop-color accuracy T-score | 49.65 (11.69) | 49.32 (8.54) | 52.22 (9.77) | 50.14 (8.81) | 48.69 (7.61) | 48.83 (11.92) | 47.80 (9.92) | 47.41 (11.45) | 51.22 (11.44) | 47.42 (10.23) |  | - | - | - | .365 | .02 |
| VSLT 4x4 T-Score | 48.22 (9.35) | 48.08 (9.79) | 49.94 (9.97) | 51.14 (10.50) | 45.98 (9.21) | 47.60 (9.85) | 51.58 (10.09) | 49.46 (10.27) | 49.64 (10.35) | 49.54 (13.22) |  | - | - | - | .130 | .03 |
| Global T-Score | 49.77 (6.92) | 47.83 (6.10) | 49.88 (6.30) | 50.10 (6.74) | 48.51 (5.79) | 49.41 (6.48) | 50.32 (6.57) | 48.43 (8.22) | 50.41 (9.68) | 47.70 (7.47) |  | - | - | - | .361 | .02 |

^1^Mean (SD); ANOVA used to examine clinical site differences in performance; Effect size method= η²

LA=Los Angeles; DC=Washington DC; SF=San Francisco; NW=Northwestern; CC=Cook County; BHAM=Birmingham; Balti=Baltimore; Pitt=Pittsburgh

**Supplemental Table 18**. Cohort differences in cognition in women without HIV.

|  | **MACS**  N = 0^1^ | **WIHS**  N = 359^1^ | **MWCCS**  N =111^1^ | ***P*-value** | ***d*** |
| --- | --- | --- | --- | --- | --- |
| TMT-A total time to completion(ms) | - | 20,812 (12,444) | 22,745(14,484) | .206 | .15 |
| TMT-B total time to completion(ms) | - | 46,169 (15,136) | 48,702 (15,365) | .129 | .17 |
| TMT-B minus A(ms) | - | 25,357 (15,293) | 25,957 (16,257) | .731 | .04 |
| Stroop Accuracy | - | 28.32 (4.30) | 28.17 (3.78) | .726 | .04 |
| Stroop Response Time (ms) | - | 890.59 (194.23) | 879.61 (170.25) | .567 | .06 |
| VSLT 4x4 Correct minus Incorrect | - | 0.03 (2.63) | -0.45 (2.53) | .084 | .18 |
| TMT-A T-score | - | 49.22 (10.49) | 47.75 (12.01) | .250 | .14 |
| TMT-B T-score | - | 49.62 (9.90) | 48.16 (9.65) | .168 | .15 |
| TMT B - A T-score | - | 50.31 (10.99) | 50.01 (11.37) | .807 | .03 |
| Stroop-color duration T-score | - | 48.87 (9.59) | 49.11 (9.03) | .812 | .03 |
| Stroop-color accuracy T-score | - | 49.08 (10.02) | 48.84 (9.19) | .818 | .02 |
| VSLT 4x4 T-Score | - | 49.37 (10.22) | 47.94 (9.79) | .187 | .14 |
| Global T-Score | - | 49.27 (6.83) | 48.24 (6.39) | .147 | .15 |

^1^Mean (SD); T-tests were used to examine cohort differences in performance; Effect size method= Cohen’s *d*

**Supplemental Table 19**. Differences in cognition by education level in women without HIV.

|  | **Less than high school**  N = 108^1^ | **Completed high school**  N = 126^1^ | **Some College**  N = 191^1^ | **Graduated College**  N = 17^1^ | **>College**  N = 55^1^ | ***P*-value** | **η²** |
| --- | --- | --- | --- | --- | --- | --- | --- |
| TMT-A total time to completion(ms) | 24,104 (14,113) | 23,750 (14,518) | 19,618 (12,027) | 17,414 (11,774) | 17,424 (10,112) | **<.001** | .04 |
| TMT-B total time to completion(ms) | 52,056 (13,739) | 51,201 (14,048) | 44,349 (15,155) | 35,803 (12,963) | 38,162 (13,930) | **<.001** | .11 |
| TMT-B minus A(ms) | 27,952 (16,441) | 27,451 (16,396) | 24,731 (14,993) | 18,389 (12,891) | 20,738 (13,363) | **.008** | .03 |
| Stroop Accuracy | 26.80 (4.43) | 27.73 (4.33) | 28.86 (4.05) | 30.00 (2.10) | 30.00 (2.95) | **<.001** | .06 |
| Stroop Response Time (ms) | 960.60 (233.77) | 913.13 (185.16) | 858.79 (174.82) | 819.47 (86.43) | 823.58 (126.05) | **<.001** | .06 |
| VSLT 4x4 Correct minus Incorrect | -0.59 (2.45) | -0.70 (2.24) | 0.19 (2.63) | 1.35 (3.00) | 1.13 (2.78) | **<.001** | .06 |
| TMT-A T-score | 49.70 (11.68) | 48.93 (11.27) | 49.23 (10.22) | 48.32 (9.98) | 48.16 (9.94) | .925 | .00 |
| TMT-B T-score | 50.28 (9.02) | 49.81 (9.45) | 49.52 (10.89) | 48.81 (9.68) | 47.84 (9.92) | .668 | .00 |
| TMT B - A T-score | 50.70 (11.69) | 50.74 (11.85) | 50.24 (10.67) | 50.21 (8.96) | 49.37 (9.35) | .949 | .00 |
| Stroop-color duration T-score | 49.16 (10.73) | 49.78 (10.01) | 49.50 (9.60) | 46.52 (6.24) | 47.46 (8.71) | .459 | .01 |
| Stroop-color accuracy T-score | 49.08 (10.89) | 49.71 (10.64) | 49.55 (10.02) | 47.37 (5.65) | 48.15 (7.66) | .786 | .00 |
| VSLT 4x4 T-Score | 50.87 (10.05) | 48.72 (9.24) | 48.74 (10.37) | 50.41 (12.13) | 47.90 (10.72) | .316 | .01 |
| Global T-Score | 50.00 (6.82) | 49.31 (6.72) | 49.25 (6.87) | 48.51 (6.13) | 47.84 (6.80) | .421 | .01 |

^1^Mean (SD); ANOVA used to examine education differences in performance; Effect size method= η²

**Supplemental Table 20**. Differences in cognition by STRAW+10 menopause status in women without HIV.

|  | **Premenopausal**  N = 110^1^ | **Perimenopause**  N = 55^1^ | **Post Menopausal**  N = 263^1^ | ***P*-value** | **η²** |
| --- | --- | --- | --- | --- | --- |
| TMT-A total time to completion(ms) | 16,824 (8,537) | 17,521 (9,664) | 23,348 (14,380) | **<.001** | .06 |
| TMT-B total time to completion(ms) | 40,746 (14,486) | 42,265 (15,584) | 49,845 (14,645) | **<.001** | .08 |
| TMT-B minus A(ms) | 23,922 (13,867) | 24,744 (14,981) | 26,497 (15,975) | .304 | .01 |
| Stroop Accuracy | 29.65 (3.76) | 29.35 (3.77) | 27.60 (4.13) | **<.001** | .05 |
| Stroop Response Time (ms) | 804.50 (122.52) | 831.77 (157.99) | 930.45 (195.15) | **<.001** | .10 |
| VSLT 4x4 Correct minus Incorrect | 0.65 (3.06) | 0.47 (2.87) | -0.55 (2.23) | **<.001** | .05 |
| TMT-A T-score | 50.64 (9.08) | 50.83 (9.45) | 48.34 (11.64) | .088 | .01 |
| TMT-B T-score | 50.38 (9.78) | 50.45 (10.19) | 48.85 (10.07) | .300 | .01 |
| TMT B - A T-score | 49.95 (9.62) | 49.77 (10.22) | 50.32 (11.71) | .922 | .00 |
| Stroop-color duration T-score | 48.98 (7.98) | 49.24 (9.61) | 48.96 (9.83) | .980 | .00 |
| Stroop-color accuracy T-score | 48.55 (9.19) | 49.00 (9.75) | 49.48 (9.96) | .696 | .00 |
| VSLT 4x4 T-Score | 49.95 (11.88) | 49.82 (10.72) | 48.28 (9.18) | .273 | .01 |
| Global T-Score | 49.99 (6.57) | 50.08 (6.98) | 48.61 (6.78) | .111 | .01 |

^1^Mean (SD); ANOVA used to examine education differences in performance; Effect size method= η²

**Supplemental Table 21**. Clinical site differences in cognition in men with HIV.

|  | **Bronx**  N = 29^1^ | **Brooklyn**  N = 0^1^ | **DC**  N = 1^1^ | **SF**  N = 24^1^ | **Chicago-CC** N = 0^1^ | **Chapel Hill**  N = 97^1^ | **Atlanta**  N = 76^1^ | **Miami**  N = 82^1^ | **BHAM**  N = 43^1^ | **Jackson**  N = 31^1^ | **Balti**  N = 136^1^ | **Chicago-NW**  N = 128^1^ | **Pitt/**  **Ohio**  N =58^1^ | **LA**  N = 116^1^ | ***P*-value** | η² |
| --- | --- | --- | --- | --- | --- | --- | --- | --- | --- | --- | --- | --- | --- | --- | --- | --- |
| TMT-A total time to completion(ms) | 19,142 (10,692) | - | 17,320 | 15,692 (5,221) | - | 15,980 (8,294) | 20,354 (10,968) | 18,731 (10,306) | 17,100 (8,365) | 19,121 (9,551) | 18,066 (8,559) | 17,568 (7,142) | 15,226 (5,650) | 18,480 (9,618) | **.030** | .03 |
| TMT-B total time to completion(ms) | 46,820 (14,553) | - | 32,583 | 37,719 (13,007) | - | 38,153 (14,427) | 48,637 (14,803) | 42,417 (15,440) | 42,224 (15,916) | 48,719 (15,523) | 43,758 (14,460) | 42,279 (14,287) | 38,333 (14,557) | 45,188 (14,704) | **<.001** | .06 |
| TMT-B minus A(ms) | 27,678 (13,405) | - | 15,263 | 22,027 (11,169) | - | 22,173 (13,844) | 28,283 (15,330) | 23,687 (13,552) | 25,124 (15,129) | 29,597 (12,169) | 25,691 (13,094) | 24,793 (12,541) | 23,106 (13,084) | 26,708 (13,202) | **.043** | .03 |
| Stroop Accuracy | 26.79 (5.31) | - | - | 30.33 (4.51) | - | 30.52 (4.08) | 27.56 (4.89) | 26.44 (5.03) | 27.81 (4.87) | 27.45 (6.27) | 29.07 (4.43) | 27.90 (4.28) | 30.23 (3.02) | 28.58 (4.37) | **<.001** | .07 |
| Stroop Response Time (ms) | 860.65 (210.12) | - | 807.97 | 803.94 (182.36) | - | 790.84 (170.92) | 901.14 (242.57) | 858.01 (206.78) | 820.17 (180.50) | 834.19 (193.49) | 855.09 (192.33) | 925.33 (206.34) | 814.71 (135.44) | 871.85 (191.36) | **<.001** | .05 |
| VSLT 4x4 Correct minus Incorrect | 0.21 (2.66) | - | 0.00 | 0.67 (3.16) | - | 1.06 (2.63) | -0.18 (2.54) | 0.48 (3.04) | 0.16 (2.62) | 0.19 (2.70) | 0.35 (2.63) | 0.38 (2.54) | 1.48 (2.82) | 0.64 (2.51) | **.042** | .03 |
| TMT-A T-score | 49.99 (8.92) | - | 47.16 | 52.67 (8.25) | - | 51.13 (7.95) | 48.27 (10.16) | 50.24 (9.27) | 49.97 (9.44) | 46.87 (9.71) | 50.84 (8.30) | 50.96 (7.53) | 53.36 (7.29) | 50.59 (8.47) | **.031** | .03 |
| TMT-B T-score | 48.31 (8.84) | - | 54.76 | 53.41 (11.67) | - | 51.58 (8.85) | 46.61 (9.86) | 50.95 (10.30) | 50.17 (14.13) | 42.54 (12.28) | 50.18 (10.08) | 50.53 (9.75) | 52.84 (10.60) | 49.36 (9.55) | **<.001** | .05 |
| TMT B - A T-score | 48.37 (9.19) | - | 56.10 | 51.11 (8.03) | - | 50.54 (8.79) | 47.68 (10.63) | 50.48 (9.26) | 49.38 (11.12) | 44.65 (9.38) | 49.27 (9.14) | 49.40 (8.61) | 49.96 (8.84) | 48.81 (9.13) | .116 | .02 |
| Stroop-color duration T-score | 48.79 (9.88) | - | 47.57 | 54.16 (10.57) | - | 50.75 (9.93) | 46.97 (12.18) | 50.69 (10.30) | 47.18 (11.73) | 46.01 (11.54) | 52.50 (11.42) | 48.00 (9.42) | 53.74 (7.41) | 49.86 (10.53) | **<.001** | .04 |
| Stroop-color accuracy T-score | 46.82 (13.30) | - | 43.58 | 55.36 (9.70) | - | 51.22 (9.48) | 45.86 (14.65) | 50.87 (10.17) | 45.44 (13.99) | 42.08 (17.30) | 51.85 (11.73) | 48.35 (9.19) | 54.02 (6.64) | 49.88 (10.44) | **<.001** | .07 |
| VSLT 4x4 T-Score | 49.91 (9.80) | - | 46.47 | 50.64 (12.39) | - | 50.81 (9.62) | 47.84 (9.87) | 50.21 (11.52) | 48.36 (11.07) | 47.09 (10.80) | 50.09 (10.34) | 49.19 (10.00) | 53.33 (11.49) | 51.18 (9.93) | .111 | .02 |
| Global T-Score | 49.25 (6.12) | - | 48.99 | 52.72 (8.47) | - | 51.07 (6.04) | 47.42 (6.61) | 50.52 (7.45) | 48.92 (8.19) | 45.63 (7.67) | 50.90 (6.97) | 49.67 (6.53) | 53.32 (6.35) | 50.25 (7.23) | **<.001** | .06 |

^1^Mean (SD); ANOVA used to examine clinical site differences in performance; Effect size method= η²

LA=Los Angeles; DC=Washington DC; SF=San Francisco; NW=Northwestern; CC=Cook County; BHAM=Birmingham; Balti=Baltimore; Pitt=Pittsburgh

**Supplemental Table 22**. Cohort differences in cognition in men with HIV.

|  | **MACS**  N = 410^1^ | **WIHS**  N = 0^1^ | **MWCCS**  N =315^1^ | ***P*-value** | ***d*** |
| --- | --- | --- | --- | --- | --- |
| TMT-A total time to completion(ms) | 17,708 (8,273) | - | 17,779 (9,466) | .916 | .01 |
| TMT-B total time to completion(ms) | 42,876 (14,528) | - | 42,656 (15,563) | .846 | .01 |
| TMT-B minus A(ms) | 25,194 (12,904) | - | 24,876 (14,327) | .758 | .02 |
| Stroop Accuracy | 28.76 (4.23) | - | 29.12 (4.98) | .312 | .08 |
| Stroop Response Time (ms) | 877.39 (191.80) | - | 845.87 (210.12) | **.038** | .16 |
| VSLT 4x4 Correct minus Incorrect | 0.56 (2.62) | - | 0.50 (2.73) | .774 | .02 |
| TMT-A T-score | 51.00 (8.25) | - | 50.00 (9.07) | .127 | .12 |
| TMT-B T-score | 50.33 (10.02) | - | 49.54 (10.72) | .311 | .08 |
| TMT B - A T-score | 49.37 (9.05) | - | 49.26 (9.96) | .870 | .01 |
| Stroop-color duration T-score | 50.30 (10.39) | - | 48.57 (11.04) | **.032** | .16 |
| Stroop-color accuracy T-score | 50.41 (10.36) | - | 47.98 (12.35) | **.005** | .22 |
| VSLT 4x4 T-Score | 50.62 (10.31) | - | 49.62 (10.39) | .195 | .10 |
| Global T-Score | 50.56 (7.01) | - | 49.43 (7.23) | **.035** | .16 |

^1^Mean (SD); T-tests were used to examine cohort differences in performance; Effect size method= Cohen’s *d*

**Supplemental Table 23**. Differences in cognition by education level in men with HIV.

|  | **Less than high school**  N =73^1^ | **Completed high school**  N = 172^1^ | **Some College**  N = 253^1^ | **Graduated College**  N = 162^1^ | **>College**  N = 157^1^ | ***P*-value** | **η²** |
| --- | --- | --- | --- | --- | --- | --- | --- |
| TMT-A total time to completion(ms) | 21,280 (11,381) | 19,376 (9,207) | 17,835 (9,512) | 16,086 (7,113) | 16,314 (6,731) | **<.001** | .03 |
| TMT-B total time to completion(ms) | 51,661 (12,706) | 48,093 (14,793) | 43,038 (15,224) | 38,332 (14,084) | 37,900 (12,784) | **<.001** | .10 |
| TMT-B minus A(ms) | 30,380 (12,173) | 28,778 (13,925) | 25,203 (14,394) | 22,246 (11,810) | 21,586 (11,917) | **<.001** | .05 |
| Stroop Accuracy | 27.66 (4.55) | 28.16 (4.73) | 29.53 (4.37) | 29.79 (4.88) | 28.87 (4.37) | **<.001** | .02 |
| Stroop Response Time (ms) | 915.49 (215.36) | 884.49 (204.08) | 830.70 (192.32) | 828.06 (195.13) | 876.02 (184.84) | **<.001** | .02 |
| VSLT 4x4 Correct minus Incorrect | -0.53 (2.28) | -0.06 (2.52) | 0.55 (2.71) | 0.80 (2.85) | 1.24 (2.59) | **<.001** | .04 |
| TMT-A T-score | 50.86 (9.41) | 51.30 (8.72) | 50.50 (9.62) | 49.58 (7.92) | 49.94 (7.65) | .417 | .00 |
| TMT-B T-score | 49.24 (8.08) | 51.17 (10.04) | 50.16 (10.83) | 48.21 (10.89) | 49.60 (9.62) | .107 | .01 |
| TMT B - A T-score | 48.39 (8.75) | 49.51 (9.86) | 49.72 (10.19) | 48.41 (8.61) | 49.48 (8.38) | .593 | .00 |
| Stroop-color duration T-score | 49.79 (11.56) | 50.33 (10.44) | 51.40 (10.65) | 48.84 (10.50) | 47.53 (9.85) | **.005** | .02 |
| Stroop-color accuracy T-score | 49.62 (12.28) | 49.56 (11.84) | 51.22 (11.05) | 48.58 (12.31) | 47.44 (9.98) | **.019** | .01 |
| VSLT 4x4 T-Score | 50.50 (9.21) | 50.70 (9.94) | 50.11 (10.60) | 49.27 (11.27) | 49.89 (10.21) | .782 | .00 |
| Global T-Score | 50.10 (6.99) | 50.88 (7.00) | 50.54 (7.22) | 48.97 (7.39) | 49.24 (6.35) | .051 | .01 |

^1^Mean (SD); ANOVA used to examine education differences in performance; Effect size method= η²

**Supplemental Table 24**. Differences in cognition by viral load status in men with HIV.

| **Characteristic** | **<=20**  N = 359^1^ | **21-200**  N = 240^1^ | **201-500**  N = 9^1^ | **501-1000**  N = 5^1^ | **>1000**  N = 32^1^ | ***P*-value** | **η²** |
| --- | --- | --- | --- | --- | --- | --- | --- |
| TMT-A total time to completion(ms) | 17,520 (8,842) | 17,166 (8,130) | 15,246 (3,688) | 22,244 (11,477) | 16,488 (4,924) | .582 | .00 |
| TMT-B total time to completion(ms) | 41,211 (14,818) | 43,042 (15,329) | 43,830 (13,577) | 58,561 (12,391) | 46,518 (14,105) | **.027** | .02 |
| TMT-B minus A(ms) | 23,721 (13,209) | 25,877 (13,786) | 28,584 (14,177) | 36,317 (10,133) | 30,030 (13,144) | **.012** | .02 |
| Stroop Accuracy | 28.99 (4.63) | 29.12 (4.59) | 29.33 (2.69) | 27.80 (4.44) | 27.41 (4.54) | .363 | .01 |
| Stroop Response Time (ms) | 856.22 (184.64) | 857.26 (220.61) | 846.68 (106.53) | 966.58 (226.64) | 899.44 (182.89) | .571 | .00 |
| VSLT 4x4 Correct minus Incorrect | 0.55 (2.74) | 0.56 (2.65) | 0.44 (1.88) | -0.40 (1.52) | 0.59 (2.37) | .957 | .00 |
| TMT-A T-score | 50.57 (8.79) | 51.13 (8.04) | 52.60 (6.71) | 50.01 (10.48) | 50.90 (6.54) | .895 | .00 |
| TMT-B T-score | 50.59 (10.06) | 50.06 (10.56) | 48.04 (12.50) | 45.42 (5.03) | 47.00 (11.04) | .281 | .01 |
| TMT B - A T-score | 49.96 (9.10) | 48.85 (9.55) | 46.39 (11.46) | 44.40 (6.41) | 45.92 (9.95) | .067 | .01 |
| Stroop-color duration T-score | 49.53 (10.09) | 50.19 (11.56) | 50.31 (5.47) | 47.54 (9.37) | 45.31 (11.67) | .192 | .01 |
| Stroop-color accuracy T-score | 49.19 (11.47) | 49.91 (11.28) | 50.88 (4.34) | 50.58 (7.74) | 43.97 (13.48) | .097 | .01 |
| VSLT 4x4 T-Score | 49.91 (10.67) | 50.38 (10.17) | 49.66 (8.53) | 50.86 (4.12) | 50.50 (9.60) | .984 | .00 |
| Global T-Score | 50.15 (7.01) | 50.44 (7.28) | 50.15 (6.01) | 48.46 (6.27) | 48.43 (7.23) | .635 | .00 |

^1^Mean (SD); ANOVA used to examine viral load differences in performance; Effect size method= η²

**Supplemental Table 25**. Clinical site differences in cognition in men without HIV.

|  | **Bronx**  N = 28^1^ | **Brooklyn**  N = 0^1^ | **DC**  N = 0^1^ | **SF**  N = 31^1^ | **Chicago-CC** N = 0^1^ | **Chapel Hill**  N = 16^1^ | **Atlanta**  N = 21^1^ | **Miami**  N = 23^1^ | **BHAM**  N = 20^1^ | **Jackson**  N = 18^1^ | **Balti** = 117^1^ | **Chicago-NW**  N = 85^1^ | **Pitt/**  **Ohio**  N =93^1^ | **LA**  N = 112^1^ | ***P*-value** | η² |
| --- | --- | --- | --- | --- | --- | --- | --- | --- | --- | --- | --- | --- | --- | --- | --- | --- |
| TMT-A total time to completion(ms) | 19,859 (11,351) | - | - | 17,647 (8,313) | - | 17,402 (6,004) | 18,100 (6,164) | 24,389 (16,076) | 16,111 (5,805) | 22,079 (13,036) | 17,089 (6,800) | 17,923 (8,510) | 16,938 (8,017) | 17,482 (8,871) | **.034** | .04 |
| TMT-B total time to completion(ms) | 47,582 (15,775) | - | - | 42,113 (14,814) | - | 52,718 (13,221) | 51,196 (13,538) | 52,084 (15,047) | 35,354 (12,715) | 49,406 (16,184) | 40,581 (14,402) | 41,812 (15,030) | 38,742 (13,195) | 40,611 (13,832) | **<.001** | .10 |
| TMT-B minus A(ms) | 27,723 (12,596) | - | - | 24,465 (11,616) | - | 35,316 (9,508) | 33,096 (13,320) | 27,695 (15,727) | 19,243 (13,891) | 27,327 (12,033) | 23,492 (12,472) | 23,889 (12,964) | 21,805 (11,241) | 23,128 (12,839) | **<.001** | .07 |
| Stroop Accuracy | 30.20 (4.86) | - | - | 28.90 (4.25) | - | 28.71 (5.85) | 25.88 (4.56) | 28.40 (3.89) | 31.83 (4.11) | 29.50 (7.56) | 29.08 (4.05) | 28.48 (4.16) | 28.69 (3.74) | 29.59 (3.79) | **.016** | .06 |
| Stroop Response Time (ms) | 845.96 (234.52) | - | - | 891.61 (212.04) | - | 877.26 (242.16) | 953.35 (212.92) | 934.42 (157.12) | 718.30 (161.05) | 885.63 (244.24) | 860.11 (178.12) | 895.53 (208.05) | 878.80 (197.23) | 853.51 (164.02) | **.013** | .05 |
| VSLT 4x4 Correct minus Incorrect | -0.36 (2.16) | - | - | 0.45 (2.42) | - | 0.88 (3.05) | 0.29 (2.70) | -0.43 (2.57) | 0.85 (2.76) | 0.28 (2.89) | 0.43 (2.58) | 0.91 (2.32) | 1.23 (2.56) | 0.75 (2.48) | .073 | .04 |
| TMT-A T-score | 51.51 (10.12) | - | - | 52.24 (10.42) | - | 52.14 (7.06) | 51.83 (6.58) | 47.19 (12.01) | 48.81 (8.34) | 46.24 (10.20) | 51.73 (7.83) | 50.47 (8.37) | 51.75 (8.04) | 51.87 (8.89) | .144 | .03 |
| TMT-B T-score | 50.81 (9.37) | - | - | 52.21 (12.12) | - | 46.09 (7.65) | 47.57 (8.43) | 46.73 (9.58) | 51.14 (12.54) | 44.52 (9.22) | 51.80 (10.69) | 49.90 (10.19) | 52.14 (9.65) | 51.80 (9.16) | **.016** | .05 |
| TMT B - A T-score | 49.51 (8.15) | - | - | 50.30 (8.01) | - | 44.03 (6.22) | 45.55 (9.27) | 48.97 (10.89) | 51.35 (10.60) | 47.41 (8.44) | 50.07 (8.84) | 49.13 (8.84) | 50.82 (7.95) | 50.42 (8.56) | .057 | .04 |
| Stroop-color duration T-score | 53.87 (11.00) | - | - | 50.75 (11.85) | - | 49.61 (14.17) | 47.12 (11.54) | 47.25 (7.56) | 52.63 (8.78) | 46.14 (10.26) | 53.26 (10.39) | 50.06 (10.26) | 50.80 (10.11) | 53.09 (8.41) | **.026** | .04 |
| Stroop-color accuracy T-score | 54.94 (9.11) | - | - | 51.45 (11.30) | - | 47.91 (15.13) | 46.91 (11.35) | 46.66 (9.09) | 52.32 (8.75) | 44.61 (14.21) | 52.66 (10.11) | 50.23 (9.29) | 50.80 (9.37) | 52.78 (7.91) | **.002** | .06 |
| VSLT 4x4 T-Score | 49.38 (8.07) | - | - | 50.96 (9.21) | - | 53.95 (12.08) | 51.76 (11.30) | 48.31 (8.30) | 48.55 (11.87) | 49.04 (10.31) | 49.68 (10.30) | 50.71 (8.82) | 52.28 (9.65) | 51.03 (10.06) | .558 | .02 |
| Global T-Score | 51.39 (6.17) | - | - | 51.54 (7.95) | - | 50.45 (7.44) | 49.57 (6.58) | 47.37 (6.99) | 50.28 (7.46) | 46.49 (7.70) | 51.62 (6.98) | 50.28 (6.91) | 51.75 (6.68) | 51.95 (6.03) | **.021** | .04 |

^1^Mean (SD); ANOVA used to examine clinical site differences in performance; Effect size method= η²

LA=Los Angeles; DC=Washington DC; SF=San Francisco; NW=Northwestern; CC=Cook County; BHAM=Birmingham; Balti=Baltimore; Pitt=Pittsburgh

**Supplemental Table 26**. Cohort differences in cognition in men without HIV.

|  | **MACS**  N = 398^1^ | **WIHS**  N = 0^1^ | **MWCCS**  N =135^1^ | ***P*-value** | ***d*** |
| --- | --- | --- | --- | --- | --- |
| TMT-A total time to completion(ms) | 17,463 (8,190) | - | 18,892 (10,073) | .138 | .16 |
| TMT-B total time to completion(ms) | 40,573 (13,992) | - | 45,799 (15,633) | **<.001** | .36 |
| TMT-B minus A(ms) | 23,109 (12,386) | - | 26,907 (13,634) | **.005** | .30 |
| Stroop Accuracy | 28.93 (3.84) | - | 29.11 (5.18) | .709 | .04 |
| Stroop Response Time (ms) | 874.33 (186.32) | - | 859.08 (219.40) | .470 | .08 |
| VSLT 4x4 Correct minus Incorrect | 0.78 (2.47) | - | 0.50 (2.67) | .288 | .11 |
| TMT-A T-score | 51.42 (8.68) | - | 49.65 (9.30) | .054 | .20 |
| TMT-B T-score | 51.52 (9.91) | - | 48.34 (9.81) | **.001** | .32 |
| TMT B - A T-score | 50.41 (8.63) | - | 48.26 (9.18) | **.018** | .24 |
| Stroop-color duration T-score | 51.64 (9.81) | - | 49.27 (10.83) | **.026** | .23 |
| Stroop-color accuracy T-score | 51.70 (9.10) | - | 49.21 (11.19) | **.021** | .26 |
| VSLT 4x4 T-Score | 51.18 (9.77) | - | 50.44 (9.79) | .454 | .07 |
| Global T-Score | 51.44 (6.73) | - | 49.43 (6.81) | **.003** | .30 |

^1^Mean (SD); T-tests were used to examine cohort differences in performance; Effect size method= Cohen’s *d*

**Supplemental Table 27**. Differences in cognition by education level in men without HIV.

|  | **Less than high school**  N =44^1^ | **Completed high school**  N = 75^1^ | **Some College**  N = 116^1^ | **Graduated College**  N = 125^1^ | **>College**  N = 203^1^ | ***P*-value** | **η²** |
| --- | --- | --- | --- | --- | --- | --- | --- |
| TMT-A total time to completion(ms) | 22,126 (11,362) | 20,099 (11,506) | 17,126 (7,054) | 16,757 (7,767) | 17,245 (8,171) | **<.001** | .03 |
| TMT-B total time to completion(ms) | 52,549 (13,838) | 50,875 (14,398) | 42,716 (14,676) | 38,087 (13,602) | 38,894 (13,345) | **<.001** | .12 |
| TMT-B minus A(ms) | 30,423 (13,422) | 30,776 (13,161) | 25,590 (12,130) | 21,329 (12,981) | 21,649 (11,480) | **<.001** | .08 |
| Stroop Accuracy | 28.66 (5.04) | 27.80 (4.74) | 28.88 (4.42) | 29.42 (4.02) | 29.10 (3.90) | .113 | .01 |
| Stroop Response Time (ms) | 882.07 (212.84) | 915.90 (217.45) | 860.53 (187.48) | 847.72 (188.23) | 870.04 (186.75) | .178 | .01 |
| VSLT 4x4 Correct minus Incorrect | -1.27 (1.93) | 0.15 (2.26) | 0.90 (2.75) | 0.65 (2.33) | 1.10 (2.55) | **<.001** | .06 |
| TMT-A T-score | 50.47 (10.56) | 52.27 (10.16) | 51.67 (8.57) | 50.26 (8.53) | 50.57 (8.33) | .452 | .01 |
| TMT-B T-score | 49.39 (10.02) | 50.62 (9.25) | 51.42 (11.02) | 50.12 (10.28) | 50.72 (9.60) | .787 | .00 |
| TMT B - A T-score | 48.52 (9.84) | 48.67 (9.24) | 49.93 (8.74) | 49.89 (9.33) | 50.24 (8.17) | .615 | .00 |
| Stroop-color duration T-score | 52.65 (11.06) | 50.85 (11.71) | 51.26 (10.05) | 50.45 (10.05) | 50.89 (9.51) | .802 | .00 |
| Stroop-color accuracy T-score | 52.85 (11.93) | 50.62 (11.32) | 51.12 (10.49) | 50.33 (9.58) | 50.72 (8.75) | .686 | .00 |
| VSLT 4x4 T-Score | 47.74 (8.01) | 52.43 (9.12) | 52.27 (10.72) | 49.88 (9.63) | 50.53 (9.81) | **.036** | .02 |
| Global T-Score | 50.06 (6.16) | 51.54 (7.12) | 51.65 (7.93) | 50.17 (6.54) | 50.68 (6.39) | .369 | .01 |

^1^Mean (SD); ANOVA used to examine education differences in performance; Effect size method= η²

**Supplemental Table 28**. Clinical site differences in cognition in the total sample.

|  | **Bronx**  N = 225^1^ | **Brooklyn**  N = 279^1^ | **DC**  N = 181^1^ | **SF**  N = 229^1^ | **Chicago-CC** N = 179^1^ | **Chapel Hill**  N = 245^1^ | **Atlanta**  N = 297^1^ | **Miami**  N = 200^1^ | **BHAM**  N = 126^1^ | **Jackson**  N = 131^1^ | **Balti**  N = 253^1^ | **Chicago-NW**  N = 213^1^ | **Pitt/Ohio**  N = 151^1^ | **LA**  N = 228^1^ | ***P*-value** | η² |
| --- | --- | --- | --- | --- | --- | --- | --- | --- | --- | --- | --- | --- | --- | --- | --- | --- |
| TMT-A total time to completion(ms) | 22,112 (13,741) | 23,291 (13,656) | 19,710 (11,642) | 18,535 (10,098) | 18,533 (9,238) | 17,667 (9,128) | 20,252 (11,713) | 22,079 (14,062) | 18,808 (11,269) | 20,433 (12,199) | 17,614 (7,795) | 17,710 (7,700) | 16,280 (7,228) | 17,990 (9,252) | **<.001** | .03 |
| TMT-B total time to completion(ms) | 49,607 (14,449) | 49,245 (14,107) | 45,666 (14,537) | 42,211 (14,708) | 47,716 (14,963) | 43,507 (15,683) | 47,733 (14,726) | 47,068 (15,332) | 42,434 (15,679) | 45,496 (15,416) | 42,289 (14,492) | 42,092 (14,555) | 38,585 (13,688) | 42,940 (14,434) | **<.001** | .04 |
| TMT-B minus A(ms) | 27,487 (15,054) | 26,110 (15,516) | 25,956 (15,169) | 23,676 (13,428) | 29,182 (15,056) | 25,840 (14,857) | 27,481 (15,443) | 24,989 (14,366) | 23,626 (15,884) | 25,063 (13,177) | 24,674 (12,832) | 24,432 (12,689) | 22,305 (11,957) | 24,950 (13,120) | **<.001** | .01 |
| Stroop Accuracy | 27.69 (4.78) | 27.60 (4.02) | 28.84 (3.96) | 28.52 (4.35) | 27.18 (3.81) | 29.22 (4.26) | 28.01 (4.28) | 27.50 (4.69) | 28.98 (4.85) | 28.34 (5.40) | 29.08 (4.25) | 28.14 (4.23) | 29.28 (3.55) | 29.05 (4.13) | **<.001** | .02 |
| Stroop Response Time (ms) | 915.45 (224.07) | 920.43 (186.90) | 875.26 (159.88) | 886.92 (181.94) | 940.00 (180.44) | 838.15 (185.60) | 887.63 (189.23) | 908.54 (218.30) | 808.93 (169.17) | 846.98 (182.79) | 857.41 (185.54) | 913.43 (207.05) | 854.19 (178.34) | 862.84 (178.30) | **<.001** | .03 |
| VSLT 4x4 Correct minus Incorrect | -0.40 (2.15) | -0.41 (2.28) | 0.03 (2.56) | 0.48 (2.55) | -0.38 (2.34) | 0.29 (2.74) | 0.05 (2.47) | -0.04 (2.75) | 0.15 (2.66) | 0.35 (2.80) | 0.38 (2.60) | 0.59 (2.46) | 1.32 (2.66) | 0.69 (2.49) | **<.001** | .03 |
| TMT-A T-score | 49.56 (10.98) | 47.65 (11.00) | 50.60 (10.12) | 51.46 (9.66) | 51.26 (8.98) | 50.84 (8.56) | 49.52 (9.92) | 48.68 (11.18) | 49.55 (10.18) | 47.72 (10.56) | 51.25 (8.08) | 50.76 (7.86) | 52.37 (7.78) | 51.22 (8.69) | **<.001** | .02 |
| TMT-B T-score | 48.95 (9.38) | 48.32 (8.98) | 50.20 (10.22) | 52.55 (10.55) | 48.79 (9.93) | 50.23 (10.30) | 48.49 (9.53) | 49.41 (9.68) | 50.63 (13.12) | 47.44 (11.02) | 50.93 (10.38) | 50.28 (9.91) | 52.41 (9.99) | 50.56 (9.42) | **<.001** | .02 |
| TMT B - A T-score | 49.52 (10.69) | 50.23 (11.07) | 49.88 (10.76) | 51.38 (9.37) | 47.65 (10.56) | 49.24 (10.02) | 48.84 (10.80) | 50.64 (10.04) | 50.60 (11.66) | 49.33 (9.70) | 49.64 (8.99) | 49.29 (8.68) | 50.49 (8.28) | 49.61 (8.87) | **.046** | .01 |
| Stroop-color duration T-score | 49.88 (11.07) | 48.22 (9.40) | 51.00 (8.60) | 50.08 (9.91) | 46.29 (9.48) | 50.15 (10.58) | 48.68 (10.16) | 48.71 (10.94) | 50.14 (10.44) | 47.87 (10.09) | 52.85 (10.94) | 48.83 (9.79) | 51.93 (9.25) | 51.45 (9.66) | **<.001** | .02 |
| Stroop-color accuracy T-score | 49.74 (11.10) | 48.36 (9.85) | 51.53 (9.30) | 50.50 (10.39) | 46.62 (9.72) | 50.08 (10.61) | 48.01 (11.22) | 49.05 (10.96) | 48.52 (12.06) | 46.38 (13.79) | 52.23 (10.99) | 49.11 (9.25) | 52.04 (8.55) | 51.31 (9.37) | **<.001** | .02 |
| VSLT 4x4 T-Score | 48.89 (8.44) | 48.21 (9.31) | 49.37 (9.38) | 51.20 (10.03) | 47.84 (8.99) | 49.40 (10.38) | 49.50 (10.01) | 49.50 (10.42) | 48.55 (11.01) | 49.02 (11.17) | 49.90 (10.31) | 49.81 (9.55) | 52.69 (10.37) | 51.10 (9.97) | **<.001** | .02 |
| Global T-Score | 49.32 (6.62) | 48.10 (6.34) | 50.29 (6.63) | 51.32 (7.14) | 48.55 (5.68) | 50.16 (6.84) | 49.05 (6.27) | 49.07 (7.78) | 49.72 (7.67) | 48.01 (7.76) | 51.23 (6.97) | 49.92 (6.68) | 52.35 (6.58) | 51.08 (6.71) | **<.001** | .03 |

^1^Mean (SD); ANOVA used to examine clinical site differences in performance; Effect size method= η²

LA=Los Angeles; DC=Washington DC; SF=San Francisco; NW=Northwestern; CC=Cook County; BHAM=Birmingham; Balti=Baltimore; Pitt=Pittsburgh

**Supplemental Table 29**. Cohort differences in cognition in the total sample.

|  | **MACS**  N = 808^1^ | **WIHS**  N = 1,185^1^ | **MWCCS**  N = 755^1^ | ***P*-value** | **η²** |
| --- | --- | --- | --- | --- | --- |
| TMT-A total time to completion(ms) | 17,588 (8,228) | 20,781(12,422) | 19,306 (10,940) | **<.001** | .01 |
| TMT-B total time to completion(ms) | 41,742 (14,304) | 46,717 (14,793) | 45,613 (15,579) | **<.001** | .02 |
| TMT-B minus A(ms) | 24,167 (12,687) | 25,946 (15,351) | 26,349 (14,407) | **.005** | .00 |
| Stroop Accuracy | 28.85 (4.04) | 28.03 (4.22) | 28.66 (4.70) | **<.001** | .01 |
| Stroop Response Time (ms) | 875.88 (189.01) | 900.21 (185.51) | 868.86 (201.39) | **<.001** | .01 |
| VSLT 4x4 Correct minus Incorrect | 0.67 (2.55) | -0.10 (2.50) | 0.18 (2.56) | **<.001** | .02 |
| TMT-A T-score | 51.21 (8.46) | 49.48 (10.63) | 49.63 (9.79) | **<.001** | .01 |
| TMT-B T-score | 50.92 (9.98) | 49.42 (9.89) | 48.96 (10.21) | **<.001** | .01 |
| TMT B - A T-score | 49.88 (8.86) | 49.97 (11.10) | 49.00 (10.01) | .098 | .00 |
| Stroop-color duration T-score | 50.96 (10.12) | 48.77 (9.65) | 48.63 (10.45) | **<.001** | .01 |
| Stroop-color accuracy T-score | 51.04 (9.78) | 48.77 (10.20) | 48.37 (11.29) | **<.001** | .01 |
| VSLT 4x4 T-Score | 50.90 (10.04) | 49.00 (9.86) | 49.44 (9.67) | **<.001** | .01 |
| Global T-Score | 51.00 (6.89) | 49.17 (6.74) | 49.16 (6.85) | **<.001** | .01 |

^1^Mean (SD); ANOVA used to examine cohort differences in performance; Effect size method= η²

**Supplemental Table 30**. Differences in cognition by education level in the total sample.

|  | **Less than high school**  N = 470^1^ | **Completed high school**  N = 629^1^ | **Some College**  N = 942^1^ | **Graduated College**  N = 322^1^ | **>College**  N = 564^1^ | ***P*-value** | **η²** |
| --- | --- | --- | --- | --- | --- | --- | --- |
| TMT-A total time to completion(ms) | 23,760 (13,858) | 21,243 (12,109) | 18,808 (10,579) | 16,350 (7,671) | 16,819 (7,840) | **<.001** | .05 |
| TMT-B total time to completion(ms) | 52,801 (13,780) | 49,634 (14,103) | 43,916 (14,746) | 38,175 (13,751) | 38,923 (13,494) | **<.001** | .12 |
| TMT-B minus A(ms) | 29,041 (15,226) | 28,477 (14,716) | 25,109 (14,593) | 21,825 (12,452) | 22,104 (12,287) | **<.001** | .04 |
| Stroop Accuracy | 26.87 (4.83) | 27.76 (4.32) | 28.89 (4.10) | 29.61 (4.33) | 29.25 (3.93) | **<.001** | .04 |
| Stroop Response Time (ms) | 946.97 (219.83) | 912.20 (193.63) | 859.44 (175.43) | 837.29 (184.47) | 860.79 (177.87) | **<.001** | .04 |
| VSLT 4x4 Correct minus Incorrect | -0.71 (2.16) | -0.37 (2.37) | 0.33 (2.61) | 0.74 (2.62) | 1.04 (2.56) | **<.001** | .06 |
| TMT-A T-score | 49.90 (11.46) | 50.63 (10.54) | 49.80 (9.91) | 49.85 (8.34) | 49.72 (8.35) | .479 | .00 |
| TMT-B T-score | 49.67 (9.28) | 50.70 (9.43) | 49.69 (10.58) | 48.89 (10.54) | 49.05 (9.98) | **.030** | .00 |
| TMT B - A T-score | 49.74 (11.10) | 50.05 (10.58) | 49.92 (10.52) | 49.00 (8.98) | 49.17 (8.75) | .370 | .00 |
| Stroop-color duration T-score | 49.41 (11.18) | 49.69 (10.40) | 49.79 (9.53) | 49.12 (10.07) | 48.66 (9.69) | .267 | .00 |
| Stroop-color accuracy T-score | 48.78 (12.29) | 49.57 (10.82) | 49.96 (10.10) | 49.05 (10.81) | 48.66 (9.20) | .118 | .00 |
| VSLT 4x4 T-Score | 50.34 (8.80) | 49.91 (9.48) | 49.42 (10.34) | 49.47 (10.57) | 49.19 (10.15) | .341 | .00 |
| Global T-Score | 49.83 (6.98) | 50.23 (6.90) | 49.68 (6.95) | 49.33 (6.97) | 49.16 (6.56) | .079 | .00 |

^1^Mean (SD); Effect size method= η²

**Supplemental Table 31**. Clinical site differences in cognition in women.

|  | **Bronx**  N = 168^1^ | **Brooklyn**  N = 279^1^ | **DC**  N = 180^1^ | **SF**  N = 174^1^ | **Chicago-CC** N = 179^1^ | **Chapel Hill**  N = 132^1^ | **Atlanta**  N = 200^1^ | **Miami**  N = 95^1^ | **BHAM**  N = 63^1^ | **Jackson**  N = 82^1^ | **Balti**  N = 0^1^ | **Chicago-NW**  N = 0^1^ | **Pitt/Ohio**  N = 0^1^ | **LA**  N = 0^1^ | ***P*-value** | η² |
| --- | --- | --- | --- | --- | --- | --- | --- | --- | --- | --- | --- | --- | --- | --- | --- | --- |
| TMT-A total time to completion(ms) | 23,011 (14,493) | 23,291 (13,656) | 19,724 (11,673) | 19,085 (10,827) | 18,534 (9,238) | 18,939 (9,844) | 20,440 (12,425) | 24,411 (15,813) | 20,830 (13,777) | 20,568 (12,966) | - | - | - | - | **<.001** | .02 |
| TMT-B total time to completion(ms) | 50,426 (14,198) | 49,245 (14,107) | 45,739 (14,544) | 42,848 (14,877) | 47,716 (14,963) | 46,325 (15,685) | 47,026 (14,815) | 49,869 (14,364) | 44,825 (15,890.) | 43,419 (15,015) | - | - | - | - | **<.001** | .03 |
| TMT-B minus A(ms) | 27,415 (15,753) | 26,110 (15,516) | 26,015 (15,190) | 23,763 (14,041) | 29,182 (15,056) | 27,386 (15,396) | 26,586 (15,619) | 25,458 (14,741) | 23,995 (16,930) | 22,851 (13,396) | - | - | - | - | **.026** | .01 |
| Stroop Accuracy | 27.35 (4.57) | 27.60 (4.02) | 28.84 (3.96) | 28.26 (4.33) | 27.18 (3.81) | 28.52 (4.12) | 28.33 (4.02) | 27.51 (4.85) | 28.27 (4.79) | 28.53 (4.92) | - | - | - | - | **.005** | .02 |
| Stroop Response Time (ms) | 936.49 (221.98) | 920.43 (186.90) | 875.63 (160.25) | 897.53 (174.17) | 940.00 (180.44) | 868.17 (182.44) | 875.60 (160.80) | 945.89 (233.38) | 830.02 (156.49) | 843.33 (163.80) | - | - | - | - | **<.001** | .03 |
| VSLT 4x4 Correct minus Incorrect | -0.52 (2.04) | -0.41 (2.28) | 0.03 (2.57) | 0.45 (2.50) | -0.38 (2.34) | -0.34 (2.63) | 0.12 (2.42) | -0.38 (2.47) | -0.08 (2.66) | 0.43 (2.84) | - | - | - | - | **<.001** | .02 |
| TMT-A T-score | 49.16 (11.44) | 47.65 (11.00) | 50.62 (10.15) | 51.15 (9.73) | 51.26 (8.98) | 50.48 (9.17) | 49.75 (10.09) | 47.68 (12.38) | 49.49 (11.27) | 48.36 (11.01) | - | - | - | - | **.003** | .02 |
| TMT-B T-score | 48.75 (9.49) | 48.32 (8.98) | 50.18 (10.24) | 52.49 (10.14) | 48.79 (9.93) | 49.74 (11.39) | 49.29 (9.44) | 48.73 (9.01) | 50.77 (12.77) | 49.93 (10.16) | - | - | - | - | **.004** | .02 |
| TMT B - A T-score | 49.72 (11.32) | 50.23 (11.07) | 49.84 (10.78) | 51.61 (9.79) | 47.65 (10.56) | 48.91 (11.01) | 49.63 (10.96) | 51.19 (10.53) | 51.20 (12.42) | 51.52 (9.45) | - | - | - | - | **.040** | .01 |
| Stroop-color duration T-score | 49.40 (11.19) | 48.22 (9.40) | 51.02 (8.62) | 49.39 (9.34) | 46.29 (9.48) | 49.79 (10.61) | 49.50 (9.07) | 47.33 (11.95) | 51.37 (9.67) | 48.94 (9.43) | - | - | - | - | **<.001** | .02 |
| Stroop-color accuracy T-score | 49.38 (10.77) | 48.36 (9.85) | 51.58 (9.31) | 49.65 (10.17) | 46.62 (9.72) | 49.51 (10.75) | 48.94 (9.52) | 48.04 (11.87) | 49.41 (11.19) | 48.40 (11.81) | - | - | - | - | **.004** | .02 |
| VSLT 4x4 T-Score | 48.63 (8.29) | 48.21 (9.31) | 49.38 (9.40) | 51.33 (9.87) | 47.84 (8.99) | 47.82 (10.48) | 49.89 (9.88) | 49.17 (9.93) | 48.68 (10.87) | 49.76 (11.52) | - | - | - | - | **.024** | .01 |
| Global T-Score | 48.99 (6.75) | 48.10 (6.34) | 50.30 (6.65) | 51.09 (6.80) | 48.55 (5.68) | 49.45 (7.27) | 49.61 (6.02) | 48.23 (8.11) | 50.08 (7.45) | 49.25 (7.62) | - | - | - | - | **<.001** | .02 |

^1^Mean (SD); ANOVA used to examine clinical site differences in performance; Effect size method= η²

LA=Los Angeles; DC=Washington DC; SF=San Francisco; NW=Northwestern; CC=Cook County; BHAM=Birmingham; Balti=Baltimore; Pitt=Pittsburgh

**Supplemental Table 32**. Cohort differences in cognition in women.

|  | **MACS**  N = 0^1^ | **WIHS**  N = 1,185^1^ | **MWCCS**  N =305^1^ | ***P*-value** | ***d*** |
| --- | --- | --- | --- | --- | --- |
| TMT-A total time to completion(ms) | - | 20,781 (12,422) | 21,066 (12,409) | .720 | .02 |
| TMT-B total time to completion(ms) | - | 46,717 (14,793) | 48,584 (15,039) | .053 | .13 |
| TMT-B minus A(ms) | - | 25,946 (15,351) | 27,622 (14,726) | .080 | .11 |
| Stroop Accuracy | - | 28.03 (4.22) | 27.98 (4.06) | .866 | .01 |
| Stroop Response Time (ms) | - | 900.21 (185.51) | 896.93 (179.99) | .778 | .02 |
| VSLT 4x4 Correct minus Incorrect | - | -0.10 (2.50) | -0.28 (2.26) | .221 | .07 |
| TMT-A T-score | - | 49.48 (10.63) | 49.24 (10.69) | .718 | .02 |
| TMT-B T-score | - | 49.42 (9.89) | 48.64 (9.84) | .217 | .08 |
| TMT B - A T-score | - | 49.97 (11.10) | 49.06 (10.42) | .181 | .08 |
| Stroop-color duration T-score | - | 48.77 (9.65) | 48.40 (9.63) | .553 | .04 |
| Stroop-color accuracy T-score | - | 48.77 (10.20) | 48.40 (10.14) | .571 | .04 |
| VSLT 4x4 T-Score | - | 49.00 (9.86) | 48.81 (8.79) | .744 | .02 |
| Global T-Score | - | 49.17 (6.74) | 48.77 (6.46) | .344 | .06 |

^1^Mean (SD); T-tests were used to examine cohort differences in performance; Effect size method= Cohen’s *d*

**Supplemental Table 33**. Differences in cognition by education level in women.

|  | **Less than high school**  N = 353^1^ | **Completed high school**  N = 382^1^ | **Some College**  N = 573^1^ | **Graduated College**  N = 35^1^ | **>College**  N = 204^1^ | ***P*-value** | **η²** |
| --- | --- | --- | --- | --- | --- | --- | --- |
| TMT-A total time to completion(ms) | 24,477 (14,542) | 22,308 (13,228) | 19,577 (11,519) | 16,118 (9,743) | 16,785 (8,296) | **<.001** | .04 |
| TMT-B total time to completion(ms) | 53,069 (14,009) | 50,084 (13,704) | 44,547 (14,536) | 37,765 (13,068) | 39,740 (14,166) | **<.001** | .10 |
| TMT-B minus A(ms) | 28,592 (15,992) | 27,891 (15,326) | 24,970 (15,146) | 21,647 (13,676) | 22,955 (13,315) | **<.001** | .02 |
| Stroop Accuracy | 26.39 (4.78) | 27.48 (4.02) | 28.53 (3.89) | 29.41 (2.59) | 29.61 (3.59) | **<.001** | .07 |
| Stroop Response Time (ms) | 961.57 (220.05) | 923.95 (182.84) | 871.91 (163.55) | 842.77 (104.45) | 839.87 (161.49) | **<.001** | .05 |
| VSLT 4x4 Correct minus Incorrect | -0.67 (2.16) | -0.61 (2.29) | 0.12 (2.52) | 0.83 (2.61) | 0.83 (2.55) | **<.001** | .05 |
| TMT-A T-score | 49.53 (11.69) | 50.26 (11.05) | 49.37 (10.00) | 49.84 (9.45) | 49.01 (8.62) | .647 | .00 |
| TMT-B T-score | 49.83 (9.55) | 50.69 (9.30) | 49.51 (10.45) | 47.36 (9.71) | 47.45 (10.42) | **.003** | .01 |
| TMT B - A T-score | 50.27 (11.50) | 50.50 (10.97) | 50.20 (10.84) | 47.97 (9.34) | 48.09 (9.32) | .066 | .01 |
| Stroop-color duration T-score | 49.16 (11.21) | 49.47 (10.26) | 49.07 (8.86) | 45.51 (7.09) | 48.14 (9.52) | .138 | .00 |
| Stroop-color accuracy T-score | 48.35 (12.34) | 49.45 (10.32) | 49.42 (9.57) | 46.43 (6.28) | 48.42 (8.74) | .207 | .00 |
| VSLT 4x4 T-Score | 50.58 (8.81) | 49.23 (9.27) | 48.66 (10.08) | 48.42 (10.67) | 47.18 (10.25) | **.001** | .01 |
| Global T-Score | 49.78 (7.09) | 49.91 (6.78) | 49.15 (6.49) | 47.78 (6.28) | 47.94 (6.56) | **.004** | .01 |

^1^Mean (SD); Effect size method= η²

**Supplemental Table 34**. Differences in cognition by STRAW+10 menopause status in women.

|  | **Premenopausal**  N = 270^1^ | **Perimenopause**  N = 140^1^ | **Post Menopausal**  N = 961^1^ | ***P*-value** | **η²** |
| --- | --- | --- | --- | --- | --- |
| TMT-A total time to completion(ms) | 17,309 (9,658) | 17,455 (9,467) | 22,151 (13,244) | **<.001** | .03 |
| TMT-B total time to completion(ms) | 42,819(14,449) | 41,905 (14,826) | 48,927 (14,595) | **<.001** | .04 |
| TMT-B minus A(ms) | 25,510 (14,276) | 24,450 (14,848) | 26,789 (15,542) | .151 | .00 |
| Stroop Accuracy | 29.56 (4.11) | 29.23 (3.56) | 27.39 (4.11) | **<.001** | .05 |
| Stroop Response Time (ms) | 822.91 (164.57) | 830.54 (136.15) | 931.49 (183.66) | **<.001** | .07 |
| VSLT 4x4 Correct minus Incorrect | 0.40 (2.84) | 0.61 (2.62) | -0.42 (2.27) | **<.001** | .03 |
| TMT-A T-score | 50.44 (9.65) | 50.82 (9.47) | 49.20 (11.07) | .089 | .00 |
| TMT-B T-score | 48.71 (10.01) | 50.46 (10.41) | 49.36 (9.78) | .238 | .00 |
| TMT B - A T-score | 48.74 (10.20) | 49.94 (10.52) | 50.05 (11.25) | .224 | .00 |
| Stroop-color duration T-score | 48.18 (9.65) | 49.06 (8.75) | 48.74 (9.63) | .604 | .00 |
| Stroop-color accuracy T-score | 48.12 (10.13) | 48.63 (9.35) | 48.83 (10.25) | .602 | .00 |
| VSLT 4x4 T-Score | 48.88 (11.16) | 50.20 (10.11) | 48.72 (9.16) | .241 | .00 |
| Global T-Score | 49.05 (6.97) | 50.14 (6.44) | 49.01 (6.61) | .170 | .00 |

^1^Mean (SD); ANOVA used to examine education differences in performance; Effect size method= η²

**Supplemental Table 35**. Clinical site differences in cognition in men.

|  | **Bronx**  N = 57^1^ | **Brooklyn**  N = 0^1^ | **DC**  N = 1^1^ | **SF**  N = 55^1^ | **Chicago-CC** N = 0^1^ | **Chapel Hill**  N = 113^1^ | **Atlanta**  N = 97^1^ | **Miami**  N = 105^1^ | **BHAM**  N = 63^1^ | **Jackson**  N = 49^1^ | **Balti**  N = 253^1^ | **Chicago-NW**  N = 213^1^ | **Pitt/Ohio**  N = 151^1^ | **LA**  N = 228^1^ | ***P*-value** | η² |
| --- | --- | --- | --- | --- | --- | --- | --- | --- | --- | --- | --- | --- | --- | --- | --- | --- |
| TMT-A total time to completion(ms) | 19,494 (10,928) | - | 17,320 | 16,794 (7,139) | - | 16,181 (8,002) | 19,866 (10,137) | 19,970 (11,955) | 16,786 (7,612) | 20,208 (10,922) | 17,614 (7,795) | 17,710 (7,700) | 16,280 (7,228) | 17,990 (9,252) | **.004** | .02 |
| TMT-B total time to completion(ms) | 47,194 (15,034) | - | 32,583 | 40,196 (14,100) | - | 40,216 (15,094) | 49,191 (14,508) | 44,535 (15,802) | 40,043 (15,217) | 48,971 (15,604) | 42,289 (14,492) | 42,092 (14,555) | 38,585 (13,688) | 42,940 (14,434) | **<.001** | .05 |
| TMT-B minus A(ms) | 27,700 (12,898) | - | 15,263 | 23,402 (11,384) | - | 24,034 (14,0556) | 29,325 (14,985) | 24,565 (14,076) | 23,257 (14,893) | 28,763 (12,044) | 24,674 (12,832) | 24,432 (12,689) | 22,305 (11,957) | 24,950 (13,120) | **.003** | .02 |
| Stroop Accuracy | 28.73 (5.28) | - | - | 29.39 (4.34) | - | 30.34 (4.27) | 27.14 (4.83) | 27.47 (4.45) | 29.54 (4.92) | 27.89 (6.47) | 29.08 (4.25) | 28.14 (4.23) | 29.28 (3.55) | 29.05 (4.13) | **<.001** | .04 |
| Stroop Response Time (ms) | 853.43 (220.56) | - | 807.97 | 853.36 (202.65) | - | 803.08 (183.87) | 912.44 (236.39) | 874.75 (198.83) | 787.83 (179.73) | 853.08 (212.50) | 857.41 (185.54) | 913.43 (207.05) | 854.19 (178.34) | 862.84 (178.30) | **<.001** | .04 |
| VSLT 4x4 Correct minus Incorrect | -0.07 (2.43) | - | 0.00 | 0.55 (2.74) | - | 1.04 (2.68) | -0.08 (2.57) | 0.28 (2.96) | 0.38 (2.66) | 0.22 (2.74) | 0.38 (2.60) | 0.59 (2.46) | 1.32 (2.66) | 0.69 (2.49) | **.002** | .02 |
| TMT-A T-score | 50.74 (9.48) | - | 47.16 | 52.43 (9.45) | - | 51.27 (7.81) | 49.04 (9.58) | 49.57 (9.95) | 49.61 (9.06) | 46.64 (9.78) | 51.25 (8.08) | 50.76 (7.86) | 52.37 (7.78) | 51.22 (8.69) | **.004** | .02 |
| TMT-B T-score | 49.54 (9.11) | - | 54.76 | 52.73 (11.83) | - | 50.80 (8.87) | 46.82 (9.53) | 50.02 (10.25) | 50.48 (13.55) | 43.24 (11.23) | 50.93 (10.38) | 50.28 (9.91) | 52.41 (9.99) | 50.56 (9.42) | **<.001** | .04 |
| TMT B - A T-score | 48.93 (8.64) | - | 56.10 | 50.65 (7.95) | - | 49.62 (8.75) | 47.22 (10.34) | 50.15 (9.60) | 50.00 (10.91) | 45.63 (9.07) | 49.64 (8.99) | 49.29 (8.68) | 50.49 (8.28) | 49.61 (8.87) | **.039** | .02 |
| Stroop-color duration T-score | 51.29 (10.67) | - | 47.57 | 52.24 (11.34) | - | 50.59 (10.57) | 47.00 (11.98) | 49.93 (9.84) | 48.91 (11.11) | 46.06 (10.99) | 52.85 (10.94) | 48.83 (9.79) | 51.93 (9.25) | 51.45 (9.66) | **<.001** | .03 |
| Stroop-color accuracy T-score | 50.81 (12.05) | - | 43.58 | 53.15 (10.72) | - | 50.76 (10.45) | 46.09 (13.95) | 49.95 (10.05) | 47.62 (12.91) | 42.98 (16.16) | 52.23 (10.99) | 49.11 (9.25) | 52.04 (8.55) | 51.31 (9.37) | **<.001** | .05 |
| VSLT 4x4 T-Score | 49.65 (8.92) | - | 46.47 | 50.82 (10.61) | - | 51.26 (10.00) | 48.69 (10.26) | 49.79 (10.89) | 48.42 (11.23) | 47.78 (10.56) | 49.90 (10.31) | 49.81 (9.55) | 52.69 (10.37) | 51.10 (9.97) | **.035** | .02 |
| Global T-Score | 50.30 (6.18) | - | 48.99 | 52.05 (8.12) | - | 50.98 (6.22) | 47.89 (6.63) | 49.83 (7.44) | 49.36 (7.93) | 45.93 (7.61) | 51.23 (6.97) | 49.92 (6.68) | 52.35 (6.58) | 51.08 (6.71) | **<.001** | .05 |

^1^Mean (SD); ANOVA used to examine clinical site differences in performance; Effect size method= η²

LA=Los Angeles; DC=Washington DC; SF=San Francisco; NW=Northwestern; CC=Cook County; BHAM=Birmingham; Balti=Baltimore; Pitt=Pittsburgh

**Supplemental Table 36**. Cohort differences in cognition in men.

|  | **MACS**  N = 808^1^ | **WIHS**  N = 0^1^ | **MWCCS**  N =450^1^ | ***P*-value** | ***d*** |
| --- | --- | --- | --- | --- | --- |
| TMT-A total time to completion(ms) | 17,588 (8,228) | - | 18,113 (9,654) | .330 | .06 |
| TMT-B total time to completion(ms) | 41,741 (14,304) | - | 43,599 (15,633) | **.038** | .13 |
| TMT-B minus A(ms) | 24,167 (12,687) | - | 25,486 (14,138) | .101 | .10 |
| Stroop Accuracy | 28.85 (4.04) | - | 29.12 (5.04) | .329 | .06 |
| Stroop Response Time (ms) | 875.88 (189.01) | - | 849.83 (212.79) | **.031** | .13 |
| VSLT 4x4 Correct minus Incorrect | 0.67 (2.55) | - | 0.50 (2.71) | .285 | .06 |
| TMT-A T-score | 51.21 (8.46) | - | 49.90 (9.13) | **.012** | .15 |
| TMT-B T-score | 50.92 (9.98) | - | 49.18 (10.46) | **.004** | .17 |
| TMT B - A T-score | 49.88 (8.86) | - | 48.96 (9.73) | .096 | .10 |
| Stroop-color duration T-score | 50.96 (10.12) | - | 48.78 (10.97) | **<.001** | .21 |
| Stroop-color accuracy T-score | 51.04 (9.78) | - | 48.35 (12.01) | **<.001** | .25 |
| VSLT 4x4 T-Score | 50.90 (10.04) | - | 49.86 (10.21) | .085 | .10 |
| Global T-Score | 51.00 (6.89) | - | 49.43 (7.10) | **<.001** | .22 |

^1^Mean (SD); T-tests were used to examine cohort differences in performance; Effect size method= Cohen’s *d*

**Supplemental Table 37**. Differences in cognition by education level in men.

|  | **Less than high school**  N = 117^1^ | **Completed high school**  N = 247^1^ | **Some College**  N = 369^1^ | **Graduated College**  N = 287^1^ | **>College**  N = 360^1^ | ***P*-value** | **η²** |
| --- | --- | --- | --- | --- | --- | --- | --- |
| TMT-A total time to completion(ms) | 21,598 (11,332) | 19,596 (9,943) | 17,612 (8,810) | 16,378 (7,399) | 16,839 (7,581) | **<.001** | .03 |
| TMT-B total time to completion(ms) | 51,995 (13,091) | 48,938 (14,701) | 42,937 (15,035) | 38,225 (13,853) | 38,460 (13,095) | **<.001** | .10 |
| TMT-B minus A(ms) | 30,397 (12,600) | 29,385 (13,702) | 25,325 (13,707) | 21,847 (12,320) | 21,621 (11,656) | **<.001** | .06 |
| Stroop Accuracy | 28.03 (4.75) | 28.05 (4.72) | 29.33 (4.39) | 29.63 (4.52) | 29.00 (4.11) | **<.001** | .02 |
| Stroop Response Time (ms) | 902.92 (214.11) | 894.03 (208.29) | 840.08 (191.06) | 836.62 (192.07) | 872.65 (185.68) | **<.001** | .02 |
| VSLT 4x4 Correct minus Incorrect | -0.81 (2.18) | 0.00 (2.44) | 0.66 (2.72) | 0.73 (2.63) | 1.16 (2.57) | **<.001** | .05 |
| TMT-A T-score | 50.71 (9.82) | 51.59 (9.18) | 50.87 (9.31) | 49.87 (8.19) | 50.30 (8.04) | .207 | .00 |
| TMT-B T-score | 49.30 (8.82) | 51.00 (9.79) | 50.56 (10.89) | 49.04 (10.65) | 50.23 (9.61) | .158 | .00 |
| TMT B - A T-score | 48.44 (9.14) | 49.26 (9.66) | 49.79 (9.74) | 49.06 (8.94) | 49.91 (8.26) | .483 | .00 |
| Stroop-color duration T-score | 50.86 (11.41) | 50.49 (10.82) | 51.36 (10.46) | 49.54 (10.32) | 49.42 (9.79) | .081 | .01 |
| Stroop-color accuracy T-score | 50.83 (12.20) | 49.88 (11.67) | 51.19 (10.86) | 49.34 (11.22) | 49.29 (9.43) | .106 | .01 |
| VSLT 4x4 T-Score | 49.46 (8.84) | 51.23 (9.71) | 50.79 (10.67) | 49.54 (10.57) | 50.25 (9.98) | .260 | .00 |
| Global T-Score | 50.08 (6.66) | 51.08 (7.03) | 50.89 (7.46) | 49.50 (7.05) | 50.05 (6.40) | **.039** | .01 |

^1^Mean (SD); Effect size method= η²

**Supplemental Figure 1.** Distribution of raw BRACE scores.

**
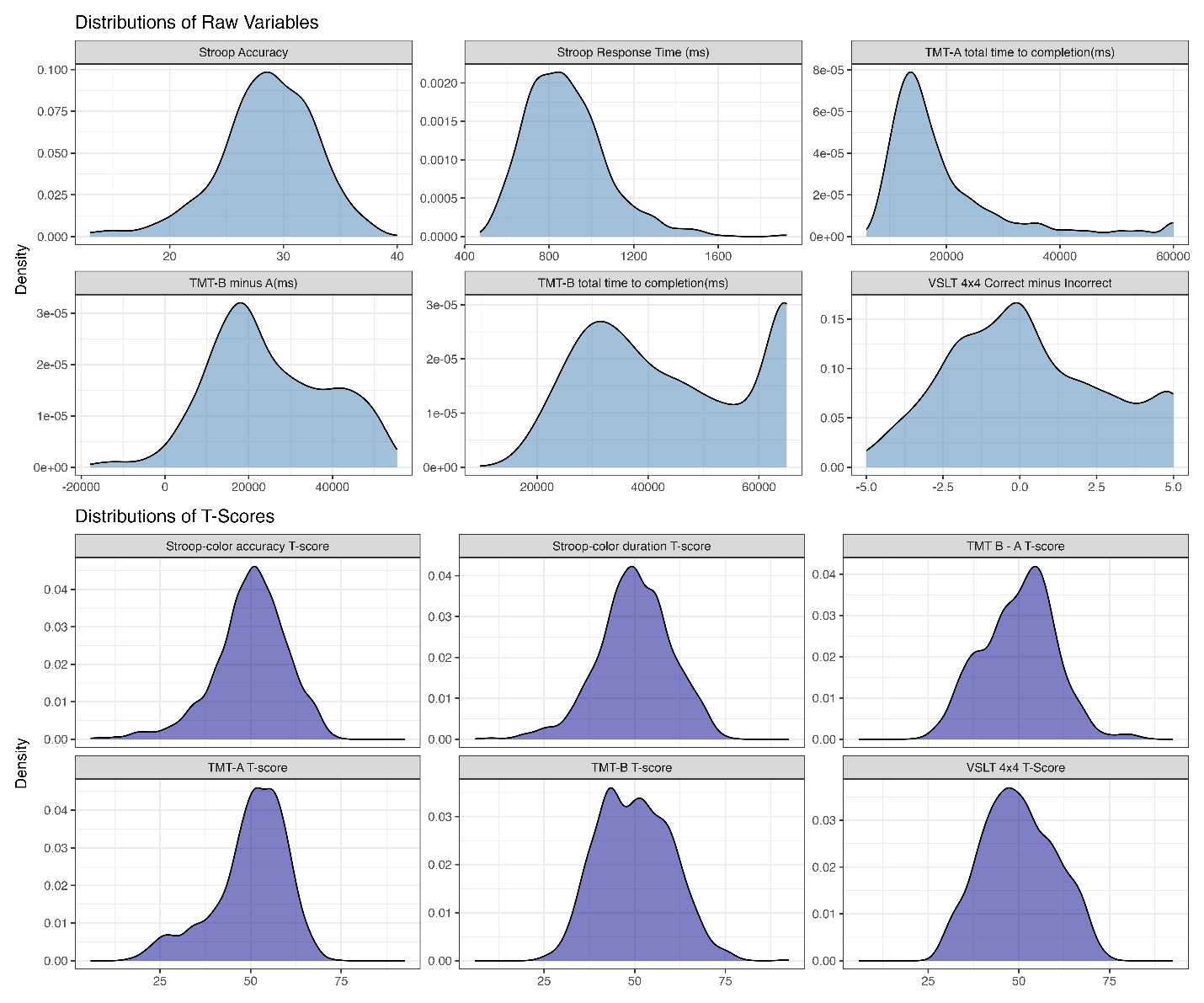
**

**Supplemental Figure 2**. Distribution of Derived T-scores.

**
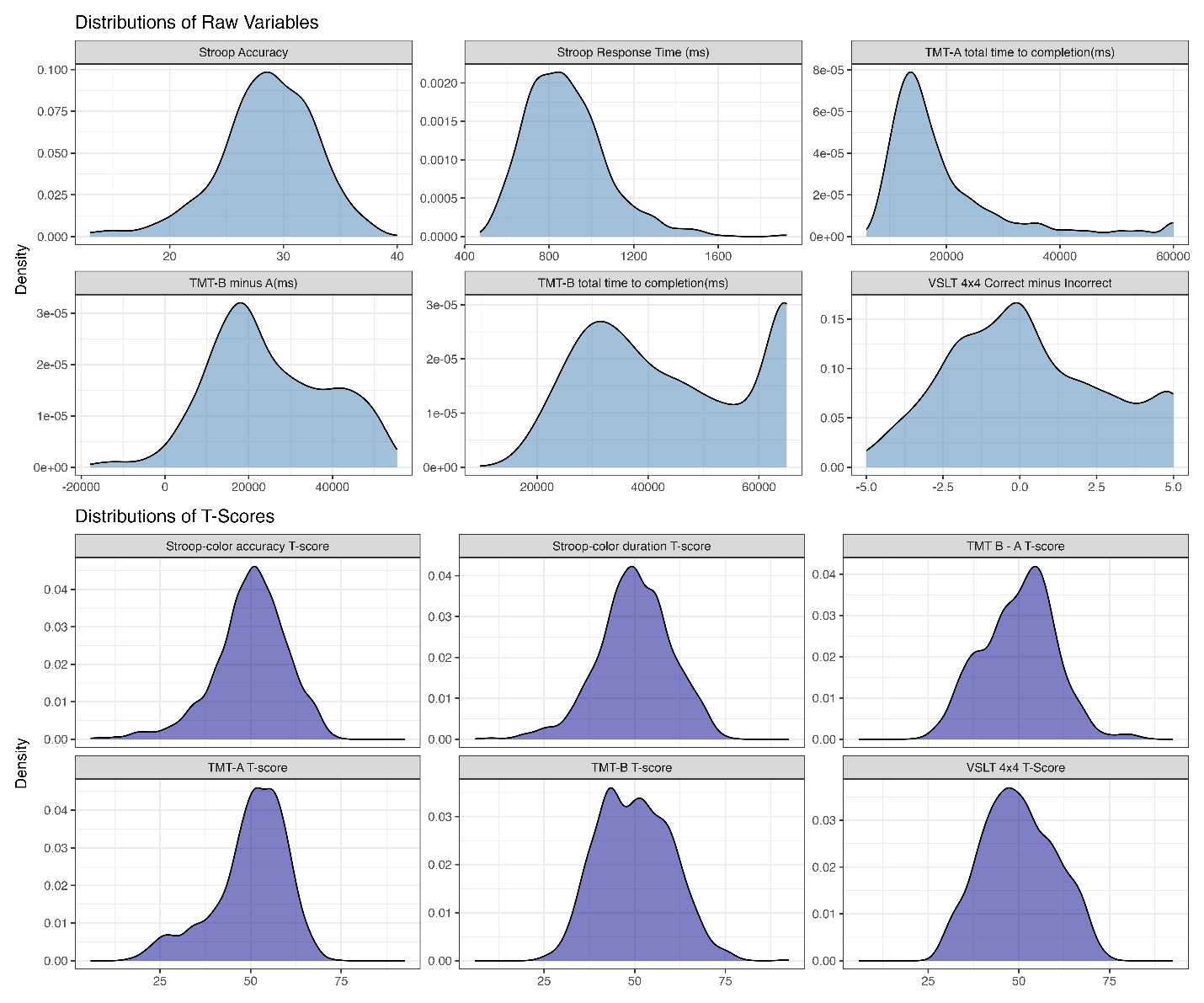
**

**Supplemental Figure 3.** Cognitive correlates among the total sample. The top panel displays associations between all examined variables and performance (raw scores and T-scores). † Denotes raw scores that have been reverse scored so that higher scores indicate better performance. The bottom panel illustrates the direction of and strength of associations: Pearson correlations were used for continuous variables (e.g., age), and point-biserial correlations for binary categorical variables. Significance thresholds are denoted as: ****P*<.001; ***P*<.01; **P*<.05; ns=not significant; Biological sex=male


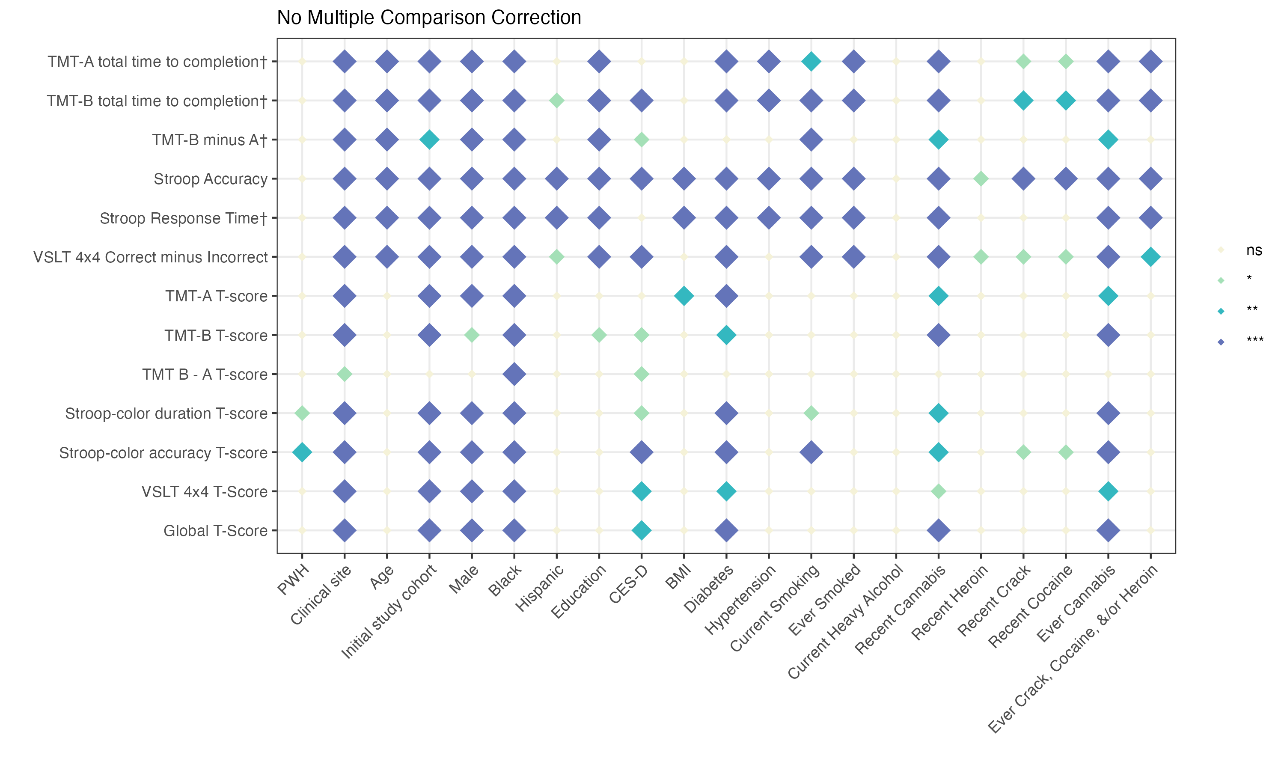


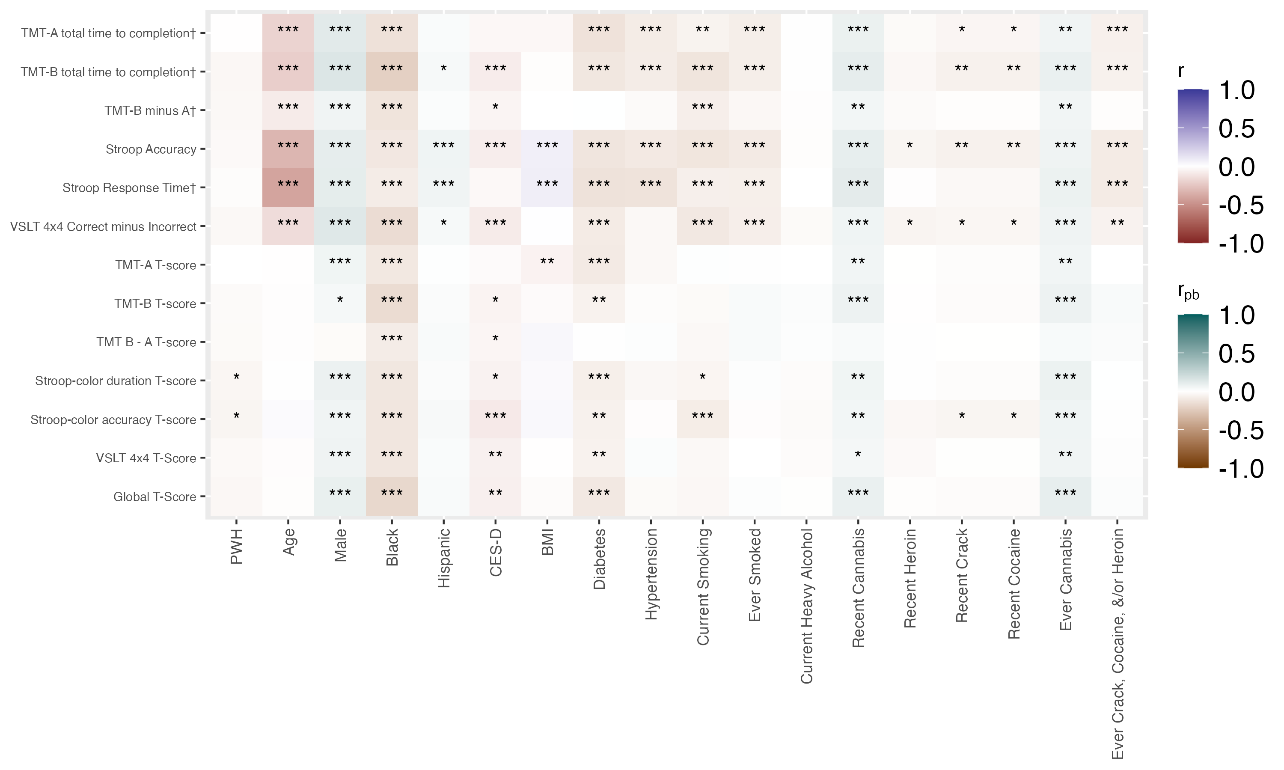


**Supplemental Figure 4.** Cognitive correlates among the total sample of women. The top panel displays associations between all examined variables and performance (raw scores and T-scores). † Denotes raw scores that have been reverse scored so that higher scores indicate better performance. The bottom panel illustrates the direction of and strength of associations: Pearson correlations were used for continuous variables (e.g., age), and point-biserial correlations for binary categorical variables. Significance thresholds are denoted as: ****P* < .001; ***P* < .01; **P* < .05; ns=not significant


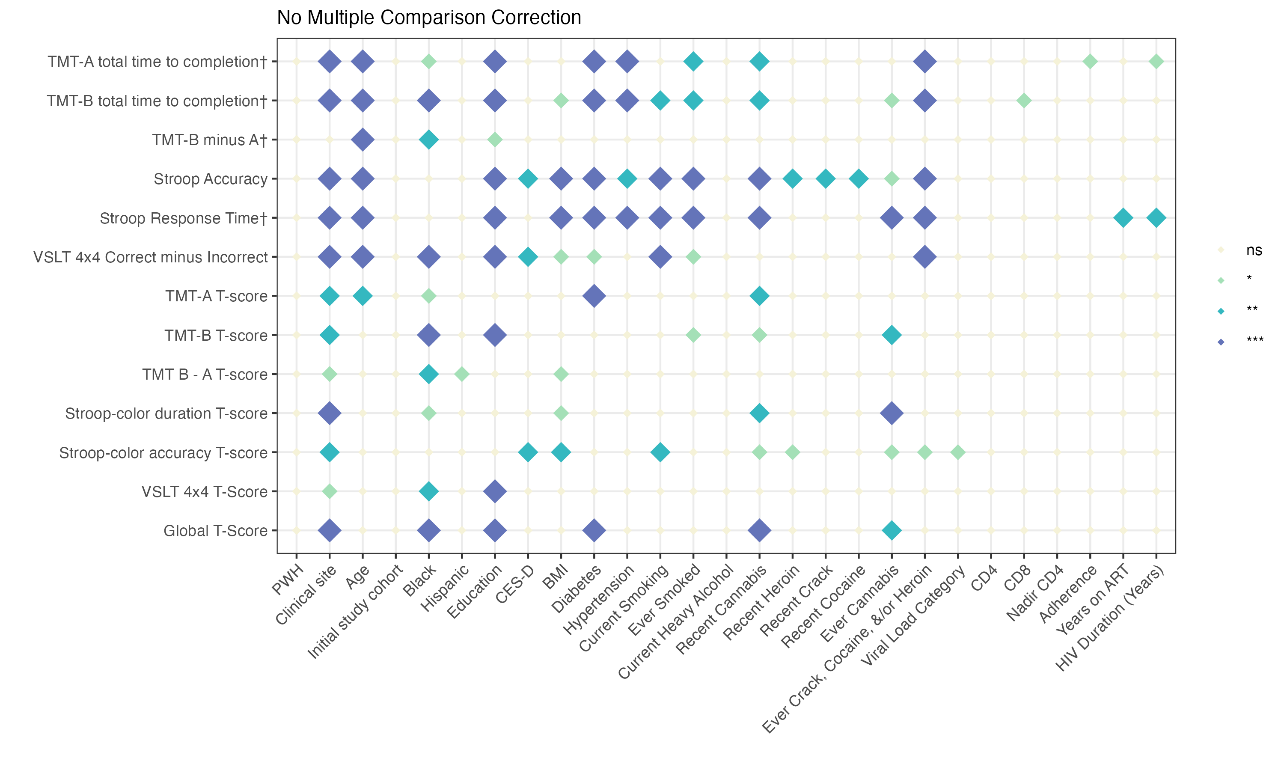


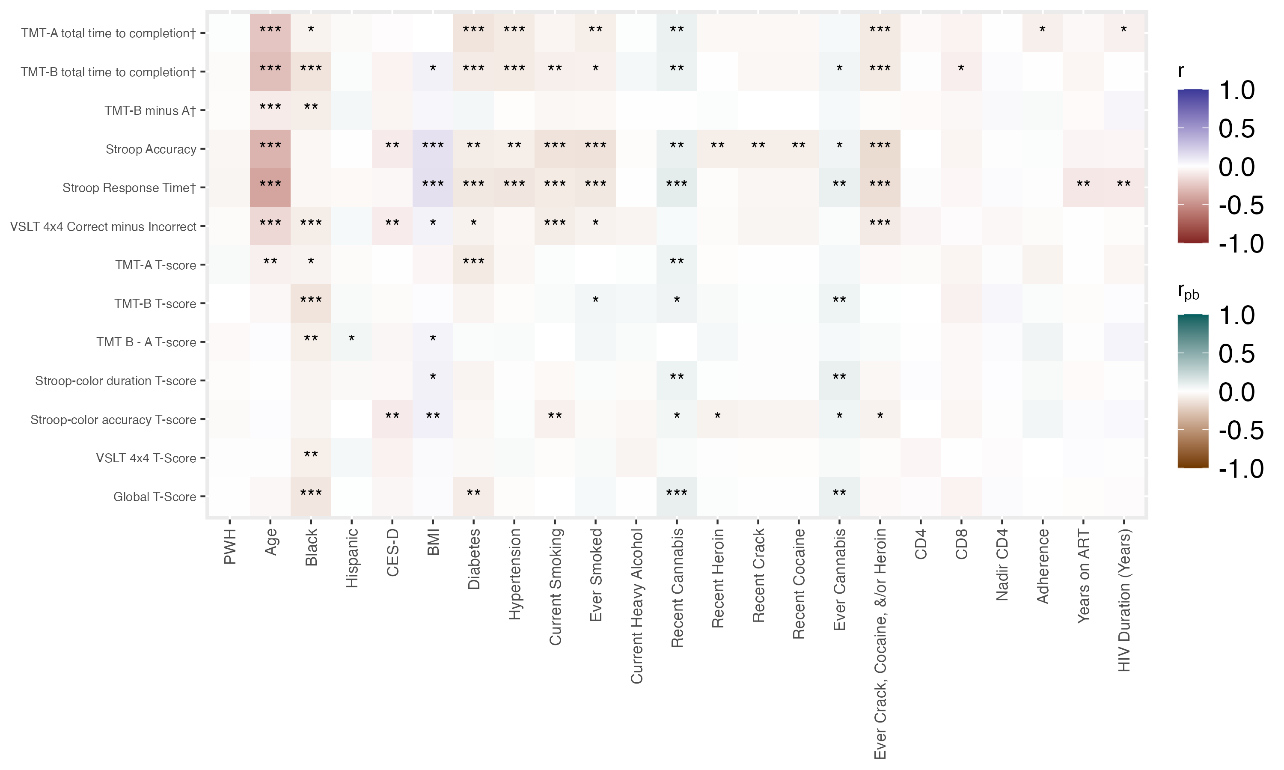


**Supplemental Figure 5.** Cognitive correlates among the total sample of men. The top panel displays associations between all examined variables and performance (raw scores and T-scores). † Denotes raw scores that have been reverse scored so that higher scores indicate better performance. The bottom panel illustrates the direction of and strength of associations: Pearson correlations were used for continuous variables (e.g., age), and point-biserial correlations for binary categorical variables. Significance thresholds are denoted as: ****P* < .001; ***P* < .01; **P* < .05; ns=not significant


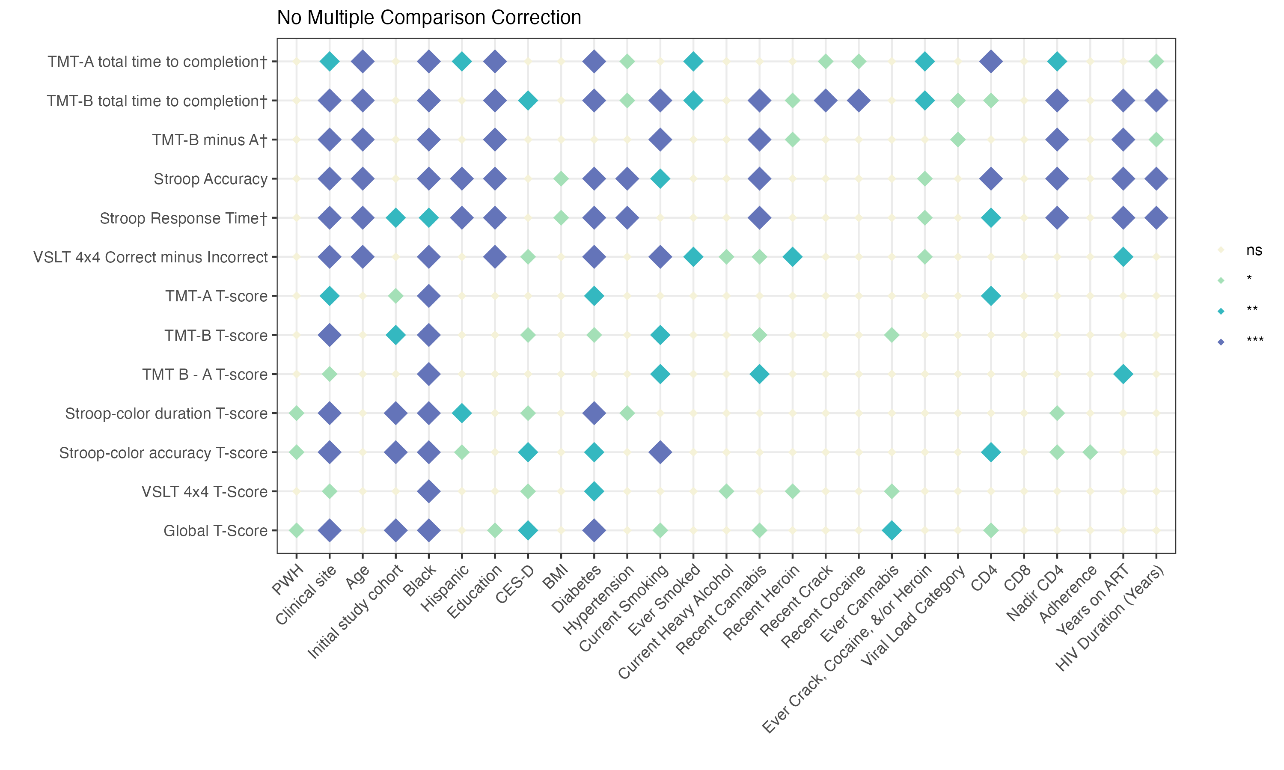


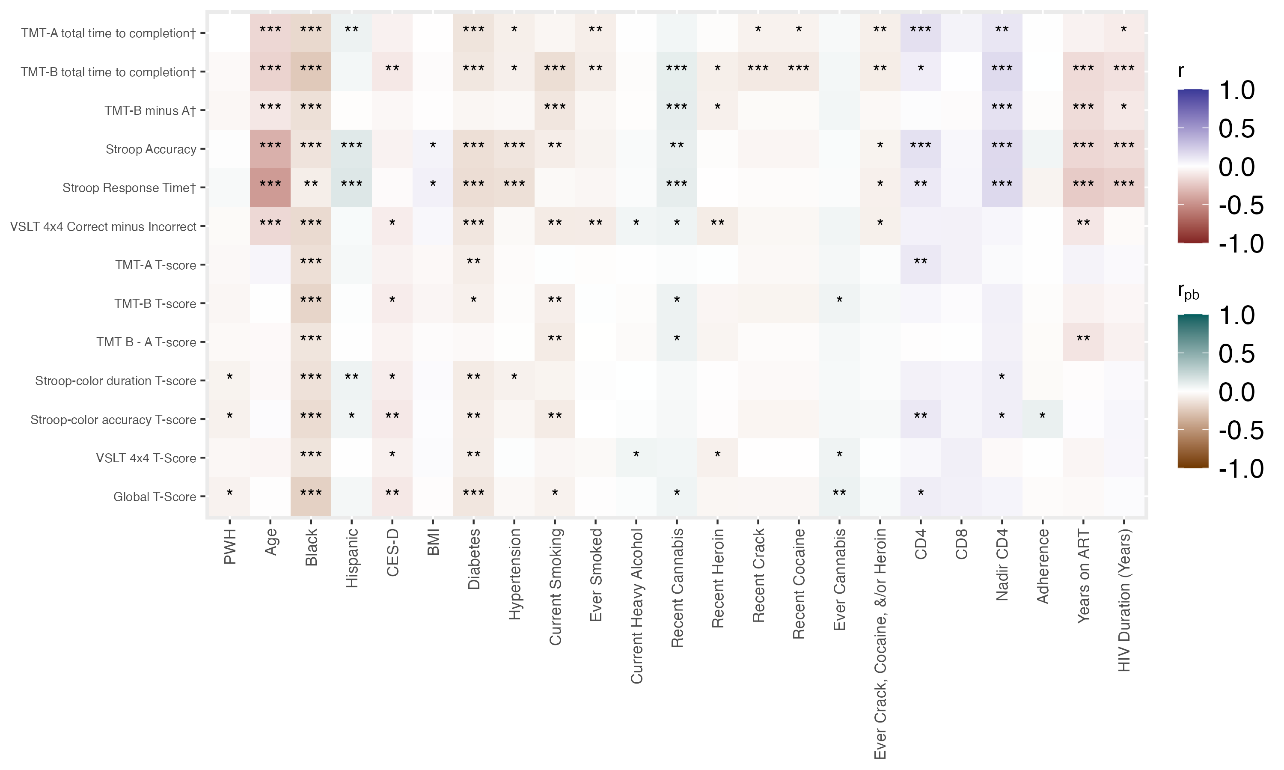

Supplement: Multimedia Appendix 2 [file mental-v13-e70207-s002.docx]
